# Development and validation of a quantitative food frequency questionnaire to assess dietary intake among Lebanese adults

**DOI:** 10.1186/s12937-020-00581-5

**Published:** 2020-07-06

**Authors:** Mireille Harmouche-Karaki, Maya Mahfouz, Jawaher Obeyd, Pascale Salameh, Yara Mahfouz, Khalil Helou

**Affiliations:** 1 Department of Nutrition, Faculty of Pharmacy, Saint Joseph University of Beirut, Lebanon B.P. 11-5076 – Riad el Solh, Beirut, 1107 2180 Lebanon; 2 Clinical and Epidemiological Research Laboratory, Faculty of Pharmacy, Lebanese University, Hadath, Lebanon

**Keywords:** Food frequency questionnaire, Validity, Reproducibility, Macronutrients, Micronutrients, Nutrient intake, 24-h dietary recall, Nutritional epidemiology, Statistical tests, Lebanon

## Abstract

**Background:**

The food frequency questionnaire (FFQ) is the most frequently used method to assess dietary intake in epidemiological studies evaluating diet-disease association. The objective of this study was to validate a FFQ for use among Lebanese adults by evaluating various facets of validity and reproducibility.

**Methods:**

The quantitative 164-items FFQ was validated against the average of six 24-h dietary recalls (DRs) in a sample of 238 Lebanese adults. Reproducibility of the FFQ was assessed by administering it twice within 1 month’ time interval.

**Results:**

Positive statistically significant Pearson correlations were observed in most macro and micronutrients between the FFQ and the six 24-h DRs, ranging from 0.16 to 0.65, with two thirds of the correlation coefficients exceeding 0.3. Energy, gender, and age-adjusted statistically significant Pearson correlation coefficients ranged from 0.14 to 0.64, with two thirds of the coefficients exceeding 0.2. Intakes from the FFQ were mostly higher than those of the 24-h DRs. Mean percent difference between nutrient intakes from both dietary methods decreased remarkably after using energy-adjusted mean intakes. Values were acceptable to good for all macronutrients and several micronutrients. Cross-classification analysis revealed that around 64.3 to 83.9% of participants were classified into the same and adjacent quartile whereas grossly misclassified proportions ranged from 3.7 to 12.2%. Weighted kappa values ranged from 0.02 to 0.36 with most of them exceeding 0.2. In indirect validity analysis, key nutrient mean intakes estimated from the six 24-h DRs were significantly positively associated with tertiles of food groups derived from the FFQ. Bland Altman plots showed that the majority of data points fell within the limits of agreement (LOA) for all nutrients. As for reproducibility analysis, ICC values were all statistically significant ranging from 0.645 to 0.959 and Bland Altman plots confirmed these results.

**Conclusions:**

Based on various aspects of validity and reproducibility, and an extensive range of statistical tests, the present FFQ developed for a Lebanese community is an acceptable tool for dietary assessment and is useful for evaluating diet-disease associations in future studies.

## Introduction

Several epidemiological studies investigate the effect of diet on health and non-communicable diseases such as obesity, diabetes, cancers, as well as neurological, endocrine, and immunological disorders [1]. Such studies require precise methods to evaluate long-term dietary intake in an aim to carry out an extensive dietary assessment. A universal epidemiological method for nutritional assessment does not currently exist [2] and the selection of the adequate instrument depends on the study objectives [3]. Multiple dietary instruments are used to determine nutritional intake [2] and they are divided into objective and self-reported instruments [4]. Objective methods include nutritional biomarkers [3] whereas self-reported dietary instruments are generally divided into short-term methods (24-h dietary recalls (DRs) and food records) and long-term methods (Food Frequency Questionnaire, FFQ) [3]. Each dietary assessment tool has specific advantages and disadvantages [5]. Nutritional biomarkers are the method of choice to assess micronutrient intakes [3]; however, they are relatively expensive and they involve respondents burden [6]. Twenty-four-hour DRs provide reliable quantitative estimates of dietary intakes with no reactivity bias; however, the results may be affected by memory and do not represent the usual dietary intake [6]. Food records - especially weighed food records - have the advantage of being accurate without relying on memory (more accurate portion sizes with no food omission); nevertheless they require relatively high cooperation from participants whose motivation might decrease over time, and intakes can be affected by the process of regular food recording [6]. Time and economic restrictions make the above-mentioned methods unsuitable for use in epidemiological studies [7]. The FFQ is a simple, less invasive, and inexpensive tool [6, 8] that captures the usual dietary intake because it covers a longer period of time [9]. In fact, when evaluating the association between diet and related diseases, measuring food intake over a period of months to years is more valuable than measuring the intake of few days [8]. One main disadvantage of the FFQ is the overestimation of dietary intakes. However, it is the most frequently used method to assess dietary intake in epidemiological studies [2] especially when investigating diet and disease association [10]. Adopting a pre-existing FFQ poses inaccuracies, as the original objectives might not meet the requirements of the current study, and the FFQ yields different results according to different demographic groups [9]. In Lebanon, previous FFQs have been validated for use among children [11] and pregnant women [12] and to assess the intake of antioxidant vitamins [13], and Middle Eastern and Mediterranean food [14]. A new FFQ providing a detailed assessment of a wide array of food and nutrients is needed in order to evaluate the association of diet with health and diseases.

Thus, the aim of the current study was to validate a FFQ for use among Lebanese adults, by investigating various facets of validity and reproducibility. The objectives of the present study were to i) determine the relative validity of the developed FFQ in measuring energy and nutrient intakes as compared to six non-consecutive 24-h DRs ii) compare the means of nutrient intakes across tertiles of food groups obtained by the FFQ iii) evaluate the reproducibility of the FFQ.

## Material and methods

### Study design and participants

A sample of 500 participants was drawn from the university database covering both students and employees, using a stratified random cluster sampling, with a status and sex distribution proportionate to that of the university population per campus. To be included in the study, the participants had to be Lebanese, aged between 18 and 64 years, not having medical conditions or taking medications that affect food intake. Selected participants received a letter by email explaining the procedure of the study. The agreement for participation was requested by phone, 5 days after sending the letter. Out of the 500 participants, 305 agreed to participate in the study. After providing written consent, socio-demographic, anthropometric, and dietary data were collected. Based on the “non-individualized method” [15], also called “recommended method” [16], participants with high or low reported energy intake, i.e. outside the range of 500-3500 kcal/day for women and 800-4000 kcal/day for men, were excluded from the analysis [17]. This resulted in a final sample of 238 participants.

### Development of the FFQ

The quantitative 164-items FFQ was developed by a panel of nutritionists who drafted a pre-final version of the questionnaire that was tested on a representative sample of the target population, composed of 50 students from the university. The respondents completed the FFQ during in-depth interviews where they were asked about its comprehensibility and acceptability [18]. The final version was composed of the following 14 food categories, including culture-specific food items: cereals and grains, dairy products, fruits, vegetables, legumes, meat, fast-food, nuts and seeds, oils and fats, salty snacks, sweets and beverages (hot, alcoholic and non-alcoholic). Portion size was determined according to food servings by the World Health Organization Eastern Mediterranean Region guide based on United States dietary guidelines [19]. During the development phase of the FFQ, respondents were asked about their usually consumed portion size for all food items. Final portion sizes were derived from the most commonly observed ones, reflecting consumption patterns in our target population. A standard portion size was designated for each food item and participants estimated portion sizes by weight, household measures (cups, spoons and plates), and customary packing size [3]. The number of portions consumed was determined and the frequency of portion consumption was recorded per day, week, month or year over the past year. Seasonality of certain food items was accounted for, by adjusting the frequency of consumption for the period of the year during which they were consumed. The FFQ took around 30 min to fill, with this being the usual reported duration [6]. A list of the food items included in the FFQ is available in table S1 in additional file 1.

### Dietary validation analysis

The FFQ was validated against the average of six non-consecutive 24-h DRs. Participants filled 24-h DRs of 3 days (two weekdays and a day of the weekend) repeated twice within one-month interval to obtain a total of six non-consecutive 24-h DRs. The average of six 24-h DRs was used: in order to estimate the usual intake from 24-h DRs and investigate its association with biochemical variables and indicators of health status, it is recommended to collect multiple days. In general, 4 to 5 days are collected as an appropriate compromise between scientific rigor and practicability for assessing energy and macronutrients intake [7]. This method ensures reducing measurement errors [20] and provides more reliable associations [6]. The five steps multiple-pass method [21] proposed by the United States Department of Agriculture was adopted. This method provides a more complete and accurate food recall while making it easier on the participants. The five steps start with a quick list of foods consumed, followed by a list of potentially forgotten foods, time, and occasion of the meals for more precision, detailed quantities and ingredients, ending with a final review [21]. The 24-h DR took 25–30 min to complete, conformingly with the recommendations [7].

**Fig. 1:**
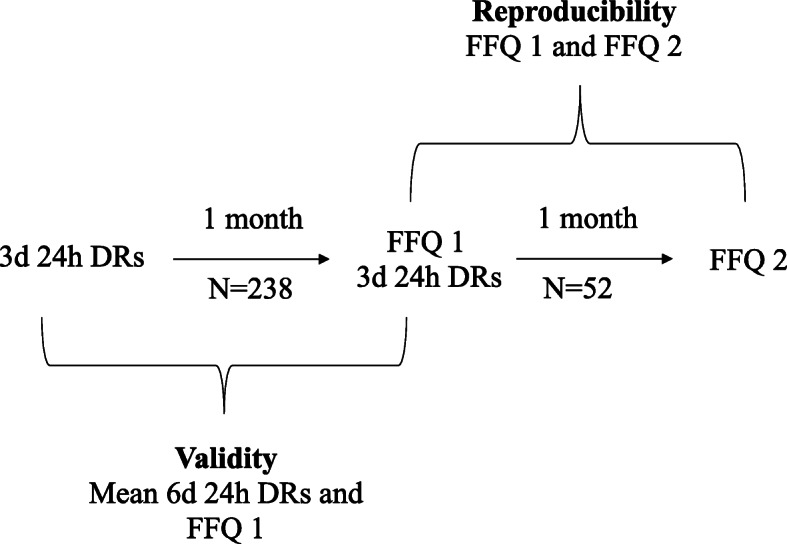
Flow diagram of the study. 3d: three days; 6d: six days; DRs: dietary recalls; FFQ: Food frequency questionnaire.

The reproducibility of the FFQ was tested on a smaller sample from the same target population. A number of 52 participants completed the questionnaire twice within 1 month’ time interval [9]. The period was long enough for the participants to forget their previous responses, but too short for any considerable changes in dietary habits to occur [9]. A period longer than 1 month could lead to seasonal reporting bias [22]. Questionnaires were interviewer-based and not self-administered which increases completion rates and enhances the consistency of the results’ analysis [23, 24]. They were filled by a research assistant who was a trained dietician having experience in both professional and research domains. The flow diagram of the study is shown in Fig. 1.

### Analysis of food consumption data

Nutritional data deriving from the FFQ and the 24-h DRs of 6 days were assessed using the Nutrilog software (Nutrilog SAS, Version 2.30, France). Nutrient composition of the Lebanese traditional dishes was derived from the American University of Beirut (AUB) food composition table [25]. For the rest of the food items that are not exclusively traditional Lebanese, data was extracted from the United States department of agriculture (USDA) nutrient database version 2010 [26] and the French food composition table (Ciqual) version 2008 [27]. These were carefully chosen to reflect our community’s dietary habits in the most accurate way. For products from specific brands, we chose the exact item from the corresponding database. We extracted the nutrient content of each portion based on the amount specified by the abovementioned databases. Hence, nutritional intakes of energy, 18 macronutrients, 11 vitamins, and 10 minerals were retrieved from the FFQ and the average of the six 24-h DRs.

### Data collection

Data were collected regarding age, gender, and crowding index. The latter is defined as the total number of co-residents per household, divided by the total number of rooms, excluding the kitchen and the bathrooms. Anthropometric measurements were taken for the description of the population. Weight and height were measured using a scale and stadiometer (Health o meter professional scale, United States). Body mass index (BMI) was calculated and the participants were classified as overweight or obese if the BMI value ranged between 25 and 29.9 kg/m^2^ and ≥ 30 kg/m^2^ respectively [28].

### Statistical analysis

Frequencies and percentages were used for categorical variables and means (standard deviation SD) were used for quantitative data. Validity of the FFQ as compared to the 24-h DRs was assessed for all nutrients using the Pearson correlation coefficient, with adjustment for energy intake, age, and gender. The analysis was repeated for men and women separately because they have several physiological and behavioral differences that affect distinctively their response to health problems [29]. Mean percent difference was calculated to test the difference between mean nutrient intakes from both dietary instruments (agreement at group level). The same test was done using energy-adjusted nutrient intakes (adjustment was done using the nutrient density method [30]). Mean percent difference = [(FFQ - 24 h recall)/24 h recall]*100. Outcome is judged according to the following criteria: good: 0.0–10.9%; acceptable: 11.0–20.0%; poor: > 20.0% [31]. The distribution of nutrient intakes was categorized into quartiles to test agreement at individual level including chance while weighted kappa was calculated to examine agreement at individual level excluding chance. Interpretation was based on the following cutoffs: good: ≥ 0.61; acceptable: 0.20–0.59; poor: < 0.20 [31]. Cohen’s kappa was calculated to evaluate the agreement between the two measures, based on dichotomized categories of nutrient intakes from both instruments. We used the following cutoffs for results interpretation: almost perfect agreement: 0.81–1.00; substantial: 0.61–0.80; moderate: 0.41–0.60; fair: 0.21–0.40; none to slight: 0.01–0.20; no agreement: ≤ 0 [32]. Indirect validity corresponds to “the extent to which a test measure of a concept agrees with a reference measure of that concept that has a greater degree of demonstrated validity, even if it is not an exact measure of the concept” [33]. It was examined using one-way ANOVA between food categories derived from the FFQ, and nutrient intakes derived from the average of six 24-h DRs. Bland Altman plots were also performed to test the agreement between the two methods; the mean of nutrient intakes between the FFQ and 24-h DRs was plotted against the difference between the two methods. Reproducibility of the FFQ was assessed using the ICC (based on a mean-rating (k = 2), absolute-agreement, 2-way mixed-effects model) as well as the Bland Altman plots. Statistical analyses were performed using IBM SPSS (IBM SPSS Statistics for Windows, Version 20, IBM corp., Armonk, NY).

## Results

General characteristics of the sample are presented in Table 1. A total of 238 participants completed the FFQ and the 6 days 24-h DRs. Over 62.2% were women and most of the participants had a good socio-economic status. Mean age was 27.8 (SD 12.0) years and half of the sample had a high-school education. Prevalence of overweightness and obesity were 31.1 and 4.6% respectively.

**Table 1:** Characteristics of the study sample (*n* = 238)

Characteristics	
Sociodemographic variables	
Gender n(%)^a^	
Male	90 (37.8)
Female	148 (62.2)
Age n(%)	
17–24 years	158 (66.4)
25–39 years	38 (16.0)
40–65 years	42 (17.6)
Crowding index n(%)^a, c^	
< 1	185 (77.7)
≥ 1	53 (22.3)
Education n(%)^a^	
High school or less	120 (50.4)
Bachelor	80 (33.6)
Master’s or Doctor of Philosophy	38 (16.0)
BMI n(%)	
< 18.5 kg/m^2^	16 (6.7)
18.5–24.9 kg/m^2^	137 (57.6)
25–29.9 kg/m^2^	74 (31.1)
≥ 30 kg/m^2^	11 (4.6)

^a^Frequency and percentage were used for categorical variables ^b^Mean average (standard deviation SD) were used for quantitative data ^c^ Crowding index was defined as the total number of co-residents per household, divided by the total number of rooms, excluding the kitchen and the bathrooms

Pearson correlations between FFQ and six 24-h DRs are presented in Table 2. Statistically significant correlations were observed in most macro and micronutrients between the FFQ and the 24-h DRs of 6 days, ranging from 0.16 for monounsaturated fatty acids (%) to 0.65 for alcohol (%). Saturated and polyunsaturated fatty acids (%) did not show significant correlations. Adjustment for age, gender, and energy intake maintained the significant correlations in most nutrients, except for fat (g), saturated fatty acids (g), and polyunsaturated fatty acids (g). Correlation coefficient values decreased after the adjustment except for carbohydrates (CHO) (%), sugars (%CHO), fat (%), monounsaturated fatty acids (%), protein (%), alcohol (%), vitamin E, vitamin C. Adjusted significant correlation coefficients ranged from 0.14 for carbohydrates (g) to 0.64 for alcohol (%). Significant correlation coefficients of vitamins ranged from 0.21 for vitamin D to 0.50 for vitamin B6. Correlations were not significant for vitamin A and riboflavin. Significant correlation coefficients of minerals ranged from 0.28 for copper to 0.45 for phosphorus.

**Table 2:** Comparison of nutrient intakes estimated by the FFQ and the average of six 24-h DRs (*n* = 238)

	Mean nutrient intakes (FFQ)	Mean energy-adjusted nutrient intakes (FFQ)	Mean nutrient intakes (DRs)	Mean energy-adjusted nutrient intakes (DRs)	Mean percent difference^b^	Mean percent difference (energy-adjusted)^b^	Unadjusted Pearson correlation FFQ vs DRs	Adjusted^c,d^ Pearson correlation FFQ vs DRs
Energy Intake (Kcal)	2494.2 (656.9)	2494.2 (656.9)	1736.9 (517.9)	1736.9 (517.9)	50.57	50.57	0.52**	0.32**
Carbohydrates (%)	47.3 (6.0)	47.3 (6.0)	48.8 (7.0)	48.8 (7.0)	−1.49	−1.49	0.27**	0.28**
Carbohydrates (g)^a^	299.4 (81.0)	120.8 (15.7)	211.1 (68.8)	122.1 (19.4)	50.85	1.45	0.49**	0.14*
Sugars (% of total CHO)	27.5 (9.5)	27.5 (9.5)	25.2 (9.3)	25.2 (9.3)	29.39	29.39	0.26**	0.25**
Sugars (g)^a^	80.0 (31.1)	32.7 (11.2)	53.1 (27.2)	30.4 (12.0)	101.93	29.90	0.36**	0.23**
Fat (%)	35.8 (5.3)	35.8 (5.3)	35.2 (6.5)	35.2 (6.5)	5.43	5.43	0.19**	0.19**
Fat (g)^a^	102.2 (32.9)	40.7 (6.0)	68.5 (25.4)	39.3 (7.9)	65.04	11.15	0.42**	0.11
Saturated FA (%)	8.5 (1.8)	8.5 (1.8)	8.0 (2.4)	8.0 (2.4)	16.05	16.05	0.13	0.12
Saturated FA (g)^a^	23.9 (7.8)	9.6 (2.0)	15.9 (7.9)	9.1 (3.7)	81.01	18.48	0.25**	0.08
Monounsaturated FA(%)	13.7 (3.1)	13.7 (3.1)	12.3 (3.4)	12.3 (3.4)	19.34	19.34	0.16*	0.17*
Monounsaturated FA(g)^a^	38.7 (14.1)	15.5 (3.6)	24.1 (10.7)	13.8 (3.9)	87.60	22.83	0.31**	0.15*
Polyunsaturated FA (%)	5.7 (1.4)	5.7 (1.4)	5.5 (1.9)	5.5 (1.9)	16.68	16.68	0.06	0.08
Polyunsaturated FA (g)^a^	16.1 (6.1)	6.4 (1.6)	10.6 (5.3)	6.1 (2.0)	89.07	22.16	0.23**	0.08
Cholesterol^a^	234.8 (116.3)	94.1 (39.9)	129.5 (94.0)	73.9 (46.3)	157.53	68.26	0.38**	0.27**
Proteins (%)	15.7 (3.0)	15.7 (3.1)	15.3 (2.9)	15.3 (2.9)	5.74	5.74	0.31**	0.31**
Proteins (g)^a^	100.1 (33.5)	40.1 (7.5)	66.1 (22.8)	38.3 (8.2)	61.46	7.72	0.50**	0.27**
Fibers^a^	28.9 (9.9)	11.8 (3.3)	18.5 (8.6)	10.8 (4.1)	79.90	21.17	0.35**	0.32**
Alcohol (g)^a^	4.6 (9.45)	1.7 (3.4)	1.8 (5.4)	0.9 (2.6)	155.40	75.69	0.60**	0.59**
Alcohol (%)	1.2 (2.4)	1.2 (2.4)	0.6 (1.8)	0.6 (1.8)	84.00	84.00	0.65**	0.64**
Vitamin A (RAE)^a^	1286.9 (1165.3)	511.5 (405.6)	280.1 (285.8)	161.8 (150.0)	1689.89	756.04	0.05	0.02
Vitamin D^a^	9.4 (1.8)	4.0 ± (1.1)	8.3 (1.6)	5.1 (1.5)	15.82	−17.86	0.21**	0.18**
Vitamin E^a^	14.2 (8.4)	5.7 (3.0)	7.0 (3.7)	4.1 (1.9)	142.61	62.66	0.36**	0.36**
Vitamin C^a^	124.7 (59.8)	51.8 (24.8)	95.1 (67.1)	56.4 (39.4)	94.16	30.05	0.27**	0.28**
Thiamin^a^	2.1 (0.9)	0.8 (0.3)	1.3 (0.5)	0.8 (0.2)	70.32	14.83	0.33**	0.26**
Riboflavin^a^	9.2 (15.0)	3.4 (4.5)	1.6 (1.3)	0.9 (0.6)	563.02	323.04	0.07	−0.02
Niacin^a^	27.3 (12.7)	10.9 (4.2)	19.1 (7.6)	11.2 (3.8)	55.81	5.16	0.37**	0.28**
Pantothenic acid^a^	7.9 (5.2)	3.2 (2.00)	3.9 (2.2)	2.3 (1.3)	125.03	53.73	0.28**	0.27**
Vitamin B6^a^	2.5 (1.2)	1.0 (0.4)	1.5 (0.8)	0.9 (0.4)	97.66	33.31	0.50**	0.49**
Folate^a^	503.4 (259.1)	205.1 (94.1)	338.8 (198.7)	198.4 (102.6)	84.36	23.91	0.47**	0.46**
Vitamin B12^a^	6.5 (4.6)	2.6 (1.6)	3.5 (2.7)	2.0 (1.5)	208.69	102.29	0.30**	0.28**
Magnesium^a^	411.8 (137.5)	167.4 (41.8)	236.2 (96.2)	138.1 (48.4)	95.39	31.22	0.38**	0.33**
Calcium^a^	1046.2 (338.9)	428.1 (124.9)	683.1 (339.4)	398.1 (169.9)	79.97	21.37	0.36**	0.26**
Phosphorus^a^	1583.4 (460.6)	638.3 (102.0)	1014.6 (354.6)	588.6 (131.2)	68.88	12.27	0.45**	0.31**
Potassium^a^	3298.5 (998.3)	1346.5 (314.1)	2279.9 (848.5)	1338.5 (443.8)	62.40	8.03	0.31**	0.25**
Sodium^a^	5666.4 (1049.9)	2352.6 (417.7)	4562.4 (838.5)	2763.1 (588.5)	26.35	−11.97	0.40**	0.15*
Iron^a^	22.5 (11.2)	9.0 (3.8)	15.4 (6.6)	8.9 (3.3)	59.48	8.10	0.39**	0.30**
Zinc^a^	13.9 (8.4)	5.6 (3.1)	7.5 (4.1)	4.4 (2.1)	107.75	42.16	0.31**	0.28**
Copper^a^	1.8 (0.8)	0.7 (0.2)	1.0 (0.4)	0.6 (0.2)	110.00	37.85	0.28**	0.16*
Manganese^a^	4.2 (1.6)	1.5 (0.5)	2.6 (1.1)	1.5 (0.6)	84.29	24.53	0.38**	0.33**
Selenium^a^	86.5 (48.9)	34.7 (16.0)	53.0 (29.2)	30.8 (14.4)	104.76	36.77	0.35**	0.34**

*FFQ* food frequency questionnaire, *DR* dietary recall, *RAE* Retinol Activity Equivalent

^a^intakes were adjusted using the nutrient density method (intake/total energy intake*1000). Energy intake and macronutrients expressed in % total energy intake were not adjusted. ^b^ Relative difference = [(FFQ - 24 h recall)/24 h recall]*100; Percentage difference: Good: 0.0–10.9%; Acceptable: 11.0–20.0%; Poor: > 20.0% **Correlation is significant at the 0.01 level (2-tailed); *. Correlation is significant at the 0.05 level (2-tailed) ^c^Adjusted for energy intake, age and gender; ^d^Energy intake was adjusted for age and gender

Pearson correlations between FFQ and six 24-h DRs for men and women are presented in Table S2 in the additional file 1. When correlations were unadjusted, values were higher for saturated fatty acid (%, g), alcohol (%), niacin, pantothenic acid, and magnesium among men whereas fat (%) and zinc were higher among women. When correlations were adjusted, values were higher for saturated fatty acids (g), monounsaturated fatty acids (g), cholesterol, protein (g), alcohol (%, g), niacin, sodium, and selenium among men whereas fat (%), fibers, vitamin D, zinc, and manganese among women.

The Mean (SD) of energy intake and all nutrient intakes as estimated by the FFQ and the average of six 24-h DRs are presented in Table 2. Mean intakes from the FFQ are mostly higher than those of the 24-h DRs. Mean intakes stratified by gender are available in the additional file (Additional file 1: Table S3). Men generally reported higher intakes than women. Energy-adjusted nutrient intakes are also presented in Table 2. Energy-adjusted mean nutrient intakes stratified by gender are available in the additional file (Additional file 1: Table S4). Women reported higher sugar and micronutrients intakes compared to men, whereas men reported higher cholesterol and alcohol intakes. Mean percent difference was calculated to test the difference between mean nutrient intakes as well as energy-adjusted mean nutrient intakes from both dietary instruments among the total sample. Mean percent difference decreased remarkably after using energy-adjusted mean intakes. See Table 2 for more details.

Table 3 shows the proportions of participants classified in the same category according to the two dietary assessment methods, as well as the corresponding kappa value. Around 49.2–70.6% of participants were classified in the same category. Kappa values ranged from − 0.02 for monounsaturated fatty acids (%) to 0.42 for alcohol (%).

**Table 3:** Agreement of energy, macronutrients, and micronutrients intake assessed by the FFQ and six 24-h DRs

	% in same category	Cohen’s Kappa Coefficient
Energy Intake (Kcal)	67.2	0.35**
Protein (%)	63.9	0.28**
Carbohydrates (%)	63.5	0.27**
Sugars (%)	60.6	0.21**
Fat (%)	53.8	0.08
Saturated FA (%)	56.3	0.13
Monounsaturated FA (%)	49.2	−0.02
Polyunsaturated FA (%)	50.4	0.01
Protein (g)	66.4	0.33**
Carbohydrates (g)	69	0.38**
Sugars (g)	62.2	0.24**
Fat (g)	67.3	0.34**
Saturated FA (g)	62.6	0.25**
Monounsaturated FA (g)	62.2	0.24**
Polyunsaturated FA (g)	62.2	0.24**
Cholesterol	63	0.26**
Fibers	67.6	0.35**
Alcohol (%)	70.6	0.42**
Alcohol (g)	69.3	0.39**
Vitamin A (RAE)	55.4	0.11
Vitamin D	62.2	0.24**
Vitamin E	63	0.26**
Vitamin C	61.4	0.23**
Thiamin	63.8	0.28**
Riboflavin	60.6	0.21**
Niacin	61.4	0.23**
Pantothenic acid	62.2	0.24**
Vitamin B6	65.6	0.31**
Folate	58.4	0.17*
Vitamin B12	58.8	0.18**
Magnesium	63.9	0.28**
Calcium	61.8	0.24**
Phosphorus	62.6	0.25**
Potassium	63	0.26**
Sodium	63	0.26**
Iron	63.8	0.28**
Zinc	63.8	0.28**
Copper	60.6	0.21**
Manganese	63	0.26**
Selenium	62.2	0.24**

*RAE* Retinol Activity Equivalent; ** *p* < 0.01; *p < 0.05

In cross-classification analysis, around 64.3% (% polyunsaturated fatty acids) to 83.9% (alcohol g) were classified into the same and adjacent category. Grossly misclassified proportions ranged from 3.7% (iron) to 12.2% (% polyunsaturated fatty acids). Weighted kappa values ranged from 0.02 (% polyunsaturated fatty acids) to 0.36 (energy intake). Results are shown in Table 4.

**Table 4:** Agreement of energy, macronutrients, and micronutrients intake assessed by the FFQ and six 24-h DRs

	% same + adjacent quartile	% grossly misclassified	Weighted Kappa	Standard Error	95% CI
Energy Intake (Kcal)	83.1	4.2	0.36	0.05	0.27–0.45
Protein (%)	75.6	7.1	0.25	0.05	0.15–0.34
Carbohydrates (%)	73.9	6.7	0.18	0.05	0.09–0.27
Sugars (%)	72.2	8.8	0.18	0.05	0.09–0.28
Fat (%)	69.3	9.2	0.11	0.05	0.02–0.20
Saturated FA (%)	68	11.3	0.10	0.05	0.01–0.19
Monounsaturated FA (%)	65.5	10.1	0.05	0.05	0–0.14
Polyunsaturated FA (%)	64.3	12.2	0.02	0.05	0–0.11
Protein (g)	80.2	3.8	0.33	0.05	0.25–0.42
Carbohydrates (g)	81.9	3.8	0.35	0.05	0.26–0.44
Sugars (g)	74.3	6.7	0.22	0.05	0.13–0.32
Fat (g)	79.1	4.6	0.28	0.05	0.19–0.37
Saturated FA (g)	74.3	8	0.19	0.05	0.10–0.28
Monounsaturated FA (g)	75.1	5.5	0.21	0.05	0.12–0.30
Polyunsaturated FA (g)	73.8	7.6	0.22	0.05	0.12–0.31
Cholesterol	73.9	6.3	0.23	0.05	0.14–0.32
Fibers	79.1	5	0.32	0.05	0.23–0.42
Alcohol (%)	77.2	4.2	0.28	0.05	0.19–0.37
Alcohol (g)	83.9	4.6	0.33	0.04	0.25–0.42
Vitamin A (RAE)	67.6	9.6	–	–	–
Vitamin D	76	6.8	0.24	0.05	0.15–0.33
Vitamin E	76.4	5	0.23	0.05	0.14–0.32
Vitamin C	73	5.9	0.19	0.05	0.10–0.28
Thiamin	76.8	6.7	0.22	0.05	0.13–0.31
Riboflavin	74	7.1	0.18	0.05	0.09–0.28
Niacin	76	5.5	0.24	0.05	0.15–0.33
Pantothenic acid	76.9	5.5	0.27	0.05	0.18–0.36
Vitamin B6	78.6	5.4	0.31	0.05	0.22–0.40
Folate	72.6	6.8	0.17	0.05	0.08–0.26
Vitamin B12	71	7.5	0.19	0.05	0.09–0.28
Magnesium	76.1	6.3	0.27	0.05	0.17–0.36
Calcium	75.3	5	0.23	0.05	0.14–0.33
Phosphorus	77.7	4.6	0.27	0.05	0.18–0.36
Potassium	74.4	5.9	0.22	0.05	0.13–0.32
Sodium	77.3	4.2	0.25	0.05	0.16–0.34
Iron	74.7	3.7	0.26	0.05	0.17–0.35
Zinc	78.1	5.9	0.26	0.05	0.17–0.35
Copper	74.7	6.3	0.22	0.05	0.13–0.32
Manganese	76.5	4.2	0.26	0.05	0.17–0.36
Selenium	76.5	5.9	0.25	0.05	0.16–0.34

*RAE* Retinol Activity Equivalent

The indirect validity of the FFQ is shown in table S5 in additional file 1, where the mean of energy, macronutrients, and micronutrients estimated from six 24-h DRs are presented for a range of food groups classified by the FFQ. Selective associations between a number of food groups and key nutrients are presented in Table 5. Key nutrient mean intakes were positively associated with tertiles of food groups. While refined cereals showed positive associations with CHO and sodium intake, whole-grain cereals were positively associated with fibers intake and several vitamins and minerals that did not show any associations with refined cereals, such as thiamin, B5, B6, zinc, magnesium, etc. Fruits and vegetables showed positive associations with fibers and vitamin C, while folate and other micronutrients were positively associated with cooked green leafy vegetables. Protein and fat intakes that were not among the associations observed with cereals, showed positive associations with legumes and animal products, including red meat, chicken, fish and shellfish, and dairy products. Intakes of vitamins A, D, and E were positively associated with fish and shellfish intake. Iron intake was positively associated with chicken and red meat, fish and shellfish, and legumes. Processed meats and fast food sandwiches were not positively associated with a noteworthy number of micronutrients but showed a positive association with sodium intake. Sugar intake and alcohol intake were positively associated with dessert and alcoholic beverages respectively. See Table 5 and additional file 1: Table S5 for more details.

**Table 5:** Indirect validity: mean (SD) daily nutrient intake as assessed by 24-h DRs of six days according to tertiles of food group consumption (FFQ)

		Tertiles of food group consumption frequency according to FFQ			*p*-value*
		First	Second	Third	
Refined cereals	CHO (g)	**181.2 (58.1)**	**213.0 (64.2)**	**239.2 (71.6)**	0.000
	Sodium (mg)	**4339.7 (722.9)**	4549.0 (810.7)	**4798.7 (917.0)**^**†**^	0.002
Whole Grain cereals	Fibers (g)	**16.7 (9.6)**	**17.2 (6.4)**	**21.7 (8.8)**^**†,‡**^	< 0.001
	Thiamin (mg)	**1.2 (0.5)**	**1.3 (0.5)**	**1.5 (0.5)**^**†,‡**^	0.005
Legumes	Protein (g)	**61.3(18.8)**	**63.8 (20.0)**	**73.0 (27.2)**^**†,‡**^	0.003
	Fibers (g)	**16.1 (7.5)**	18.9 (8.2)	**20.5 (9.5)**^**†**^	0.004
Fruits	Fibers (g)	**16.4 (7.7)**	19.1 (9.7)	**20.1 (8.1)**^**†**^	0.018
	Vitamin C (mg)	**76.8 (53.5)**	**90.3 (59.6)**	**118.8 (79.3)**^**†,‡**^	< 0.001
Total Green leafy vegetables	Fibers	**16.7 (9.4)**	18.4 (9.0)	**20.4 (7.1)**^**†**^	0.024
Cooked green leafy vegetables	Folate (μg)	**294.9 (138.5)**	**320.2 (206.5)**	**393.7 (224.1)**^**†,‡**^	0.004
Chicken and red meat	Protein (g)	**57.1 (17.1)**	**65.9 (19.8)**	**75.2 (26.9)**	< 0.001
	Iron (mg)	**14.2 (5.5)**	**14.6 (7.2)**	**17.2 (6.8)**^**†,‡**^	0.008
Fish and shellfish	Vitamin D	**8.0 (1.3)**	**8.1 (1.0)**	**8.7 (2.2)**^**†,‡**^	0.009
	Selenium (μg)	**43.3 (21.3)**	53.7 (29.7)	**62.0 (32.6)**^**†**^	< 0.001
Dairy Products	Calcium (mg)	**573.5 (284.0)**	680.0 (299.3)	**795.8 (392.0)**^**†**^	< 0.001
Fast food sandwiches	Sodium (mg)	**4299.1 (659.0)**	**4624.4 (795.1)**^**†**^	**4713.2 (964.1)**^**†**^	0.003
Dessert	Sugar (g)	**46.5 (28.5)**	**51.2 (22.1)**	**61.7 (28.8)**^**†,‡**^	0.002
Alcoholic beverages	Alcohol (g)	**0.0 (0.1)**	**1.0 (2.2)**	**4.2 (8.5)**	< 0.001

*One-way ANOVA; *P* < 0.05 is significant; Values in bold show significance

†difference is significant as compared to tertile 1; ‡difference is significant as compared to tertile 2

**Fig. 2:**
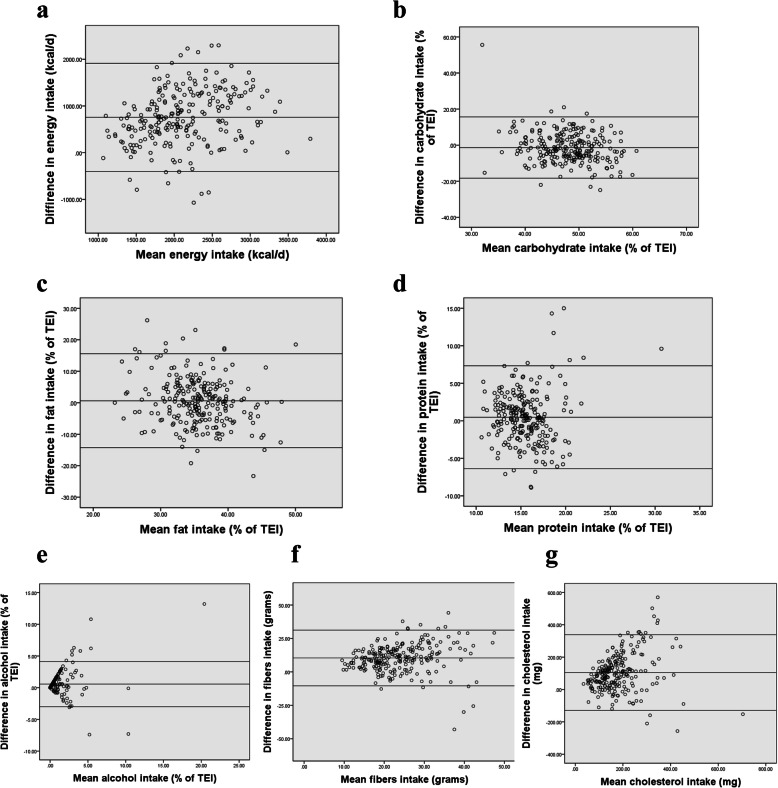
Bland-Altman plots of difference between **a** energy, **b** carbohydrates, **c** proteins, **d** fat,**e** alcohol, **f** fibers, **g** cholesterol, as predicted by the first FFQ and the mean of six 24-hour recalls (*n*=238)

**Fig. 3:**
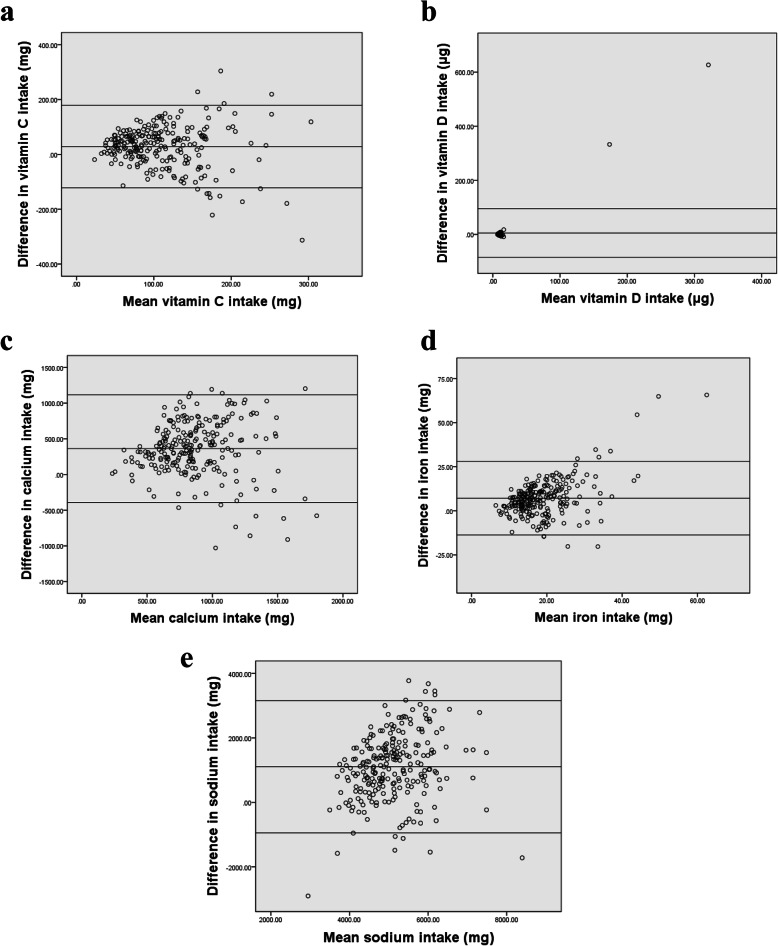
Bland-Altman plots of difference between **a** vitamin C, **b** vitamin D, **c** calcium, **d** iron, and **e** sodium, as predicted by the first FFQ and the mean of six 24-hour recalls (*n*=238)

Bland Altman analysis was conducted by plotting the mean of nutrient intakes between the FFQ and 24-h DRs against the difference between the two methods [31]. Bland Altman plots for selected macronutrients and micronutrients are presented in Figs. 2 and 3. The remaining nutrients are available in additional file 2. The figures showed that most data points fell within the limits of agreement (LOA) for all nutrients. The mean difference and 95% lower and upper limits were 757.27 (− 399.33; 1913.87) for energy intake (kcal/day), − 1.33 (− 18.34; 15.68) for carbohydrate intake (%), 0.62 (− 14.29; 15.54) for fat intake (%), and 0.47 (− 6.38; 7.32) for protein (%) respectively. Results also showed a mean difference of 0.57 (− 3; 4.13) for alcohol (%), 10.39 (− 10.44; 31.21) for fibers (g), and 104.71 (− 129.25; 338.67) for cholesterol (mg). As for micronutrients, results showed a mean difference of 28.54 (− 121.81; 178.89) for vitamin C (mg), 5.15 (− 84.74; 95.05) for vitamin D (mg), 363.15 (− 391.36; 1117.65) for calcium (mg), 7.12 (− 13.69; 27.93) for iron (mg), and 1104 (− 947.16; 3155.15) for sodium (mg). Mean difference, SD as well as LOA values for all nutrients are summarized in Table 6.

**Table 6:** Bland Altman plot analysis for testing the agreement between the FFQ and six 24-h DRs (*n* = 238)

	Mean difference	SD of mean difference	Lower LOA	Upper LOA
Energy Intake	757.27	590.10	−399.33	1913.87
Protein (%)	0.47	3.49	−6.38	7.32
Fats (%)	0.62	7.61	−14.29	15.54
CHO (%)	−1.33	8.68	− 18.34	15.68
Sugars (%CHO)	2.28	11.42	−20.10	24.66
SFA (%)	0.84	6.94	−12.76	14.44
MUFA (%)	1.40	4.21	−6.86	9.66
PUFA (%)	0.16	2.37	−4.48	4.80
Protein (g)	34.05	29.72	−24.19	92.30
Fat (g)	33.70	32.06	−29.13	96.53
CHO (g)	88.32	76.09	−60.82	237.45
Sugar (g)	26.89	33.22	−38.22	92.00
SFA (g)	8.00	9.66	−10.95	26.94
MUFA (g)	14.66	14.85	−14.45	43.77
PUFA (g)	5.48	7.08	−8.40	19.36
Fibers (g)	10.39	10.63	−10.44	31.21
Cholesterol (mg)	104.71	119.37	−129.25	338.67
Alcohol (g)	24.37	331.67	− 625.71	674.44
Alcohol (%)	0.57	1.82	−3.00	4.13
Vitamin A	1004.14	1186.87	− 1322.12	3330.40
Log Vitamin A	0.70	0.48	−0.23	1.63
Vitamin D	5.15	45.86	−84.74	95.05
Vitamin E	14.58	115.33	−211.46	240.62
Vitamin C	28.54	76.71	−121.81	178.89
Thiamin B1	1.51	13.05	−24.07	27.10
Riboflavin B2	7.90	15.71	−22.89	38.70
Niacin	8.02	12.57	−16.62	32.67
B5 (mg)	3.97	5.08	−6.00	13.93
B6 (mg)	3.13	30.84	−57.32	63.58
Folate (ug)	162.39	251.75	− 331.04	655.82
Cobalamin B12 (ug)	3.02	4.63	−6.06	12.09
Magnesium	175.55	135.06	−89.16	440.27
Calcium	363.15	384.95	−391.36	1117.65
Phosphorus	568.86	436.16	−286.02	1423.74
Potassium (mg)	1018.63	1088.89	− 1115.58	3152.85
Sodium (mg)	1104.00	1046.51	−947.16	3155.15
Iron (mg)	7.12	10.62	−13.69	27.93
Zinc (mg)	6.42	8.13	−9.52	22.35
Copper (mg)	0.84	0.82	−0.77	2.45
Manganese (mg)	1.57	1.59	−1.56	4.69
Selenium (ug)	33.53	47.26	−59.10	126.16

*LOA* limit of agreement

### Reproducibility

**Fig. 4:**
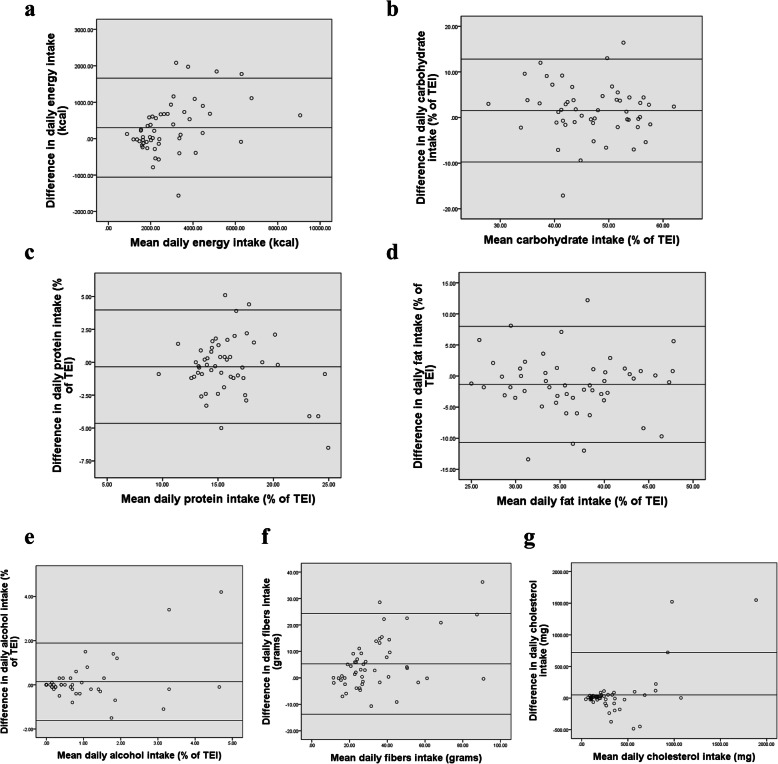
Bland-Altman plots of difference between **a** energy, **b** carbohydrates,** c** proteins, **d** fat,**e** alcohol, **f** fibers, **g** cholesterol, as predicted by the first and second FFQs (*n*=52)

**Fig. 5:**
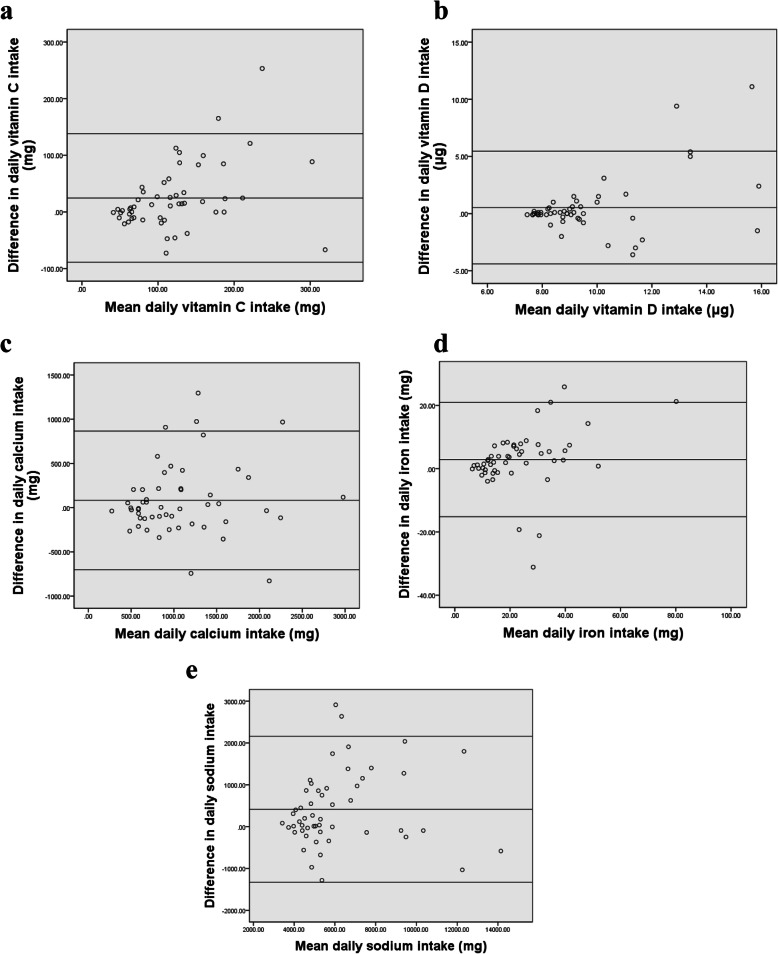
Bland-Altman plots of difference between **a** vitamin C, **b** vitamin D, **c** calcium, **d** iron, and **e** sodium, as predicted by the first and second FFQs (*n*=52)

Participants in the reproducibility and the validity phase had a similar gender distribution (63.5% women, 36.5% men for reproducibility group vs 62.2% women, 37.8% men for validity group), with median age ~ 25.1 years (Min = 18.7, Max = 63.9) mean (SD) = 31.72 (12.08) for reproducibility group vs 22 years (Min = 17, Max = 64) mean (SD) = 27.83 (11.95) for validity group. The two groups also had similar BMI means (mean = 24.5 (5.0) for reproducibility group vs 23.6 (3.7) for validity group). Concerning reproducibility analysis, ICC ranged from 0.645 (vitamin D) to 0.959 (riboflavin). All correlations were statistically significant as shown in Table 7. Bland Altman plots for reproducibility analysis of selected nutrients are presented in Figs. 4 and 5. Plots presenting the rest of the nutrients are available in additional file 2. The mean difference and 95% lower and upper limits for energy intake (kcal/day) were 301.37 (− 1057.81; 1660.54), 1.54 (− 9.74; 12.81) for carbohydrate intake (%), − 1.34 (− 10.66; 7.99) for fat intake (%), and − 0.34 (− 4.65; 3.97) for protein intake (%). Results also showed mean differences of 0.14 (− 1.61; 1.89) for alcohol (%), 5.31 (− 13.71; 24.33) for fibers, and 50.56 (− 619.85; 720.98) for cholesterol. As for micronutrients, mean differences were 24.62 (− 88.8; 138.05) for vitamin C (mg), 0.53 (− 4.4; 5.46) for vitamin D (mg), 82.46 (− 701.1; 866.02) for calcium (mg) 2.88 (− 15.2; 20.97) for iron (mg), and 414.65 (− 1328.22; 2157.53) for sodium (mg). Mean difference, SD as well as LOA values for all nutrients are summarized in Table 8.

**Table 7:** Reproducibility: ICC for nutrient intake as reported in FFQ1 and FFQ2 (*n* = 52)

	Mean (SD) nutrient intakes (FFQ1)	Mean (SD) nutrient intakes (FFQ2)	ICC	Confidence Interval	*p* value
Energy intake (kcal/d)	3074.1 (1768.6)	2772.7 (1486.7)	0.945	0.889–0.971	< 0.001
Protein intake (grams)	126.0 (81.3)	116.8 (75.0)	0.935	0.886–0.963	< 0.001
Protein intake (%)	15.8 (3.1)	16.1 (3.6)	0.879	0.789–0.930	< 0.001
Fat (grams)	127.2 (82.8)	118.8 (72.0)	0.946	0.906–0.969	< 0.001
Fat (%)	35.6 (6.3)	37.0 (6.6)	0.833	0.707–0.904	< 0.001
Carbohydrates (grams)	365.9 (201.2)	316.5 (159.8)	0.913	0.801–0.956	< 0.001
Carbohydrates (%)	47.7 (7.7)	46.1 (8.2)	0.845	0.728–0.911	< 0.001
Sugars (grams)	88.4 (46.5)	77.7 (36.1)	0.826	0.687–0.902	< 0.001
Alcohol (grams)	4.1 (6.0)	3.4 (5.3)	0.852	0.743–0.915	< 0.001
Alcohol (%)	0.9 (1.4)	0.8 (1.0)	0.846	0.733–0.912	< 0.001
Fibers (mg)	36.6 (21.5)	31.3 (16.9)	0.916	0.802–0.959	< 0.001
Cholesterol (mg)	359.8 (461.5)	309.3 (258.7)	0.735	0.541–0.848	< 0.001
Vitamin A (RAE)	1287.4 (1045.9)	1244.0 (1011.3)	0.781	0.618–0.874	< 0.001
Vitamin D (ug)	9.9 (2.9)	9.3 (1.9)	0.645	0.388–0.795	< 0.001
Vitamin E (mg ATE)	16.6 (10.3)	14.6 (7.8)	0.824	0.691–0.900	< 0.001
Vitamin C (mg)	133.1 (77.8)	108.5 (56.9)	0.753	0.544–0.863	< 0.001
Thiamin B1 (mg)	2.3 (1.3)	2.0 (1.1)	0.844	0.715–0.913	< 0.001
Riboflavin B2 (mg)	9.0 (19.6)	8.0 (19.0)	0.959	0.930–0.977	< 0.001
Niacin (mg)	31.2 (20.6)	28.8 (18.3)	0.859	0.756–0.919	< 0.001
Vitamin B5 (mg)	9.1 (5.7)	8.0 (4.6)	0.758	0.580–0.861	< 0.001
Vitamin B6 (mg)	2.8 (1.5)	2.5 (1.3)	0.839	0.714–0.908	< 0.001
Folate (ug)	553.7 (340.9)	474.7 (264.7)	0.849	0.722–0.916	< 0.001
Cobalamin B12 (ug)	6.9 (6.0)	6.6 (5.6)	0.810	0.668–0.891	< 0.001
Magnesium (mg)	482.3 (281.5)	430.3 (224.8)	0.933	0.865–0.965	< 0.001
Calcium (mg)	1118.4 (612.3)	1035.9 (573.3)	0.869	0.773–0.925	< 0.001
Phosphorus (mg)	1979.9 (1130.3)	1828.4 (987.7)	0.940	0.891–0.966	< 0.001
Potassium (mg)	4126.8 (2025.1)	3682.4 (1562.9)	0.900	0.802–0.946	< 0.001
Sodium (mg)	6312.2 (2439.5)	5897.5 (2348.0)	0.958	0.910–0.978	< 0.001
Iron (mg)	24.5 (16.0)	21.7 (12.9)	0.880	0.785–0.932	< 0.001
Zinc (mg)	16.6 (10.7)	14.7 (8.9)	0.824	0.693–0.899	< 0.001
Copper (mg)	2.2 (1.4)	2.0 (1.2)	0.918	0.844–0.955	< 0.001
Manganese (mg)	5.3 (3.4)	4.7 (2.9)	0.951	0.883–0.976	< 0.001
Selenium (ug)	116.6 (99.5)	114.4 (89.5)	0.901	0.827–0.943	< 0.001

*FFQ* food frequency questionnaire, *DR* dietary recall, *RAE* Retinol Activity Equivalent, *ICC* Intra-class correlation coefficient; Model: 2-way mixed model; Type: absolute agreement

**Table 8:** Bland Altman plot analysis for testing the reproducibility of the FFQ (*n* = 52)

	Mean difference	SD of mean difference	Lower LOA	Upper LOA
Energy Intake	301.37	693.46	−1057.81	1660.54
Protein (%)	−0.34	2.20	−4.65	3.97
Fats (%)	−1.34	4.76	−10.66	7.99
CHO (%)	1.54	5.75	−9.74	12.81
Sugar (%CHO)	−0.40	6.63	−13.40	12.59
SFA (%)	0.17	1.88	−3.52	3.86
MUFA (%)	−0.46	2.75	−5.85	4.92
PUFA (%)	−0.28	1.21	−2.64	2.09
Protein (g)	9.21	37.95	−65.17	83.60
Fat (g)	8.41	34.44	−59.09	75.91
CHO (g)	49.35	93.35	− 133.62	232.32
Sugar (g)	10.70	30.98	−50.03	71.43
SFA (g)	3.19	9.79	−15.99	22.37
MUFA (g)	3.66	15.72	−27.16	34.47
PUFA (g)	1.11	6.53	−11.68	13.90
Fibers (g)	5.31	9.70	−13.71	24.33
Cholesterol (mg)	50.56	342.05	−619.85	720.98
Alcohol (g)	0.65	4.07	−7.34	8.63
Alcohol (%)	0.14	0.89	−1.61	1.89
Vitamin A	43.37	876.97	− 1675.49	1762.22
Vitamin D	0.53	2.52	−4.40	5.46
Vitamin E	2.04	6.93	−11.55	15.63
Vitamin C	24.62	57.87	−88.80	138.05
Thiamin B1	0.31	0.86	−1.37	1.99
Riboflavin B2	1.03	7.62	−13.92	15.97
Niacin	2.33	13.63	−24.38	29.04
B5 (mg)	1.08	4.52	−7.78	9.94
B6 (mg)	0.32	1.03	−1.71	2.35
Folate (ug)	79.00	212.30	− 337.11	495.11
Cobalamin B12 (ug)	0.37	4.65	−8.75	9.48
Magnesium	52.06	118.79	−180.78	284.89
Calcium	82.46	399.77	−701.10	866.02
Phosphorus	151.48	490.28	− 809.46	1112.42
Potassium (mg)	444.38	1023.41	− 1561.50	2450.27
Sodium (mg)	414.65	889.22	−1328.22	2157.53
Iron (mg)	2.88	9.23	−15.20	20.97
Zinc (mg)	1.86	7.52	−12.87	16.60
Copper (mg)	0.27	0.69	−1.09	1.62
Manganese (mg)	0.66	1.22	−1.73	3.05
Selenium (ug)	2.19	57.28	−110.07	114.46

*LOA* limit of agreement

## Discussion

Based on various aspects of validity and an extensive range of statistical tests, we demonstrated that the present FFQ developed for a Lebanese community is a useful tool for dietary assessment, when compared to six 24-h DRs. We obtained an acceptable agreement between nutrient intakes of both dietary instruments, given that most participants were correctly classified into the same and adjacent quartiles, with a low level of misclassification. Weighted kappa statistics also showed acceptable results. These findings were further confirmed in the Bland Altman plots and the indirect validity analysis relating nutrient intakes from the 24-h DRs to food groups from the FFQ, indicating a satisfactory agreement between the two methods.

There is no perfect reference method in validation studies. Objective methods such as biochemical indicators are relatively invasive and expensive especially when they aim to test many nutrients. Moreover, biochemical indicators do not exist for some nutrients (total fat, total CHO, total fibers). They are also influenced by dietary factors including day-to-day variation and physiological factors such as nutrient absorption and metabolism, diurnal and menstrual cycles [34]. Diet records also hold several limitations, such as decreased cooperation from the respondents and modification of their dietary intake. Therefore, multiple 24-h DRs appear to be the primary alternative [34], and they are used by most validation studies as a reference method [9].

In validation studies, it is important to cover many aspects of validity. An in-depth literature review carried out in 2015 showed that the mostly used statistical tests in FFQ validation studies were combinations of two to three tests, which may not be sufficient to provide a comprehensive perception of various facets of validity [31]. Moreover, the sole use of correlation analysis is not sufficient in validity studies, as it does not measure agreement between methods [31]. Hence, in the current study, we applied a remarkable number of statistical tests for a more reliable analysis. In addition to correlation analysis, we used percent difference, cross-classification quartiles, weighted kappa statistics, and Bland Altman plots to measure agreement between the two methods, as well as indirect validity analysis between nutrient intakes from 24-h DRs and food consumption categories derived from the FFQ. While correlation coefficient, kappa statistics, and cross-classification assess validity at the individual level, Bland Altman and percent difference do it at the group level [31].

Regarding correlation analyses, a desirable Pearson correlation coefficient generally ranges from 0.5 to 0.7 [34], with coefficients between 0.2 and 0.45 considered acceptable [31]. In the current study, correlation coefficient values fell within the acceptable range, with a good outcome for alcohol (> 0.5). They were similar to some FFQ validation studies [35, 36] and lower than others [14, 37]. Adjusting for factors such as age, gender, and energy intake is very important in validation studies. In line with the present findings, it is unrealistic to obtain high values of correlations coefficients after such an adjustment [34]. However, Pearson correlation coefficient cannot be considered the only determinant of validity as it does not test the level of agreement between the two dietary instruments [31].

Results showed that the FFQ tended to overestimate nutrient intakes as compared to 24-h DRs. This finding is consistent with most of the FFQ validation studies [35, 36, 38, 39]. Possible reasons for this overestimation are the relatively large number of food items participants have to recall while filling the FFQ in comparison with the 24-h DR [9]. We also described mean nutrient intakes by gender; they were generally higher in men than women, which is consistent with previous findings [37].

Regarding the mean percent difference, it was calculated for both crude and energy-adjusted nutrient intakes. The difference remarkably decreased with the energy-adjusted values. It showed acceptable to good results for macronutrients, vitamins such as vitamin D, thiamin, and niacin, and minerals like phosphorus, potassium, sodium, and iron. Given that the FFQ overestimates energy intake, it seemed more plausible to compare intakes when they are energy-adjusted. This allowed evaluating the nutrient composition of the diet as assessed by both dietary instruments, rather than only crude intakes. In future epidemiological studies, especially those evaluating diet-disease associations, it is crucial to consider adjusting for energy intake among other confounding factors; diet-disease associations should not be the sole result of differences in total energy intake between cases and non-cases [30].

Cross-classification of nutrient intakes into quartiles and weighted kappa calculation showed promising results as per the agreement between the dietary instruments. Regarding the quartile categorization, misclassification was less than 10% among most nutrients, while a relatively high proportion of participants were classified into the same or adjacent quartile. Results were similar to previous FFQ validation studies [35, 37, 40]. Moreover, most weighted kappa values fell within the acceptable range (between 0.2 and 0.6) [31] while Cohen’s kappa values reflected fair agreement (between 0.2 and 0.4) [32]. These results are of utmost importance, given that ranking individuals according to their dietary intakes is fundamental in the investigation of diet-disease associations [31].

Bland Altman plots showed a good level of agreement between the two methods. While the positive mean in most plots indicated that the FFQ overestimated intakes, plots show that the majority of data points fell within the LOA around the mean intake.

Indirect validity assesses the relationship between the food consumption categories derived from the FFQ, and the nutrient intakes extracted from the 24-h DRs [41]. This type of analysis has been rarely conducted in previous FFQ validation studies [42, 43]. Results suggested a good indirect validity; intakes of key nutrients significantly increased with the relative tertiles of foods groups that they are usually and logically related to.

Test-retest reliability displays not only the degree of correlation but also the agreement between measurements. In contrast to Pearson correlation coefficient, paired t-test, and Bland Altman plots, ICC is an advisable measure of reliability that assesses both degree of correlation and agreement between two measures [44]. In the current study, the FFQ yielded good to excellent reproducibility according to ICC results [44], similarly to previous studies [12, 14, 36, 45]. Bland Altman analysis for reproducibility confirmed these findings. Moreover, the interval between repeated measurements (1 month) is adequate in order to minimize dietary changes over time as well as the recall of previous answers [9]. In fact, following a time interval longer than 1 month (reaching 3 months), seasonality bias could emerge and affect food reporting during the second administration of the FFQ [22]. Hence, the resulting reproducibility correlation in the present study could be attenuated. Nevertheless, previous studies have adopted time intervals of 2 weeks [43, 46, 47], 3 weeks [12, 38], 4 weeks [11, 36, 48], 4 to 6 weeks [13], and 6 weeks [49].

This is the first FFQ validation study conducted in Lebanon to assess most aspects of validity, for a complete range of macro- and micronutrients. In fact, only a few number of studies worldwide used an extensive number of statistical tests for FFQ validation. Another strength of this study is the number of 24-h DRs (6 days) collected as a reference method for the FFQ validation, which was not common in previous studies. In addition, the sample size which is relatively higher than other validation studies, appears sufficient in the context of deriving useful information on questionnaire validity, when combined with 24-h DRs of 6 days [34].

We acknowledge the present validation study has some limitations. First, the length of the FFQ could have increased the burden on participants, hence impairing the cooperation of the respondents and raising the risk of biased responses and the overestimation of intakes. Therefore, in order to account for this limitation, the FFQ was interviewer-administered which assured a more accurate completion of answers [23, 24]. Despite this limitation, some studies suggested that food lists reaching 200 items could perform better than shorter ones with 100 items, and the resulting respondent burden “does not seem to be a decisive factor for FFQs” [6]. Second, the present sample of a university community is not necessarily representative of the total population; it includes a higher proportion of women, a higher education level, and a younger age distribution. Third, errors usually associated with both dietary instruments should be taken into consideration, including errors related to memory and estimation of energy and nutritional intakes. It would have been preferable to administer the multiple 24-h DRs several times over the period of 1 year. However, this was not possible due to technical and collaboration issues. In order to account for this limitation, we collected multiple 24-h DRs administered twice within an interval of 1 month. Finally, even though food composition databases were carefully chosen to reflect our community’s dietary habits in the most accurate way, the use of multiple food composition tables could still induce a certain level of error.

## Conclusion

In the present study, we performed an extensive range of statistical tests and evaluated various aspects of validity and reproducibility. We demonstrated that the FFQ developed for a Lebanese adult community is an acceptable tool for dietary assessment, namely in the context of nutrients distribution and ranking individuals according to their dietary intake. Hence, it is valuable for use in future epidemiological studies evaluating diet-disease associations. This study also showed that caution must be taken in the quantitative assessment of the diet by accounting for energy intake, in addition to gender and other confounding variables.

## Supplementary information

**Additional file 1: Table S1.** Food items included in the FFQ; **Table S2**. Validity of the FFQ: Pearson correlations between first FFQ and mean of six 24-h DRs, stratified by gender (*n* = 238); **Table S3.** Mean ± SD comparison of nutrient intake estimated by the FFQ and the average of six 24-h dietary recalls, stratified by gender (*n* = 238); **Table S4.** Mean ± SD comparison of energy-adjusted nutrient intake estimated by the FFQ and the average of six 24-h dietary recalls, stratified by gender (*n* = 238); **Table S5.** Indirect validity: mean daily nutrient intake as assessed by 24-h DRs of 6 days according to tertiles of food group consumption (FFQ)

**Additional file 2: Figure S1.** Bland-Altman plots of difference between nutrients as predicted by the first FFQ and the mean of six 24-h recalls (*n* = 238); **Figure S2.** Bland-Altman plots of difference between nutrients as predicted by the first and second FFQs (*n* = 52)

## Data Availability

The datasets used and/or analyzed during the current study are available from the corresponding author on reasonable request.

## Abbreviations

BMI: Body mass index
CHO: Carbohydrates
DRs: Dietary recalls
FA: Fatty acids
FFQ: Food frequency questionnaire
ICC: Intra-class correlation coefficient
LOA: Limits of agreement
