# Supplementary material for: Development and validation of a quantitative food frequency questionnaire to assess dietary intake among Lebanese adults
Source: Nutr J. 2020 Jul 6;19:65. doi: 10.1186/s12937-020-00581-5 (PMC7339409; doi:10.1186/s12937-020-00581-5)
Supplement: Supplementary file 1 — Additional file 1: Table S1. Food items included in the FFQ; Table S2. Validity of the FFQ: Pearson correlations between first FFQ and mean of six 24-h DRs, stratified by gender (n = 238); Table S3. Mean ± SD comparison of nutrient intake estimated by the FFQ and the average of six 24-h dietary recalls, stratified by gender (n = 238); Table S4. Mean ± SD comparison of energy-adjusted nutrient intake estimated by the FFQ and the average of six 24-h dietary recalls, stratified by gender (n = 238); Table S5. Indirect validity: mean daily nutrient intake as assessed by 24-h DRs of 6 days according to tertiles of food group consumption (FFQ) [file 12937_2020_581_MOESM1_ESM.pdf]

Additional file 1

For

Development and validation of a quantitative food frequency questionnaire to assess dietary intake among Lebanese adults

Mireille Harmouche-Karaki[1*], Maya Mahfouz[1], Jawaher Obeyd[1], Pascale Salameh[2], Yara Mahfouz[1], Khalil Helou[1]

[1]Department of Nutrition, Faculty of Pharmacy, Saint Joseph University, Beirut, Lebanon

[2]Clinical and Epidemiological Research Laboratory, Faculty of Pharmacy, Lebanese University, Hadath, Lebanon

Email: Mireille Harmouche-Karaki*: mireille.harmouche@usj.edu.lb; Maya Mahfouz: mzmahfouz5@hotmail.com; Jawaher Obeyd : jawaherobeyd@hotmail.com; Pascale Salameh : psalameh@ul.edu.lb; Yara Mahfouz: yara.mahfouz@hotmail.com; Khalil Helou: khalil.helou@usj.edu.lb

*Correspondence: Mireille Harmouche-Karaki; e-mail: mireille.harmouche@usj.edu.lb; Telephone: +9613593395; Address: Department of Nutrition, Faculty of Pharmacy, Saint Joseph University of Beirut, Lebanon B.P. 11-5076 – Riad el Solh Beirut 1107 2180 – Lebanon

Table of Contents

Content	Page
Table S1. Food items included in the FFQ	3 of 29
Table S2. Validity of the FFQ: Pearson correlations between first FFQ and mean of six 24-hour DRs, stratified by gender (n=238)	7 of 29
Table S3. Mean (SD) comparison of nutrient intake estimated by the FFQ and the average of six 24-hour dietary recalls, stratified by gender (n=238)	10 of 29
Table S4. Mean (SD) comparison of energy-adjusted nutrient intake estimated by the FFQ and the average of six 24-hour dietary recalls, stratified by gender (n=238)	13 of 29
Table S5. Indirect validity: mean daily nutrient intake as assessed by 24-hour DRs of six days according to tertiles of food group consumption (FFQ)	16 of 29

Table S1 Food items included in the FFQ

Bread and cereals
Refined bread
Whole-grain bread
Breakfast cereals
Cereal bars
Oats
Rice and other starches
White rice
Brown rice
White pasta
Brown pasta
Crushed parboiled wheat (Bulgur)
Quinoa
Potato
Corn
Peas
Chestnuts
Pizza
Traditional pies
Meat pies (Sambousik)
Lebanese cheese rolls (Rkakat)
Spinach pies (Fatayer)
Lebanese pizza (Mana'eesh)
Dairy products
Full-fat milk
Low-fat or skimmed milk
Condensed sweetened milk
Full-fat yogurt
Low-fat or skimmed yogurt
Labneh (a traditional product made from milk)
Full-fat or skimmed labneh
Low-fat labneh
White cheese
Yellow cheese
Creamy cheese
Traditional products made from milk:
Areeshe
Kishik
Shankleesh
Fruits and juices
Fruits
Dried fruits
Fresh Juice
Commercial juice
Vegetables

Cooked vegetables
Raw vegetables
Vegetable juice
Home-made vegetable soup
Commercial vegetable soup
Eggplant, traditionally prepared with tahini (traditional sesame cream)

Legumes
Lentils
Kidney beans
Chickpeas (cooked)
Chickpeas (traditionally mixed with tahini)
Fava beans
Green beans

Meats
Chicken (all kinds)
Chicken nuggets
Beef or veal red meat
Sheep meat
Pork meat
Chicken liver (raw and cooked)
Other organ meats (heart, spleen, kidney)
Pork ham
Turkey ham
Other processed meats like mortadella, bologna, salami
Canned meat
Hot dog
Surimi
Bacon
Traditional high-fat meat-based product (Kawarma)
Traditional spicy processed meat (Basterma)
Sausages
Traditional spicy sausages (Sojok)
Kebbeh (minced raw lamb or beef mixed with fine cracked wheat and spices)

Eggs
All kinds of eggs

Fish and shellfish
Fish
Salmon
Sardines
Tuna
Shellfish (all kinds, shrimps, calamari, lobsters)
Oysters
Sushi

Fast-food
Cheeseburger

- Hamburger
- Chicken burger
- Fish burger
- Falafel sandwiches (deep-fried ball, doughnut or patty made from ground chickpeas, fava beans, or both)
- Chawarma sandwich (Sliced marinated grilled meat or chicken usually consumed as a sandwich)

Salty snacks
- Salty crackers
- Popcorn
- Crisps

Condiments and nuts
- Raw nuts
- Fried salty nuts
- Peanut butter

Fats and oils
- Vegetable oil
- Olive oil
- Margarine
- Butter
- Mayonnaise
- Ready-to-eat salad sauce
- Fresh cream
- Olives
- Avocado

Sugars, pastries
- White sugar
- Jam, honey, molasses
- Candies, gum
- Chocolate
- Plain biscuit
- Wafer
- Doughnut
- Croissant
- Cake
- Crêpe / pancake
- Tart
- Milk-based desserts (traditional milk based pudding, custard)
- Ice cream
- Sorbet
- Meghli (dessert based on a floured rice pudding and spiced with anise, caraway, and cinnamon)
- Knefe (cheese pastry soaked in sweet, sugar-based syrup)
- With cheese
- With ashta (fresh Lebanese cream)
- With or without bread

Ghraybeh (Lebanese shortbread cookie)
Halawa (sweet dessert made with sesame cream, sugar and pistachios)
Baklawa (sweet pastry made of layers, filled with chopped nuts and sweetened and held together with syrup or honey)
Maamoul (small shortbread pastries filled with dates, pistachios or walnuts)
Halewet el jibn (rolls of soft, sweet cheese dough stuffed with fresh Lebanese cream ashta and covered with sugar-based syrup)
Znoud el sit (crunchy puff pastry fried and soaked with sugar-based syrup and stuffed with fresh Lebanese cream called ashta)

Non-alcoholic beverages

Water
Carbonated beverages, with sugar
Carbonated beverages, sugar-free
Arabic, Black coffee or espresso (with and/or without sugar)
Coffee with milk (with and/or without sugar)
Coffee with coffee creamer (with and/or without sugar)
Tea
With sugar
Without sugar
Hot chocolate
With sugar
Without sugar

Alcoholic beverages

Beer
Light beer
Wine (all kinds)
Whisky, vodka, gin, arak

Table S2 Validity of the FFQ: Pearson correlations between first FFQ and mean of six 24-hour DRs, stratified by gender (n=238)

	Men (n=90)		Women (n=148)	
	Unadjusted Pearson correlation FFQ vs 24-hour recall of 6 days	Adjusted[a] Pearson correlation FFQ vs 24-hour recall of 6 days	Unadjusted Pearson correlation FFQ vs 24-hour recall of 6 days	Adjusted[a] Pearson correlation FFQ vs 24-hour recall of 6 days
Energy Intake (Kcal)[b]	0.36**	0.31**	0.37**	0.33**
Macronutrients				
Carbohydrates (%)	0.29**	0.30**	0.25**	0.26**
Carbohydrates (g)	0.36**	0.09	0.41**	0.20*
Sugars (% of total CHO)	0.29**	0.27*	0.22**	0.24**
Sugars (g)	0.34**	0.23*	0.34**	0.21*
Lipids (%)	0.12	0.12	0.22**	0.23**
Lipids (g)	0.33**	0.20	0.29**	0.03
Saturated FA (%)	0.22*	0.20	0.07	0.06
Saturated FA (g)	0.36**	0.29**	0.10	-0.06
Monounsaturated FA (%)	0.12	0.10	0.19*	0.19*
Monounsaturated FA (g)	0.22*	0.21	0.21*	0.09
Polyunsaturated FA (%)	-0.04	-0.02	0.14	0.15
Polyunsaturated FA (g)	0.14	0.09	0.20*	0.07
Cholesterol	0.39**	0.36**	0.18*	0.15
Proteins (%)	0.36**	0.36**	0.27**	0.26**
Proteins (g)	0.46**	0.39**	0.23**	0.11

Fibres	0.22*	0.17	0.39**	0.47**
Alcohol (g)	0.63**	0.64**	0.34**	0.31**
Alcohol (%)	0.73**	0.73**	0.36**	0.34**
Micronutrients				
Vitamin A (RAE)	0.01	0.01	0.07	0.03
Vitamin D	0.14	0.14	0.27**	0.29**
Vitamin E	0.35**	0.34**	0.38**	0.38**
Vitamin C	0.23*	0.24*	0.30**	0.31**
Thiamin	0.34**	0.28**	0.29**	0.25**
Riboflavin	0.03	-0.03	-0.02	-0.06
Niacin	0.53**	0.44**	0.20*	0.18*
Pantothenic acid	0.33**	0.29**	0.25**	0.26**
Vitamin B6	0.52**	0.51**	0.49**	0.48**
Folate	0.48**	0.46**	0.45**	0.48**
Vitamin B12	0.27**	0.28**	0.30**	0.28**
Magnesium	0.42**	0.38**	0.21**	0.30**
Calcium	0.31**	0.23*	0.35**	0.29**
Phosphorus	0.41**	0.36**	0.30**	0.27**
Potassium	0.34**	0.28**	0.21*	0.24**
Sodium	0.32**	0.23*	0.31**	0.05
Iron	0.37**	0.24*	0.37**	0.35**

Zinc	0.18	0.13	0.40**	0.41**
Copper	0.19	0.15	0.20*	0.17*
Manganese	0.28**	0.30**	0.38**	0.40**
Selenium	0.32**	0.40**	0.28**	0.28**

FFQ=food frequency questionnaire; DR=dietary recall; RAE= Retinol Activity Equivalent
**. Correlation is significant at the 0.01 level (2-tailed); *. Correlation is significant at the 0.05 level (2-tailed).
[a]Adjusted for energy intake and age
[b]Energy intake was adjusted for age

Table S3 Mean (SD) comparison of nutrient intake estimated by the FFQ and the average of six 24-hour dietary recalls, stratified by gender (n=238)

	Men (n=90)			Women (n=148)		
	FFQ	24-hour dietary recall of six days	p-value	FFQ	24-hour dietary recall of six days	p-value
Energy Intake (Kcal)	2914.9 (611.8)	2061.3 (556.9)	<0.001	2238.4 (543.1)	1539.7 (375.6)	<0.001
Macronutrients						
Carbohydrates (%)	46.4 (5.8)	48.3 (7.2)	0.023	47.9 (6.1)	49.2 (6.9)	0.056
Carbohydrates (g)	343.7 (75.8)	249.5 (76.1)	<0.001	272.5 (72.0)	187.8 (51.7)	<0.001
Sugars (% of total CHO)	25.1 (7.9)	23.7 (9.9)	0.232	28.9 (10.1)	26.1 (8.8)	0.004
Sugars (g)	86.5 (33.9)	59.7 (33.8)	<0.001	76.1 (28.6)	49.1 (21.5)	<0.001
Lipids (%)	35.8 (5.0)	35.3 (6.5)	0.502	35.8 (5.5)	35.1 (6.6)	0.284
Lipids (g)	119.1 (31.6)	81.8 (27.8)	<0.001	91.9 (29.4)	60.3 (19.8)	<0.001
Saturated FA (%)	8.2 (1.7)	7.7 (2.2)	0.070	8.6 (1.8)	8.2 (2.5)	0.086
Saturated FA (g)	27.1 (7.1)	18.0 (7.8)	<0.001	21.9 (7.5)	14.6 (7.8)	<0.001
Monounsaturated FA (%)	13.7 (3.0)	12.6 (3.7)	0.033	13.7 (3.2)	12.1 (3.2)	<0.001
Monounsaturated FA (g)	44.8 (13.4)	29.3 (12.4)	<0.001	35.1 (13.3)	20.9 (8.1)	<0.001
Polyunsaturated FA (%)	5.4 (1.1)	5.6 (2.3)	0.395	5.8 (1.5)	5.4 (1.6)	0.007
Polyunsaturated FA (g)	18.0 (5.6)	12.9 (6.3)	<0.001	15.0 (6.1)	9.3 (4.0)	<0.001
Cholesterol	282.7 (127.5)	170.0 (126.0)	<0.001	205.7 (98.56)	104.8 (54.9)	<0.001
Proteins (%)	16.0 (3.4)	15.5 (3.1)	0.151	15.5 (2.8)	15.1 (2.8)	0.140
Proteins (g)	119.5 (36.0)	79.8 (25.2)	<0.001	88.3 (25.5)	57.7 (16.4)	<0.001
Fibres (g)	32.0 (11.0)	21.4 (9.4)	<0.001	27.0 (8.7)	16.8 (7.6)	<0.001
Alcohol (g)	7.5 (13.8)	2.9 (7.9)	<0.001	2.8 (4.5)	1.0 (2.6)	<0.001

Alcohol (%)	1.8 (3.4)	0.9 (2.5)	0.001	0.8 (1.3)	0.4 (1.1)	<0.001
Micronutrients						
Vitamin A (RAE)	1515.9 (1431.4)	339.4 (383.6)	<0.001	1147.6 (947.3)	244.0 (197.9)	<0.001
Vitamin D	9.7 (2.4)	8.7 (2.0)	0.001	9.2 (1.3)	8.0 (1.3)	<0.001
Vitamin E	14.4 (8.6)	7.9 (3.6)	<0.001	14.1 (8.3)	6.5 (3.7)	<0.001
Vitamin C	125.5 (64.6)	93.9 (68.7)	<0.001	124.2 (56.8)	95.9 (66.3)	<0.001
Thiamin	2.2 (0.9)	1.5 (0.5)	<0.001	2.0 (0.9)	1.2 (0.5)	<0.001
Riboflavin	14.5 (21.7)	1.9 (1.9)	<0.001	5.9 (7.2)	1.4 (0.6)	<0.001
Niacin	30.5 (14.0)	22.0 (6.9)	<0.001	25.4 (11.5)	17.3 (7.4)	<0.001
Pantothenic acid	8.3 (5.8)	4.4 (2.1)	<0.001	7.6 (4.9)	3.6 (2.2)	<0.001
Vitamin B6	2.6 (1.4)	1.6 (0.8)	<0.001	2.4 (1.2)	1.4 (0.8)	<0.001
Folate	541.6 (297.8)	399.5 (232.1)	<0.001	480.2 (230.4)	301.8 (165.6)	<0.001
Vitamin B12	7.5 (5.2)	3.9 (2.6)	<0.001	6.0 (4.1)	3.3 (2.7)	<0.001
Magnesium	466.0 (149.6)	271.3 (106.3)	<0.001	378.8 (118.5)	214.9 (82.8)	<0.001
Calcium	1177.9 (305.3)	743.3 (371.7)	<0.001	966.2 (334.2)	646.5 (313.9)	<0.001
Phosphorus	1836.1 (461.4)	1178.3 (388.4)	<0.001	1429.8 (387.6)	915.0 (291.6)	<0.001
Potassium	3657.7 (1068.5)	2524.9 (907.4)	<0.001	3080.1 (888.0)	2130.9 (776.6)	<0.001
Sodium	6154.1 (1022.4)	4972.5 (966.9)	<0.001	5369.8 (953.9)	4312.9 (633.9)	<0.001
Iron	24.6 (12.2)	17.4 (6.5)	<0.001	21.2 (10.5)	14.1 (6.4)	<0.001
Zinc	15.2 (9.1)	8.9 (4.6)	<0.001	13.2 (7.9)	6.6 (3.5)	<0.001
Copper	2.2 (0.9)	1.2 (0.5)	<0.001	1.6 (0.7)	0.9 (0.4)	<0.001

Manganese	4.7 (1.9)	3.1 (1.3)	<0.001	3.8 (1.3)	2.3 (0.9)	<0.001
Selenium	101.9 (49.9)	65.9 (35.1)	<0.001	77.2 (45.9)	45.1 (21.4)	<0.001

FFQ=food frequency questionnaire; DR=dietary recall; RAE= Retinol Activity Equivalent

Table S4 Mean (SD) comparison of energy-adjusted nutrient intake estimated by the FFQ and the average of six 24-hour dietary recalls, stratified by gender (n=238)

	Men (n=90)		Women (n=148)	
	FFQ	24-hour dietary recall of six days	FFQ	24-hour dietary recall of six days
Energy Intake (Kcal)[a]	2914.9 (611.8)	2061.3 (556.9)	2238.4 (543.1)	1539.7 (375.6)
Macronutrients				
Carbohydrates (%)	46.4 (5.8)	48.3 (7.2)	47.9 (6.1)	49.2 (6.9)
Carbohydrates (g)[a]	118.4 (14.9)	121.5 (19.1)	122.3 (16.1)	122.5 (19.7)
Sugars (% of total CHO)	25.1 (7.9)	23.7 (9.9)	28.9 (10.1)	26.1 (8.8)
Sugars (g)[a]	29.9 (10.8)	28.5 (13.0)	34.4 (11.2)	31.6 (11.3)
Lipids (%)	35.8 (5.0)	35.3 (6.5)	35.8 (5.5)	35.1 (6.6)
Lipids (g)[a]	40.7 (5.7)	39.6 (8.3)	40.7 (6.2)	39.2 (7.7)
Saturated FA (%)	8.2 (1.7)	7.7 (2.2)	8.6 (1.8)	8.2 (2.5)
Saturated FA (g)[a]	9.4 (1.9)	8.7 (2.6)	9.8 (2.1)	9.4 (4.2)
Monounsaturated FA (%)	13.7 (3.0)	12.6 (3.7)	13.7 (3.2)	12.0 (3.2)
Monounsaturated FA (g)[a]	15.4 (3.5)	14.2 (4.4)	15.6 (3.7)	13.5 (3.7)
Polyunsaturated FA (%)	5.4 (1.1)	5.6 (2.3)	5.8 (1.5)	5.4 (1.6)
Polyunsaturated FA (g)[a]	6.1 (1.3)	6.2 (2.3)	6.6 (1.7)	6.0 (1.9)
Cholesterol[a]	96.1 (36.5)	82.3 (58.5)	92.9 (42.0)	68.9 (36.3)
Proteins (%)	16.0 (3.4)	15.5 (3.1)	15.5 (2.8)	15.1 (2.8)
Proteins (g)[a]	40.9 (8.5)	39.2 (9.6)	39.6 (6.9)	37.8 (7.2)
Fibres[a]	11.0 (3.1)	10.5 (3.9)	12.2 (3.3)	11.0 (4.2)
Alcohol (g)[a]	2.5 (4.9)	1.4 (3.5)	1.3 (1.9)	0.6 (1.6)

Alcohol (%)	1.8 (3.4)	0.9 (2.5)	0.9 (1.3)	0.4 (1.1)
Micronutrients				
Vitamin A (RAE)[a]	516.7 (461.7)	465.3 (181.7)	508.3 (369.0)	159.6 (127.5)
Vitamin D[a]	3.5 (1.1)	4.5 (1.4)	4.3 (1.0)	5.5 (1.4)
Vitamin E[a]	4.9 (2.7)	3.9 (1.6)	6.2 (3.1)	4.2 (2.2)
Vitamin C[a]	43.5 (20.9)	46.6 (35.4)	56.8 (25.8)	62.4 (40.6)
Thiamin[a]	0.8 (0.3)	0.7 (0.2)	0.9 (0.3)	0.8 (0.3)
Riboflavin[a]	4.6 (6.3)	0.9 (0.8)	2.6 (2.7)	0.9 (0.3)
Niacin[a]	10.4 (4.0)	10.9 (2.7)	11.3 (4.4)	11.4 (4.3)
Pantothenic acid[a]	2.9 (1.8)	2.2 (0.8)	3.5 (2.0)	2.4 (1.4)
Vitamin B6[a]	0.9 (0.4)	0.8 (0.5)	1.1 (0.5)	0.9 (0.4)
Folate[a]	186.4 (92.0)	199.1 (110.4)	216.4 (93.7)	197.9 (97.9)
Vitamin B12[a]	2.5 (1.6)	1.9 (1.3)	2.6 (1.6)	2.1 (1.6)
Magnesium[a]	161.4 (43.5)	132.9 (41.8)	171.1 (40.4)	141.4 (51.9)
Calcium[a]	411.6 (100.3)	359.8 (138.9)	438.1 (137.0)	421.5 (182.7)
Phosphorus[a]	630.8 (95.3)	575.2 (127.3)	642.9 (105.9)	596.7 (133.3)
Potassium[a]	1268.2 (309.1)	1240.7 (390.3)	1394.0 (308.5)	1397.9 (464.7)
Sodium[a]	2161.4 (353.4)	2534.3 (623.3)	2468.9 (411.9)	2902.1 (521.3)
Iron[a]	8.4 (3.5)	8.6 (2.9)	9.5 (4.0)	9.2 (3.5)
Zinc[a]	5.2 (2.8)	4.4 (1.8)	5.9 (3.3)	4.4 (2.3)
Copper[a]	0.7 (0.3)	0.6 (0.2)	0.7 (0.2)	0.6 (0.2)

Manganese[a]	1.6 (0.6)	1.5 (0.6)	1.8 (0.5)	1.5 (0.6)
Selenium[a]	34.8 (15.2)	32.5 (15.8)	34.6 (16.5)	29.8 (13.5)

FFQ=food frequency questionnaire; DR=dietary recall; RAE= Retinol Activity Equivalent
[a] intakes were adjusted using the nutrient density method (intake/total energy intake*1000). Energy intake and macronutrients expressed in % total energy intake were not adjusted.

Table S5 Indirect validity: mean (SD) daily nutrient intake as assessed by 24-hour DRs of six days according to tertiles of food group consumption (FFQ)

		Tertiles of food group consumption frequency according to FFQ			p-value*
		First	Second	Third	
Cereals					
	Energy Intake (kcal)	**1482.9 (381.0)**	**1736.1 (485.0)**	**1988.6 (549.3)**	<0.001
	CHO (g)	**174.6 (51.6)**	**212.1 (60.2)**	**246.2 (73.8)**	<0.001
	Vitamin A (μg)	**252.7 (176.6)**	240.2 (189.3)	**348.0 (416.7)**[†]	0.034
	Thiamin (mg)	**1.2 (0.5)**	1.3 (0.5)	**1.5 (0.6)**[†]	0.008
	Iron (mg)	**14.1 (6.1)**	15.1 (6.8)	**16.9 (6.7)**[†]	0.028
	Calcium (mg)	**597.8 (250.9)**	**653.9 (329.9)**	**797.2 (393.6)**[†,‡]	0.001
	Copper (mg)	**1.0 (0.4)**	**1.0 (0.4)**	**1.1 (0.5)**[†,‡]	0.017
	Selenium (μg)	**48.0 (25.5)**	50.3 (27.3)	**60.8 (33.0)**[†]	0.013
	Manganese (mg)	**2.3 (0.9)**	2.5 (1.2)	**2.9 (1.3)**[†]	0.011
	Phosphorus (mg)	**896.5 (235.1)**	1010.1 (374.3)	**1135.8 (393.7)**[†]	0.000
	Magnesium (mg)	**220.7 (86.0)**	228.7 (86.3)	**259.1 (111.0)**[†]	0.03
	Zinc (mg)	**6.6 (3.4)**	7.5 (4.0)	**8.5 (4.7)**[†]	0.02
	Sodium (mg)	**4238.9 (639.8)**	**4589.0 (854.6)**[†]	**4854.5 (889.5)**[†]	0.000
Refined cereals					
	Energy Intake (kcal)	**1535.1 (423.1)**	**1759.4 (501.6)**[†]	**1916.1 (554.0)**[†]	0.000

	CHO (%)	**47.5 (7.1)**	48.7 (7.4)	**50.3 (6.3)**†	0.048
	CHO (g)	**181.2 (58.1)**	**213.0 (64.2)**	**239.2 (71.6)**	0.000
	Sodium (mg)	**4339.7 (722.9)**	4549.0 (810.7)	**4798.7 (917.0)**†	0.002
Whole Grain cereals					
	Fibers (g)	**16.7 (9.6)**	**17.2 (6.4)**	**21.7 (8.8)**†,‡	<0.001
	Vitamin E (mg)	**6.1 (3.5)**	**6.8 (3.6)**	**8.2 (3.9)**†,‡	0.002
	Folate (μg)	**295.7 (200.5)**	**318.4 (161.7)**	**402.5 (216.6)**†,‡	0.002
	Thiamin (mg)	**1.2 (0.5)**	**1.3 (0.5)**	**1.5 (0.5)**†,‡	0.005
	B5 (mg)	**3.6 (2.2)**	3.7 (1.9)	**4.5 (2.4)**†	0.02
	B6 (mg)	**1.3 (0.8)**	1.4 (0.8)	**1.7 (0.9)**†	0.014
	Zinc (mg)	7.5 (5.0)	**6.7 (3.1)**	**8.4 (3.9)**‡	0.03
	Potassium (mg)	**2116.1 (955.8)**	2236.5 (741.8)	**2487.6 (802.5)**†	0.019
	Magnesium (mg)	**212.8 (98.6)**	231.5 (91.1)	**264.3 (92.7)**†	0.003
	Copper (mg)	**0.9 (0.4)**	**1.0 (0.4)**	**1.2 (0.4)**†,‡	<0.001
	Selenium (μg)	**47.2 (24.5)**	50.9 (30.0)	**61.0 (31.1)**†	0.009
	Manganese (mg)	**2.3 (1.0)**	**2.4 (0.9)**	**3.0 (1.3)**†,‡	<0.001
	Phosphorus (mg)	978.7 (388.6)	**963.5 (294.3)**	**1102.2 (362.8)**‡	0.025
Legumes					
	Energy Intake (kcal)	**1646.8 (458.3)**	1710.3 (485.1)	**1852.3 (585.3)**†	0.037

	Protein (g)	**61.3 (18.8)**	**63.8 (20.0)**	**73.0 (27.2)**†,‡	0.003
	Lipids (g)	65.7 (24.3)	65.3 (24.4)	74.3 (26.6)	0.04
	SFA (%)	**8.5 (2.4)**	7.8 (2.4)	**7.6 (2.4)**†	0.047
	PUFA (g)	**10.0 (4.4)**	**9.7 (3.6)**	**12.2 (7.0)**†,‡	0.004
	Fibers (g)	**16.1 (7.5)**	18.9 (8.2)	**20.5 (9.5)**†	0.004
	Folate (μg)	**286.6 (170.5)**	337.7 (164.9)	**391.3 (239.6)**†	0.004
	Thiamin	**1.2 (0.5)**	1.3 (0.5)	**1.5 (0.6)**†	0.008
	Niacin	**18.0 (7.6)**	18.4 (6.5)	**20.9 (8.2)**†	0.029
	B5	**3.4 (1.2)**	4.0 (2.3)	**4.3 (2.7)**†	0.032
	Iron (mg)	**13.7 (5.8)**	15.6 (6.6)	**16.8 (7.1)**†	0.012
	Phosphorus (mg)	**940.7 (302.3)**	1008.5 (331.8)	**1093.5 (408.1)**†	0.024
	Magnesium (mg)	**219.3 (92.3)**	230.4 (74.3)	**258.6 (114.2)**†	0.028
	Copper (mg)	**0.9 (0.4)**	**1.0 (0.4)**	**1.1 (0.5)**†,‡	0.002
	Manganese (mg)	**2.3 (0.9)**	2.5 (1.0)	**2.9 (1.4)**†	0.004
	Sodium (mg)	**4414.7 (712.0)**	**4477.2 (660.6)**	**4792.4 (1046.5)**†,‡	0.009
	Selenium	**48.4 (24.6)**	**47.0 (21.2)**	**63.4 (36.7)**†,‡	0.000
Fruits					
	CHO (%)	**23.9 (9.5)**	**23.4 (8.7)**	**27.8 (9.0)**†,‡	0.004
	Fibers (g)	**16.4 (7.7)**	19.1 (9.7)	**20.1 (8.1)**†	0.018
	Vitamin C (mg)	**76.8 (53.5)**	**90.3 (59.6)**	**118.8 (79.3)**†,‡	<0.001

Vegetables					
	Fibers (g)	**16.5 (8.5)**	18.7 (9.3)	**20.3 (7.8)**†	0.023
	Vitamin C (mg)	**79.2 (59.8)**	**89.7 (57.7)**	**116.4 (77.4)**†,‡	0.001
Total Green leafy vegetables					
	Vitamin C	**71.6 (51.6)**	**100.6 (63.4)**†	**112.6 (77.4)**†	<0.001
	Vitamin E	**6.0 (3.0)**	7.1 (3.9)	**7.9 (4.0)**†	0.005
	Fibers	**16.7 (9.4)**	18.4 (9.0)	**20.4 (7.1)**†	0.024
Cooked green leafy vegetables					
	Folate (μg)	**294.9 (138.5)**	**320.2 (206.5)**	**393.7 (224.1)**†,‡	0.004
	Thiamine (mg)	**1.2 (0.5)**	1.3 (0.6)	**0.5 (0.5)**†	0.003
	B5 (mg)	**3.5 (1.5)**	3.9 (2.2)	**4.4 (2.6)**†	0.03
	B6 (mg)	**1.3 (0.6)**	1.4 (0.8)	**1.7 (1.0)**†	0.014
	Vitamin C	**73.8 (54.7)**	**92.2 (60.0)**	**116.3 (76.6)**†,‡	0.000
	Vitamin E	**6.2 (3.1)**	6.8 (3.5)	**8.0 (4.2)**†	0.008
	Iron (mg)	**13.9 (6.1)**	15.2 (6.5)	**16.7 (7.0)**†	0.031
	Calcium (mg)	**584.3 (253.4)**	707.9 (383.9)	**747.0 (346.8)**†	0.007
	Copper (mg)	1.0 (0.4)	0.9 (0.4)	1.1 (0.5)	0.036
	Manganese (mg)	2.5 (1.1)	2.4 (1.1)	2.8 (1.2)	0.044

Raw Green leafy vegetables					
	Vitamin C	**71.6 (51.6)**	**100.6 (63.1)†**	**112.6 (77.4)†**	0.001
	Thiamine	1.3 (0.5)	**1.4 (0.6)**	**1.4 (0.5)‡**	0.037
	Niacin	**19.4 (7.6)**	**19.4 (7.5)**	**18.6 (7.7)†‡**	0.009
	Potassium	**2096.8 (913.9)**	**2430.2 (876.4)†**	2260.1 (727.3)	0.048
Red meat					
	Energy Intake (kcal)	**1573.4 (500.4)**	**1792.4 (444.6)†**	**1838.9 (570.6)†**	0.003
	Protein (g)	**58.1 (19.1)**	**66.8 (20.7)†**	**73.1 (25.8)†**	<0.001
	Lipids (g)	**62.1 (24.0)**	67.8 (22.6)	**75.4 (27.7)†**	0.004
	Cholesterol (mg)	117.8 (82.4)	120.1 (79.5)	150.7 (114.0)	0.048
	Riboflavin	**1.4 (0.7)**	1.5 (0.6)	**1.9 (2.1)†**	0.024
	Niacin	**17.5 (7.4)**	19.3 (6.9)	**20.6 (8.1)†**	0.037
	Phosphorus (mg)	**908.9 (314.4)**	**1050.2 (330.8)†**	**1080.6 (394.2)†**	0.005
	Selenium (μg)	**46.7 (26.6)**	52.3 (27.8)	**59.8 (31.7)†**	0.018
	Calcium	**618.5 (310.1)**	675.1 (308.4)	**754.3 (385.1)†**	0..042
Chicken					
	Energy Intake (kcal)	**1561.7 (470.6)**	1750.6 (468.7)	**1874.5 (565.1)†**	0.001
	Protein (%)	**14.5 (2.7)**	15.4 (3.0)	**15.7 (2.8)†**	0.03
	Protein (g)	**56.4 (17.2)**	**66.1 (189)**	**74.3 (27.3)**	<0.001

	Lipids (g)	**59.5 (23.7)**	68.8 (23.0)	**75.8 (26.8)†**	<0.001
	SFA (g)	13.4 (6.0)	**16.9 (10.0)†**	**17.0 (6.6)†**	0.007
	Cholesterol (mg)	**107.6 (79.0)**	**120.8 (77.1)**	**157.4 (114.1)†,‡**	0.003
	Niacin (mg)	**17.5 (6.8)**	18.9 (7.3)	**20.8 (8.2)†**	0.025
	Iron (mg)	**13.9 (5.5)**	15.3 (7.5)	**16.7 (6.4)†**	0.032
	Phosphorus (mg)	**894.1 (287.5)**	**1040.0 (333.6)†**	**1092.5 (402.0)†**	0.002
	Calcium	**575.6 (244.2)**	695.8 (344.0)	**763.0 (382.2)†**	0.003
	Sodium	**4291.9 (621.7)**	**4628.4 (796.8)†**	**4728.1 (984.0)†**	0.004
Fish					
	Energy Intake (kcal)	**1668.8 (481.3)**	**1743.9 (499.3)**	**2003.9 (645.2)†,‡**	0.008
	Protein (g)	**61.5 (19.1)**	68.0 (24.1)	**79.0 (27.8)†**	0.001
	MUFA (g)	**23.1 (11.1)**	24.1 (9.6)	**28.5 (11.5)†**	0.055
	Cholesterol (mg)	**122.6 (68.7)**	**134.9 (89.7)**	**183.5 (160.5)†,‡**	0.001
	Vitamin A (µg)	**249.0 (250.8)**	284.7 (216.7)	**397.2 (516.8)†**	0.046
	Vitamin E (mg)	**6.3 (3.4)**	7.5 (3.8)	**8.5 (4.2)†**	0.006
	Folate (µg)	**293.2 (155.3)**	**366.8 (190.5)†**	**441.4 (312.4)†**	<0.001
	Vitamin D (µg)	**8.0 (1.1)**	8.4 (1.4)	**9.3 (3.0)†**	<0.001
	Vitamin B12 (µg)	**2.9 (2.1)**	**4.1 (3.0)†**	4.0 (3.3)	0.004
	Thiamin	**1.3 (0.5)**	1.4 (0.5)	**1.5 (0.7)†**	0.014

	Niacin	**17.9 (7.0)**	19.7 (7.3)	**22.3 (9.7)†**	0.015
	B5	**3.5 (1.9)**	4.1 (2.1)	**5.0 (3.1)†**	0.006
	B6	**1.3 (0.7)**	**1.6 (0.9)†**	**1.8 (1.1)†**	0.000
	Iron (mg)	**13.8 (5.1)**	**16.8 (7.3)†**	**17.3 (8.3)†**	0.001
	Calcium (mg)	**632.7 (289.8)**	**693.1 (345.1)**	**864.8 (448.9)†,‡**	0.004
	Phosphorus (mg)	**943.8 (324.3)**	**1042.6 (344.3)**	**1224.1 (422.6)†,‡**	<0.001
	Zinc (mg)	**6.9 (3.5)**	7.7 (3.9)	**9.4 (6.3)†**	0.015
	Potassium (mg)	**2127.1 (851.0)**	**2321.5 (764.0)**	**2793.8 (909.1)†,‡**	0.001
	Copper (mg)	**0.9 (0.4)**	**1.0 (0.4)**	**1.3 (0.5)†,‡**	0.001
	Selenium (μg)	**46.8 (23.6)**	56.0 (27.0)	**69.7 (46.1)†**	<0.001
	Manganese (mg)	2.4 (1.1)	2.7 (1.1)	3.0 (1.4)	0.024
	Magnesium	**222.1 (93.2)**	**236.6 (80.9)**	**294.9 (130.2)†,‡**	0.001
Shellfish					
	Energy Intake (kcal)	**1637.4 (497.9)**	1719.7 (513.5)	**1847.1 (526.0)†**	0.039
	SFA (g)	**14.1 (6.1)**	15.3 (5.8)	**18.1 (10.6)†**	0.005
	Vitamin A (μg)	**196.7 (207.0)**	280.9 (238.4)	**356.4 (365.2)†**	0.002
	Vitamin E (mg)	**6.0 (3.2)**	7.3 (4.3)	**7.7 (3.4)†**	0.018
	Vitamin B12 (μg)	**2.6 (1.9)**	3.5 (2.3)	**4.3 (3.3)†**	<0.001
	B6	**1.2 (0.7)**	1.5 (0.7)	**1.7 (1.0)†**	0.01

	Phosphorus (mg)	**928.0 (331.4)**	1028.6 (349.2)	**1079.9 (369.0)**†	0.026
	Zinc (mg)	**6.5 (2.9)**	7.9 (4.4)	**8.1 (4.6)**†	0.032
	Potassium (mg)	**2089.9 (841.7)**	2270.0 (894.8)	**2465.3 (771.6)**†	0.022
	Copper (mg)	**0.9 (0.4)**	1.0 (0.4)	**1.1 (0.4)**†	0.032
Fish and shellfish					
	Energy Intake (kcal)	**1620.1 (447.9)**	**1696.6 (484.0)**	**1894.6 (580.0)**†,‡	0.002
	Protein (%)	**14.5 (2.8)**	**15.8 (2.7)**†	15.5 (3.0)	0.012
	Protein (g)	**58.3 (16.7)**	**66.7 (22.9)**†	**73.2 (25.7)**†	<0.001
	Lipids (g)	**64.6 (23.2)**	66.1 (24.7)	**74.7 (27.1)**†	0.025
	SFA (g)	**14.4 (6.0)**	**14.7 (6.2)**	**18.5 (10.4)**†,‡	0.001
	MUFA (g)	23.1 (11.1)	**22.3 (9.9)**	**26.8 (10.7)**‡	0.017
	PUFA (%)	**5.3 (1.5)**	5.1 (1.7)	**6.0 (2.2)**†	0.006
	PUFA (g)	**9.6 (4.0)**	**9.8 (4.0)**	**12.5 (6.4)**†,‡	<0.001
	Cholesterol (mg)	**112.3 (76.5)**	123.4 (78.2)	**152.8 (118.1)**†	0.019
	Vitamin A (μg)	**234.7 (215.4)**	**246.1 (216.6)**	**360.0 (380.2)**†,‡	0.009
	Vitamin D	**8.0 (1.3)**	**8.1 (1.0)**	**8.7 (2.2)**†,‡	0.009
	Vitamin E (mg)	**6.3 (3.5)**	**6.6 (3.6)**	**8.2 (3.9)**†,‡	0.003
	Folate (μg)	**286.5 (137.5)**	**323.7 (178.1)**	**406.3 (247.6)**†,‡	<0.001
	Vitamin B12 (μg)	**2.8 (2.2)**	3.5 (2.3)	**4.2 (3.2)**†	0.003

	Thiamin	**1.3 (0.5)**	1.3 (0.5)	**1.5 (0.6)**†	0.03
	Riboflavin	**1.4 (0.6)**	1.5 (0.6)	**1.9 (2.1)**†	0.026
	Niacin	**17.4 (7.0)**	18.6 (7.4)	**21.4 (7.8)**†	0.003
	B6	**1.3 (0.6)**	**1.4 (0.7)**	**1.7 (1.0)**†,‡	0.001
	Iron (mg)	**13.7 (5.2)**	15.2 (6.6)	**17.2 (7.5)**†	0.003
	Phosphorus (mg)	**902.1 (299.4)**	1014.4 (336.3)	**1127.2 (390.2)**†	<0.001
	Zinc (mg)	**6.6 (3.5)**	7.4 (3.7)	**8.6 (4.9)**†	0.008
	Potassium (mg)	**2028.2 (745.0)**	**2197.5 (1.828.1)**	**2615.0 (867.8)**†,‡	<0.001
	Copper (mg)	**0.9 (0.4)**	**1.0 (0.4)**	**1.2 (0.5)**†,‡	<0.001
	Selenium (μg)	**43.3 (21.3)**	53.7 (29.7)	**62.0 (32.6)**†	<0.001
	Manganese (mg)	**2.3 (0.9)**	2.6 (1.1)	**2.9 (1.3)**†	0.003
	Magnesium (mg)	**208.7 (77.9)**	**230.2 (90.3)**	**269.8 (108.7)**†,‡	0.000
Chicken and red meat					
	Energy Intake (kcal)	**1568.0 (439.6)**	1742.6 (479.6)	**1900.2 (576.9)**†	<0.001
	Protein (g)	**57.1 (17.1)**	**65.9 (19.8)**	**75.2 (26.9)**	<0.001
	Lipids (g)	**58.7 (21.1)**	**69.6 (23.4)**†	**77.0 (27.9)**†	<0.001
	Cholesterol (mg)	**111.1 (72.8)**	**119.3 (81.4)**	**158.2 (116.5)**†,‡	0.003
	Thiamin	**1.3 (0.5)**	**1.3 (0.5)**	**1.5 (0.6)**†,‡	0.009
	Riboflavin	**1.4 (0.5)**	**1.5 (0.7)**	**2.0 (2.1)**†,‡	0.007

	Niacin	**17.3 (6.6)**	**18.3 (6.9)**	**21.7 (8.4)**†,‡	0.000
	B5	**3.6 (1.5)**	3.7 (1.5)	**4.5 (3.1)**†	0.021
	Iron (mg)	**14.2 (5.5)**	**14.6 (7.2)**	**17.2 (6.8)**†,‡	0.008
	Phosphorus (mg)	**910.7 (297.6)**	1031.8 (340.5)	**1100.9 (396.6)**†	0.003
	Zinc (mg)	**6.5 (2.5)**	7.6 (4.4)	**8.5 (4.8)**†	0.012
	Selenium (µg)	**47.0 (25.8)**	**49.7 (23.5)**	**62.4 (35.0)**†,‡	0.002
	Ca	**611.6 (266.2)**	685.0 (347.1)	**752.6 (383.5)**†	0.032
	Na	**4320.7 (621.0)**	**4655.3 (765.8)**†	**4710.1 (1032.7)**†	0.006
Organ meats					
	Lipids (g)	**63.6 (24.0)**	69.2 (23.6)	**73.2 (27.4)**†	0.044
Eggs					
	Energy Intake (kcal)	**1585.7 (399.2)**	1727.9 (509.2)	**1879.3 (582.6)**†	0.002
	Protein (g)	**57.8 (16.9)**	**66.2 (21.9)**†	**73.1 (26.0)**†	<0.001
	Lipids (g)	**63.8 (22.4)**	67.2 (25.7)	**73.9 (26.6)**†	0.043
	PUFA (g)	**9.7 (4.1)**	10.2 (4.7)	**12.0 (6.5)**†	0.017
	Cholesterol (mg)	**100.5 (51.5)**	**122.7 (68.9)**	**162.6 (131.2)**†,‡	<0.001
	Folate (µg)	**281.7 (114.8)**	341.4 (187.6)	**385.6 (251.5)**†	0.006
	Thiamin	**1.2 (0.4)**	1.4 (0.5)	**1.5 (0.6)**†	0.009
	Riboflavin	**1.3 (0.5)**	1.6 (1.1)	**1.9 (1.9)**†	0.042

	Niacin	**18.0 (7.4)**	18.4 (6.9)	**20.9 (8.2)†**	0.032
	B5	**3.4 (1.4)**	3.9 (2.1)	**4.4 (2.7)†**	0.016
	Iron (mg)	**13.1 (4.6)**	**16.2 (6.9)†**	**16.4 (7.4)†**	0.003
	Phosphorus (mg)	**907.8 (288.4)**	1017.1 (345.2)	**1105.0 (394.0)†**	0.003
	Zinc (mg)	**6.5 (2.8)**	7.7 (3.7)	**8.2 (5.2)†**	0.04
	Potassium (mg)	**2071.5 (779.1)**	2324.3 (863.9)	**2411.3 (865.9)†**	0.042
	Selenium (μg)	**47.6 (24.0)**	**49.3 (22.7)**	**62.0 (37.0)†,‡**	0.003
	Ca	**598.8 (260.4)**	702.6 (355.6)	**734.5 (370.5)†**	0.041
	Na	**4408.6 (715.3)**	4517.5 (843.2)	**4747.8 (906.0)†**	0.039
	Cu	**0.9 (0.3)**	1.0 (0.4)	**1.1 (0.5)†**	0.011
Dairy products					
	Protein (%)	**14.6 (2.9)**	**16.0 (3.0)†**	15.2 (2.5)	0.009
	Protein (g)	**59.3 (17.6)**	**72.3 (25.8)†**	66.4 (22.6)	0.001
	Lipids (g)	**62.6 (22.8)**	**73.3 (26.7)†**	69.4 (25.5)	0.025
	SFA (g)	**13.8 (5.5)**	**17.3 (8.4)†**	16.5 (9.2)	0.014
	Cholesterol (mg)	**109.0 (65.0)**	**149.5 (98.0)†**	129.7 (109.9)	0.024
	Vitamin A (μg)	**208.5 (161.5)**	283.1 (288.1)	**348.6 (357.9)†**	0.008
	Calcium (mg)	**573.5 (284.0)**	680.0 (299.3)	**795.8 (392.0)†**	<0.001
	Phosphorus (mg)	**903.6 (280.9)**	**1072.1 (384.8)†**	**1067.2 (366.8)†**	0.003

	Selenium (µg)	**46.3 (22.3)**	**57.4 (32.4)**†	55.2 (30.9)	0.038
Full-fat dairy products					
	Protein (g)	**61.8 (20.6)**	**71.6 (25.8)**†	64.6 (20.7)	0.02
	PUFA (%)	**5.8 (2.3)**	5.6 (1.6)	**5.0 (1.6)**†	0.036
	Sodium (mg)	**4404.4 (796.3)**	**4777.1 (920.1)**†	4502.4 (754.0)	0.014
Low-fat dairy products					
	Protein (%)	**15.0 (2.8)**	**14.7 (2.8)**	**16.1 (2.9)**†,‡	0.003
	Protein (g)	64.0 (20.9)	**62.8 (21.7)**	**71.4 (25.0)**‡	0.035
	Vitamin A (µg)	**200.3 (142.2)**	272.5 (283.7)	**367.6 (363.6)**†	0.001
	Vitamin B12 (µg)	**3.0 (2.3)**	3.5 (3.2)	**4.0 (2.3)**†	0.05
	Riboflavin	1.5 (1.1)	1.5 (0.7)	1.9 (1.9)	0.042
	B5	**3.6 (1.4)**	**3.6 (2.1)**	**4.6 (2.8)**†,‡	0.001
	B6	**1.3 (0.6)**	1.4 (0.9)	**1.6 (0.8)**†	0.029
	Calcium (mg)	**609.4 (294.7)**	**607.6 (270.0)**	**833.2 (394.1)**†,‡	<0.001
	Phosphorus (mg)	**942.3 (310.8)**	**946.2 (314.0)**	**1156.1 (394.1)**†,‡	<0.001
	Zinc (mg)	**7.1 (3.0)**	**6.7 (3.5)**	**8.8 (5.3)**†,‡	0.002
	Potassium (mg)	2207.3 (801.3)	**2118.9 (741.7)**	**2515.5 (948.7)**‡	0.008
	Magnesium (mg)	**223.1 (82.1)**	**216.4 (73.2)**	**269.5 (118.8)**†,‡	0.001
	Copper	**0.9 (0.4)**	**0.9 (0.4)**	**1.1 (0.5)**†,‡	0.003

	Manganese	**2.4 (1)**	2.5 (0.9)	**2.9 (1.3)†**	0.026
	Selenium (µg)	**46.2 (20.8)**	**50.4 (29.0)**	**62.4 (34.1)†,‡**	0.001
Processed meats					
	Energy Intake (kcal)	**1595.0 (453.5)**	1741.2 (483.0)	**1872.8 (577.7)†**	0.003
	Protein (g)	**60.8 (21.3)**	65.9 (21.6)	**71.4 (24.4)†**	0.014
	Lipids (g)	**59.1 (21.4)**	**70.2 (25.4)†**	**75.9 (26.3)†**	<0.001
	SFA (g)	**13.9 (6.4)**	**15.3 (6.1)**	**18.4 (10.2)†,‡**	0.001
	MUFA (g)	**21.1 (9.1)**	**25.3 (11.8)†**	**25.8 (10.6)†**	0.011
	Cholesterol (mg)	120.6 (80.6)	116.7 (71.0)	151.4 (120.6)	0.038
	Sodium (mg)	**4372 (745.3)**	4600.2 (788.0)	**4710.9 (944.1)†**	0.036
Fast food sandwiches					
	Energy Intake (kcal)	**1633.2 (453.0)**	1714.6 (490.6)	**1845.0 (577.8)†**	0.038
	Sodium (mg)	**4299.1 (659.0)**	**4624.4 (795.1)†**	**4713.2 (964.1)†**	0.003
Dessert					
	Energy Intake (kcal)	1676.1 (539.3)	1678.0 (488.2)	1857.5 (511.3)	0.04
	CHO (g)	**199.8 (73.1)**	205.3 (62.9)	**228.3 (67.6)†**	0.021
	Sugar (g)	**46.5 (28.5)**	**51.2 (22.1)**	**61.7 (28.8)†,‡**	0.002
	Lipids (g)	65.3 (26.4)	65.9 (24.0)	74.2 (25.0)	0.049
	SFA (g)	**14.8 (7.8)**	**14.7 (5.8)**	**18.1 (9.5)†,‡**	0.011

	Sodium (mg)	4502.6 (842.1)	**4427.9 (735.5)**	**4759.4 (905.3)**‡	0.032
Fats and oils					
	Energy Intake (kcal)	**1604.3 (493.4)**	1762.8 (498.9)	**1843.5 (538.0)**†	0.012
	SFA (%)	**8.5 (2.4)**	8.1 (2.5)	**7.5 (2.3)**†	0.027
Unsaturated fats and oils					
	Energy Intake (kcal)	1600.1 (484.9)	1796.5 (558.4)	1813.6 (485.2)	0.015
	SFA (%)	8.3 (2.3)	8.3 (2.6)	7.5 (2.3)	0.046
	MUFA (g)	**21.9 (9.3)**	24.1 (9.7)	**26.2 (12.6)**†	0.043
Raw nuts					
	Lipids (%)	34.3 (6.7)	36.7 (5.6)	36.9 (6.4)	0.024
	MUFA (%)	**11.9 (3.1)**	12.9 (4.3)	**13.6 (3.2)**†	0.008
Fried salted nuts					
	Sodium (mg)	**4368.3 (677.3)**	4607.9 (943.7)	**4691.9 (842.9)**†	0.043
	Manganese (mg)	**2.3 (1.0)**	**2.8 (1.1)**†	2.6 (1.2)	0.04
	Potassium	**2060.8 (761.7)**	2365.9 (785.1)	**2394.1 (944.6)**†	0.025
Alcoholic beverages	Alcohol (%)	**0.0 (0.0)**	**0.4 (0.7)**	**1.5 (2.8)**	<0.001
	Alcohol (g)	**0.0 (0.1)**	**1.0 (2.2)**	**4.2 (8.5)**	<0.001

*One-way ANOVA; P<0.05 is significant

†difference is significant as compared to tertile 1

‡difference is significant as compared to tertile 2
