# Supplementary material for: Development and validation of a quantitative food frequency questionnaire to assess dietary intake among Lebanese adults
Source: Nutr J. 2020 Jul 6;19:65. doi: 10.1186/s12937-020-00581-5 (PMC7339409; doi:10.1186/s12937-020-00581-5)
Supplement: Supplementary file 2 — Additional file 2: Figure S1. Bland-Altman plots of difference between nutrients as predicted by the first FFQ and the mean of six 24-h recalls (n = 238); Figure S2. Bland-Altman plots of difference between nutrients as predicted by the first and second FFQs (n = 52) [file 12937_2020_581_MOESM2_ESM.pdf]

Additional file 2

For

Development and validation of a quantitative food frequency questionnaire to assess dietary intake among Lebanese adults

Mireille Harmouche-Karaki[1*], Maya Mahfouz[1], Jawaher Obeyd[1], Pascale Salameh[2], Yara Mahfouz[1], Khalil Helou[1]

[1]Department of Nutrition, Faculty of Pharmacy, Saint Joseph University, Beirut, Lebanon

[2]Clinical and Epidemiological Research Laboratory, Faculty of Pharmacy, Lebanese University, Hadath, Lebanon

Email: Mireille Harmouche-Karaki*: mireille.harmouche@usj.edu.lb; Maya Mahfouz: mzmahfouz5@hotmail.com; Jawaher Obeyd : jawaherobeyd@hotmail.com; Pascale Salameh : psalameh@ul.edu.lb; Yara Mahfouz: yara.mahfouz@hotmail.com; Khalil Helou: khalil.helou@usj.edu.lb

*Correspondence: Mireille Harmouche-Karaki; e-mail: mireille.harmouche@usj.edu.lb; Telephone: +9613593395; Address: Department of Nutrition, Faculty of Pharmacy, Saint Joseph University of Beirut, Lebanon B.P. 11-5076 – Riad el Solh Beirut 1107 2180 – Lebanon

Table of Contents

Content	Page
Figure S1. Bland-Altman plots of difference between nutrients as predicted by the first FFQ and the mean of six 24-hour recalls (n=238)	3 of 35
Figure S2. Bland-Altman plots of difference between nutrients as predicted by the first and second FFQs (n=52)	18 of 35

Figure S1 Bland-Altman plots of difference between **a**, carbohydrates (grams) **b** protein (grams), **c** fat (grams), **d** SFA (% of TEI), **e**, SFA (grams), **f** MUFA (% of TEI) **g** MUFA (grams), **h** PUFA (% of TEI), **i** PUFA (grams), **j** sugars (% of carbohydrates), **k** sugars (grams), **l** alcohol (grams), **m** vitamin A, **n** vitamin E, **o** vitamin B1, **p** vitamin B2, **q** niacin, **r** vitamin B5, **s** vitamin B6, **t** vitamin B9, **u** vitamin B12, **v** magnesium, **w** phosphorus, **x** potassium, **y** zinc, **z** copper, **aa** manganese, **bb** and selenium, as predicted by the first FFQ and the mean of six 24-hour recalls (n=238)

a

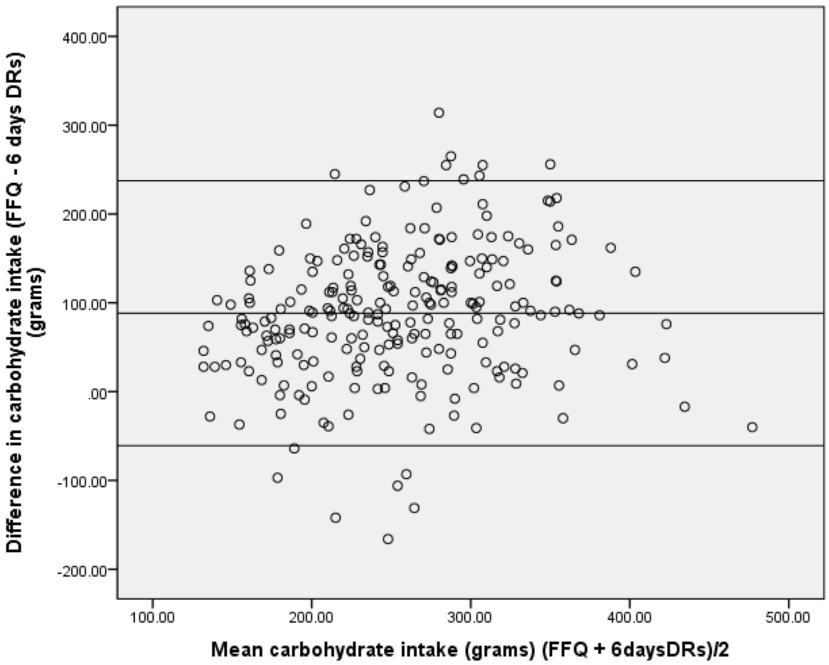

b

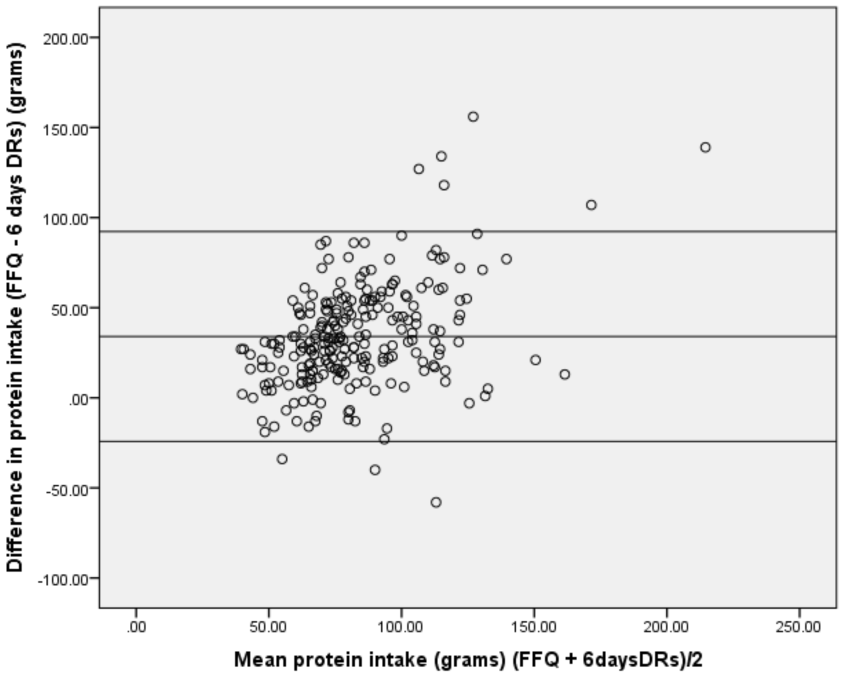
Difference in protein intake (FFQ - 6 days DRs) (grams)
200.00
150.00
100.00
50.00
.00
-50.00
-100.00
.00
50.00
100.00
150.00
200.00
250.00
Mean protein intake (grams) (FFQ + 6daysDRs)/2

c

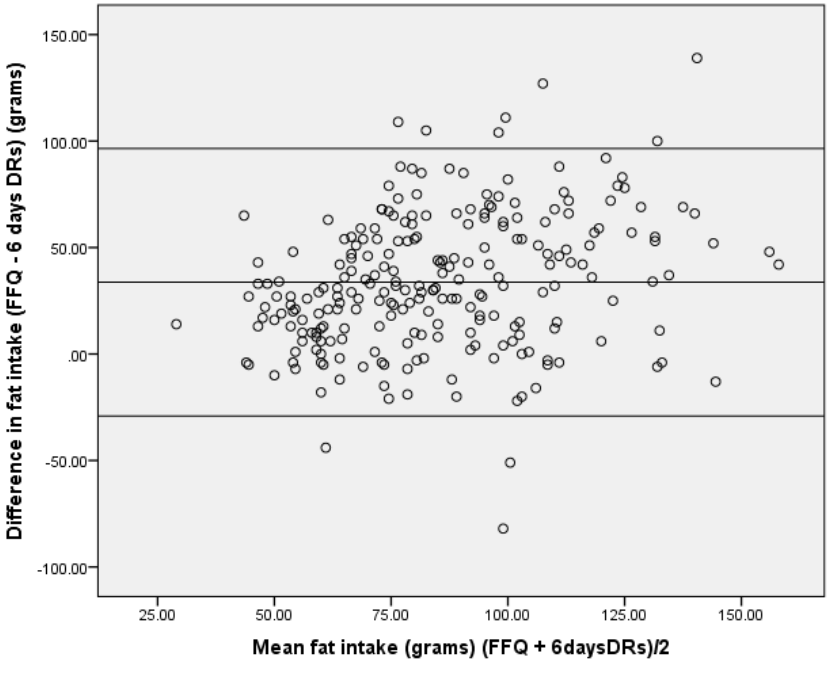
Difference in fat intake (FFQ - 6 days DRs) (grams)
150.00
100.00
50.00
.00
-50.00
-100.00
25.00
50.00
75.00
100.00
125.00
150.00
Mean fat intake (grams) (FFQ + 6daysDRs)/2

d

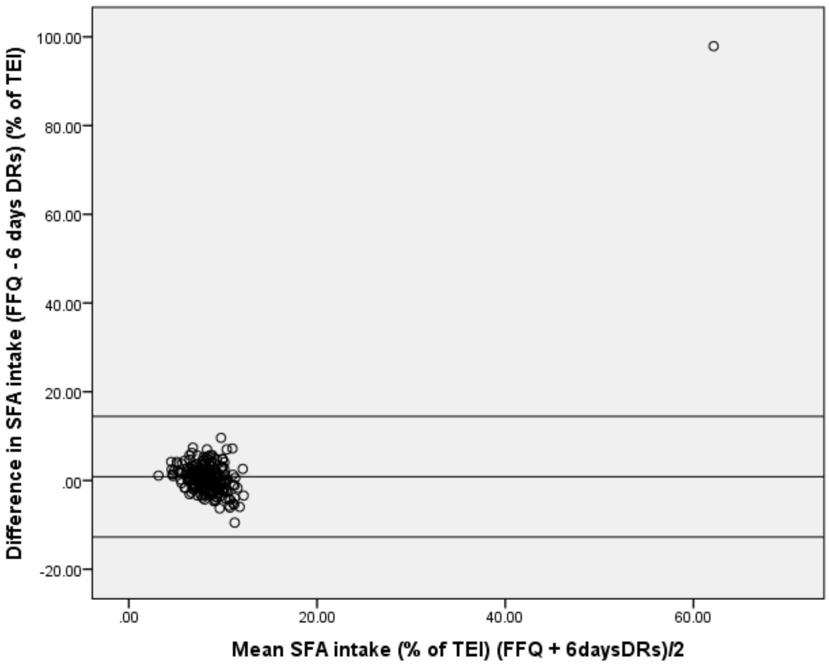
100.00
80.00
60.00
40.00
20.00
.00
-20.00
Difference in SFA intake (FFQ - 6 days DRs) (% of TEI)
.00
20.00
40.00
60.00
Mean SFA intake (% of TEI) (FFQ + 6daysDRs)/2

e

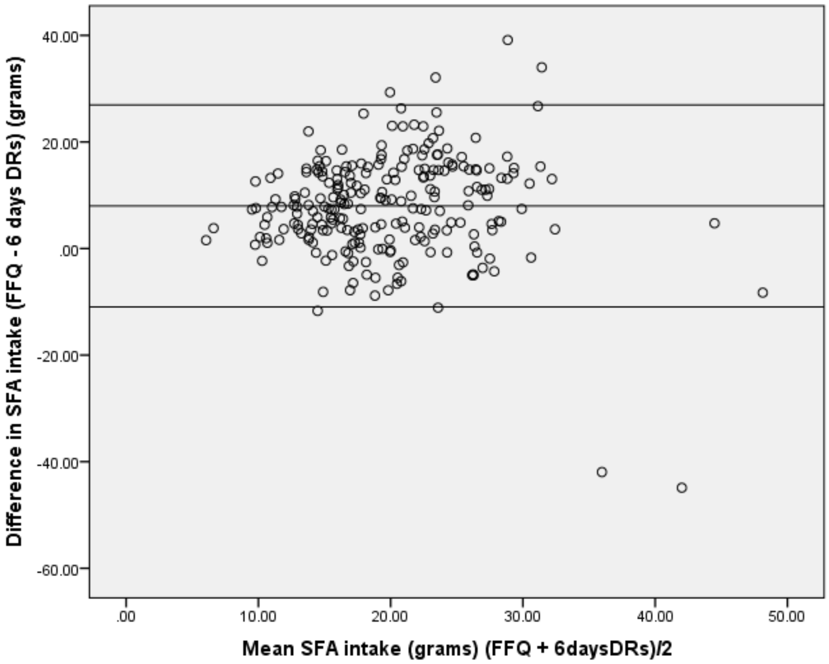
40.00
20.00
.00
-20.00
-40.00
-60.00
Difference in SFA intake (FFQ - 6 days DRs) (grams)
.00
10.00
20.00
30.00
40.00
50.00
Mean SFA intake (grams) (FFQ + 6daysDRs)/2

f

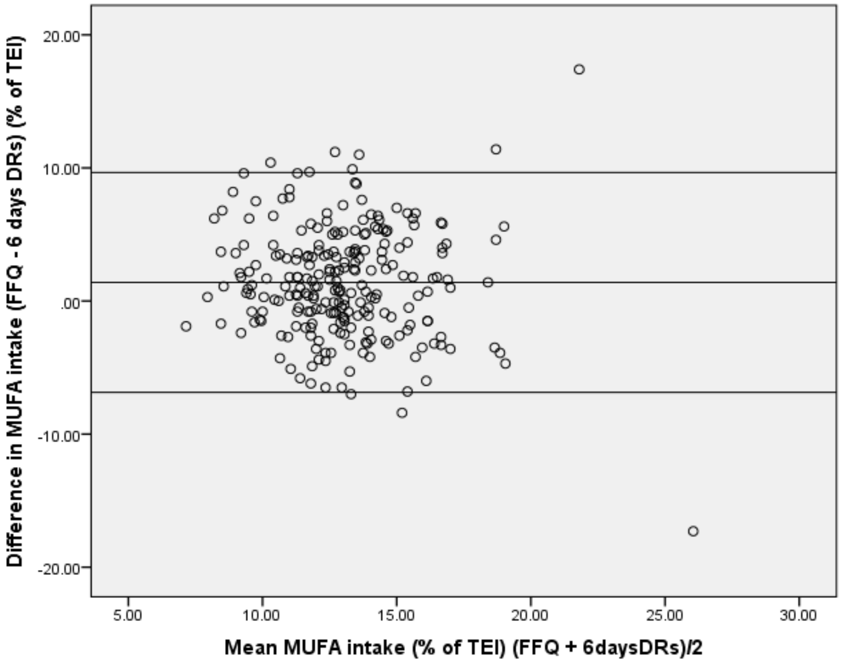
20.00
10.00
.00
-10.00
-20.00
5.00
10.00
15.00
20.00
25.00
30.00
Difference in MUFA intake (FFQ - 6 days DRs) (% of TEI)
Mean MUFA intake (% of TEI) (FFQ + 6daysDRs)/2

g

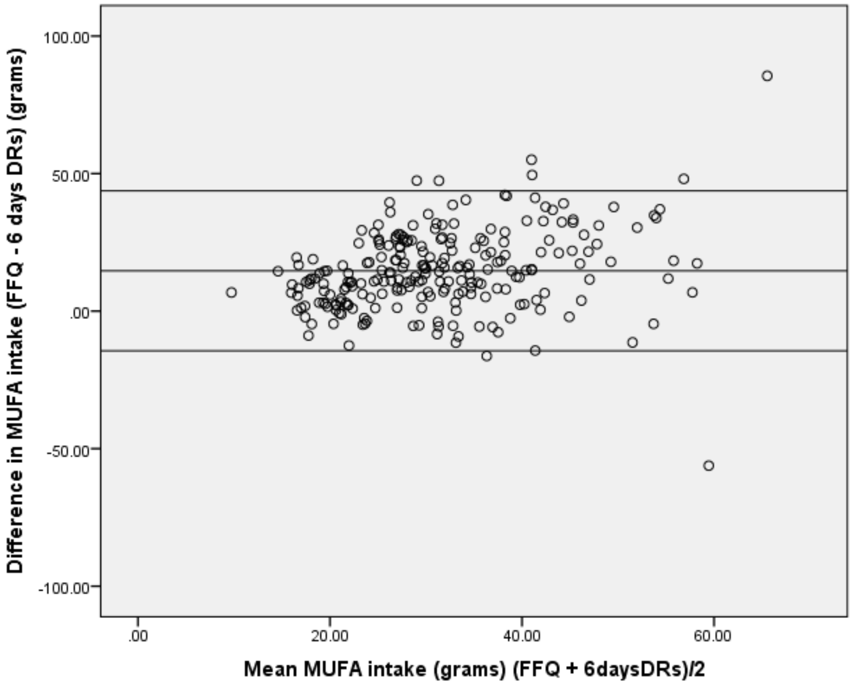
100.00
50.00
.00
-50.00
-100.00
.00
20.00
40.00
60.00
Difference in MUFA intake (FFQ - 6 days DRs) (grams)
Mean MUFA intake (grams) (FFQ + 6daysDRs)/2

h

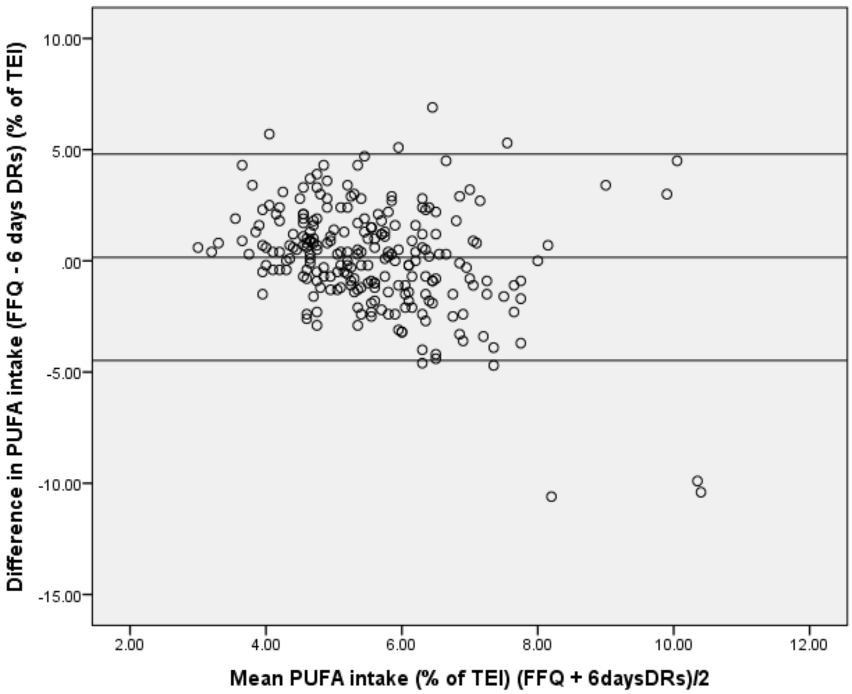
10.00
5.00
.00
-5.00
-10.00
-15.00
Difference in PUFA intake (FFQ - 6 days DRs) (% of TEI)
2.00
4.00
6.00
8.00
10.00
12.00
Mean PUFA intake (% of TEI) (FFQ + 6daysDRs)/2

i

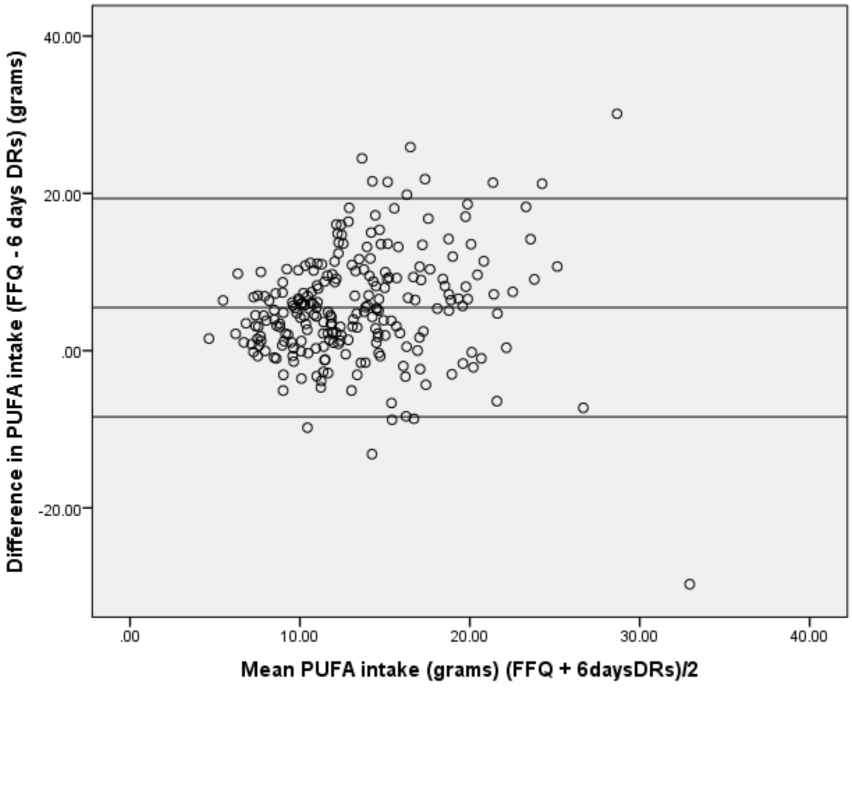
40.00
20.00
.00
-20.00
Difference in PUFA intake (FFQ - 6 days DRs) (grams)
.00
10.00
20.00
30.00
40.00
Mean PUFA intake (grams) (FFQ + 6daysDRs)/2

j

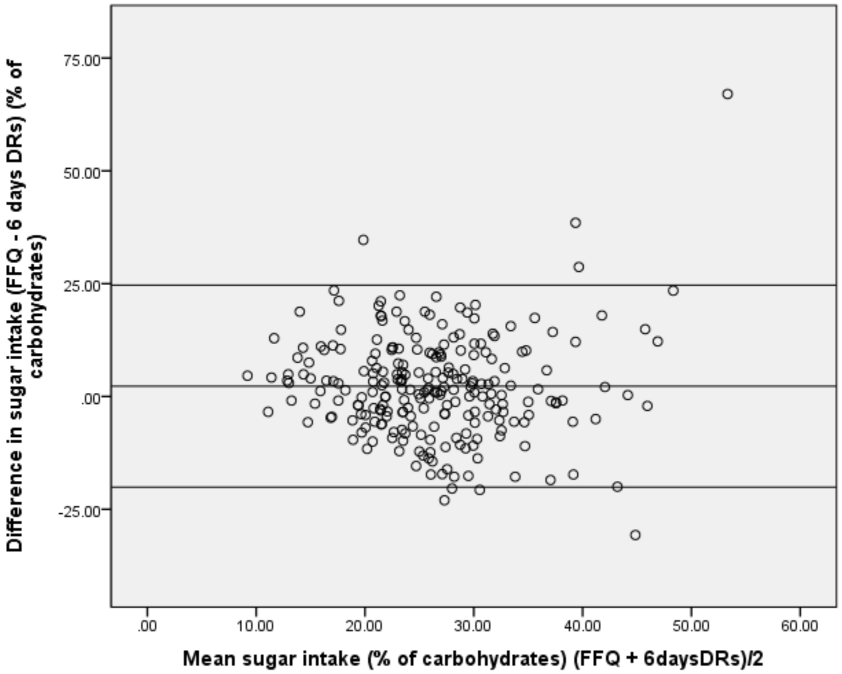
Difference in sugar intake (FFQ - 6 days DRs) (% of carbohydrates)
75.00
50.00
25.00
.00
-25.00
.00
10.00
20.00
30.00
40.00
50.00
60.00
Mean sugar intake (% of carbohydrates) (FFQ + 6daysDRs)/2

k

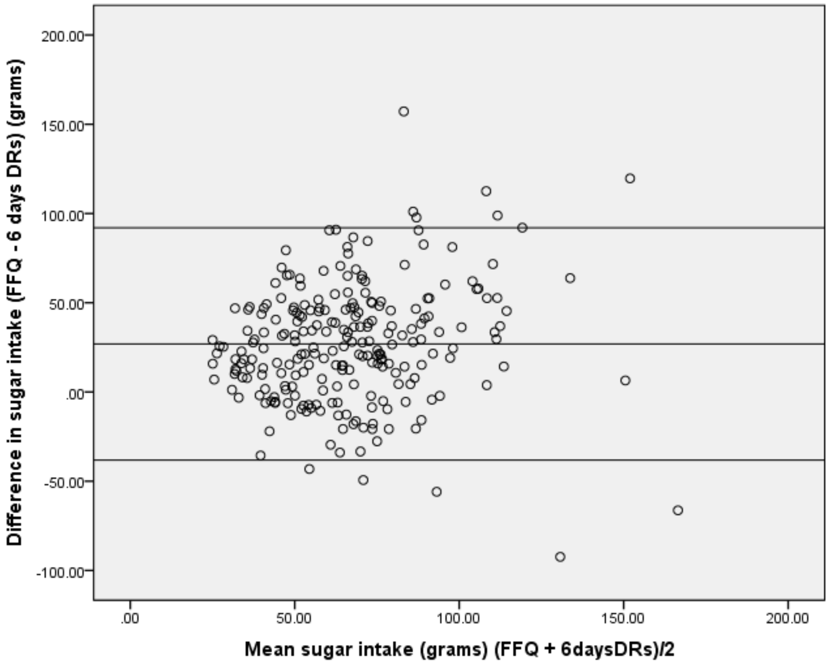
Difference in sugar intake (FFQ - 6 days DRs) (grams)
200.00
150.00
100.00
50.00
.00
-50.00
-100.00
.00
50.00
100.00
150.00
200.00
Mean sugar intake (grams) (FFQ + 6daysDRs)/2

l

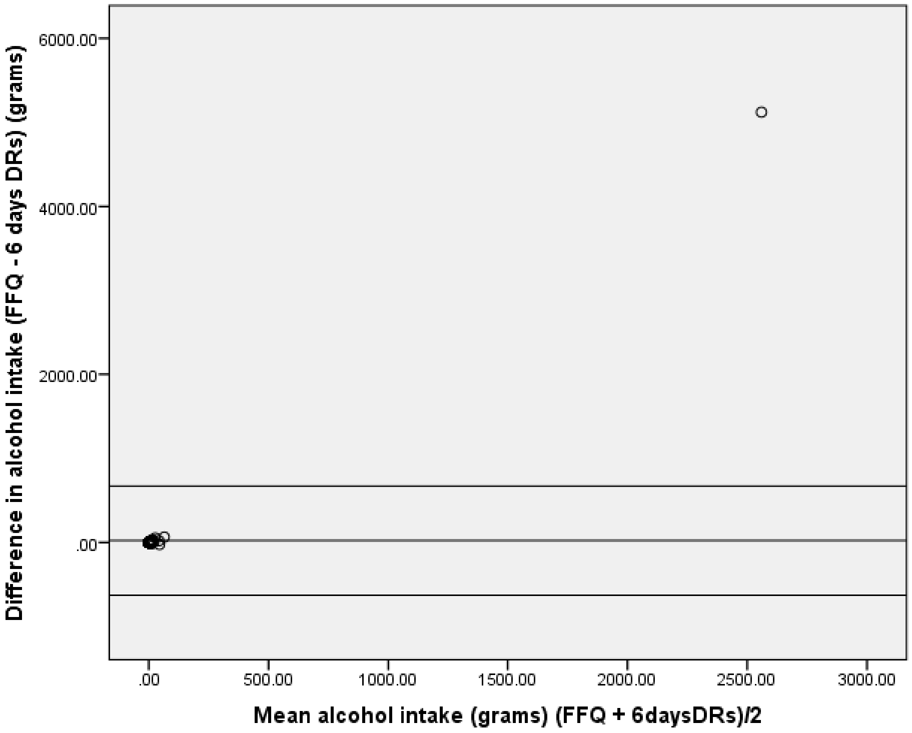
6000.00
4000.00
2000.00
.00
Difference in alcohol intake (FFQ - 6 days DRs) (grams)
.00
500.00
1000.00
1500.00
2000.00
2500.00
3000.00
Mean alcohol intake (grams) (FFQ + 6daysDRs)/2

m

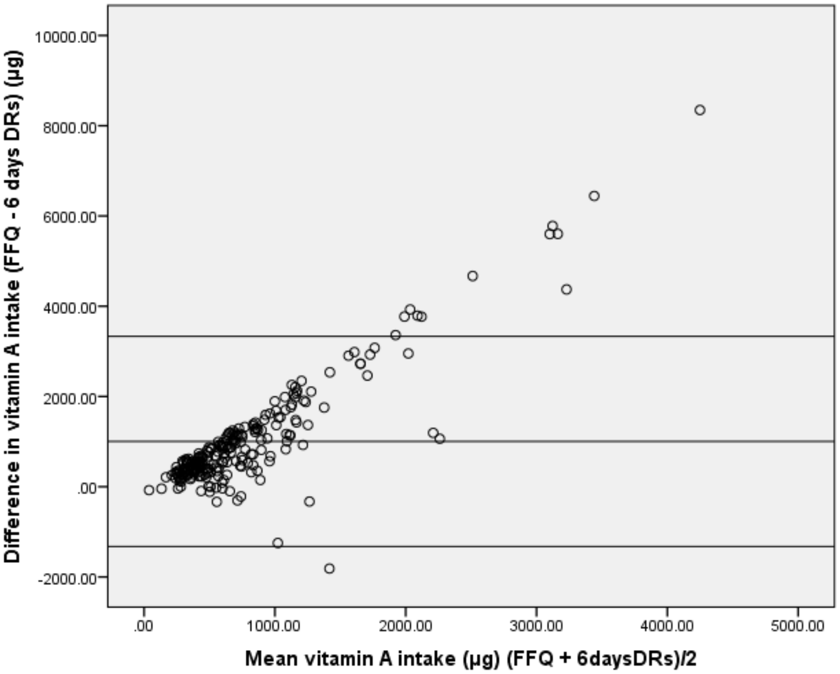
10000.00
8000.00
6000.00
4000.00
2000.00
.00
-2000.00
Difference in vitamin A intake (FFQ - 6 days DRs) (µg)
.00
1000.00
2000.00
3000.00
4000.00
5000.00
Mean vitamin A intake (µg) (FFQ + 6daysDRs)/2

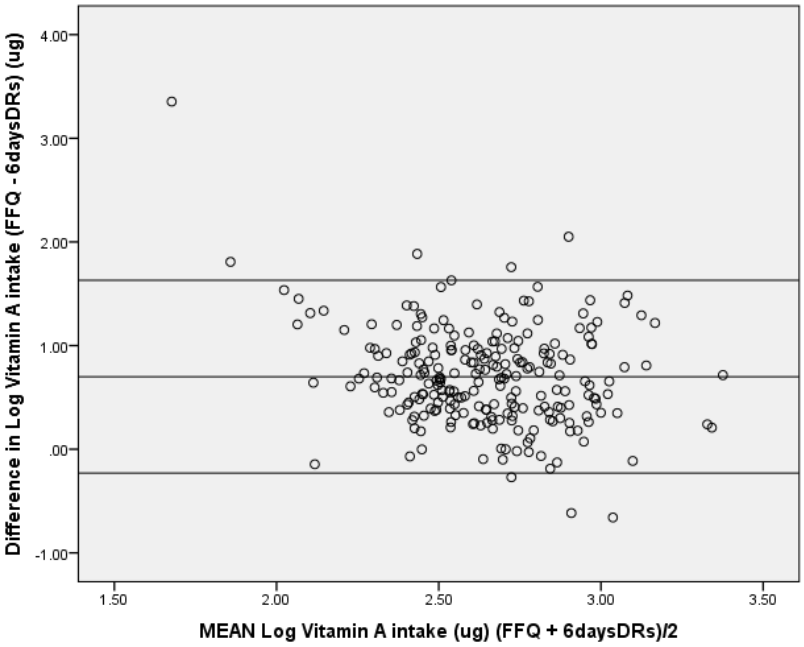
Difference in Log Vitamin A intake (FFQ - 6daysDRs) (ug)
4.00
3.00
2.00
1.00
.00
-1.00
1.50
2.00
2.50
3.00
3.50
MEAN Log Vitamin A intake (ug) (FFQ + 6daysDRs)/2

n

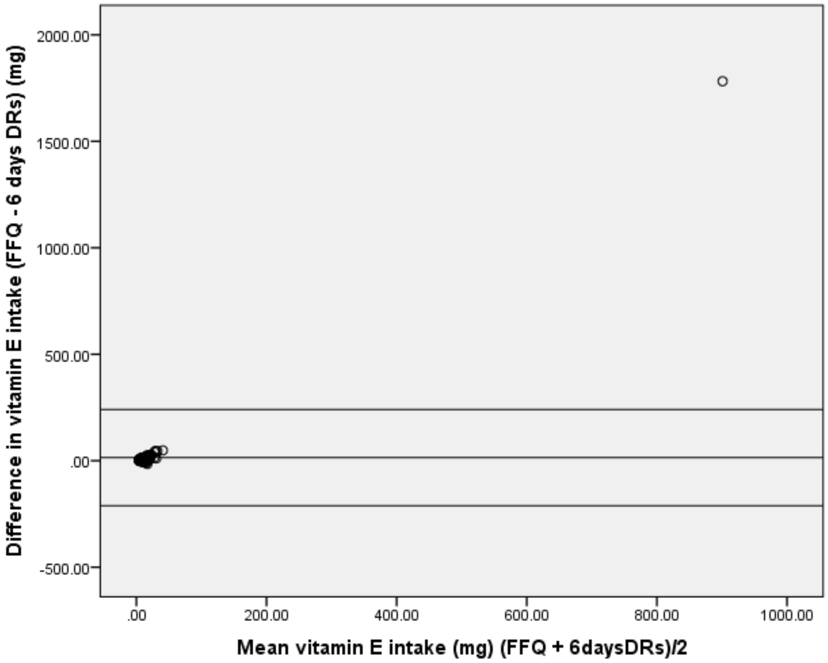
Difference in vitamin E intake (FFQ - 6 days DRs) (mg)
2000.00
1500.00
1000.00
500.00
.00
-500.00
.00
200.00
400.00
600.00
800.00
1000.00
Mean vitamin E intake (mg) (FFQ + 6daysDRs)/2

o

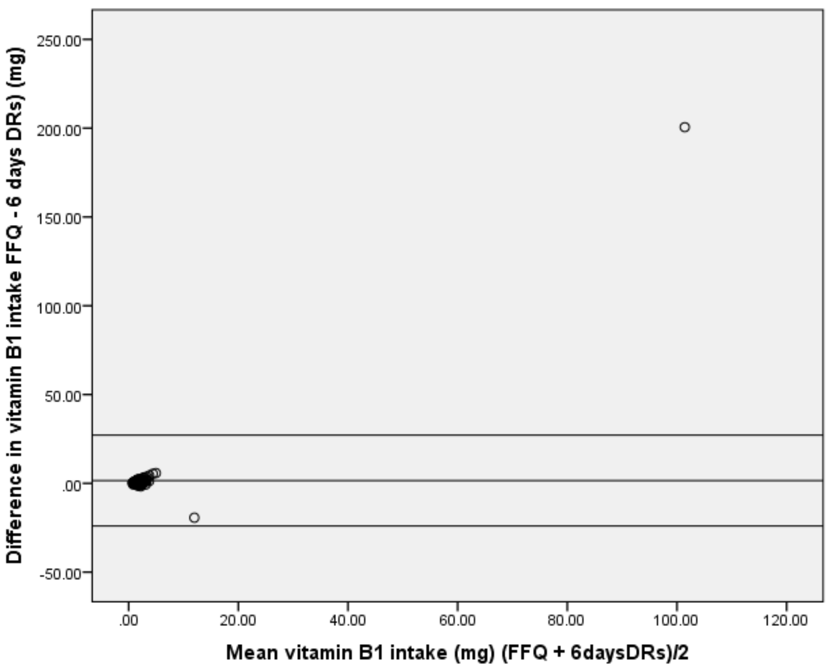
250.00
200.00
150.00
100.00
50.00
.00
-50.00
.00
20.00
40.00
60.00
80.00
100.00
120.00
Difference in vitamin B1 intake FFQ - 6 days DRs) (mg)
Mean vitamin B1 intake (mg) (FFQ + 6daysDRs)/2

p

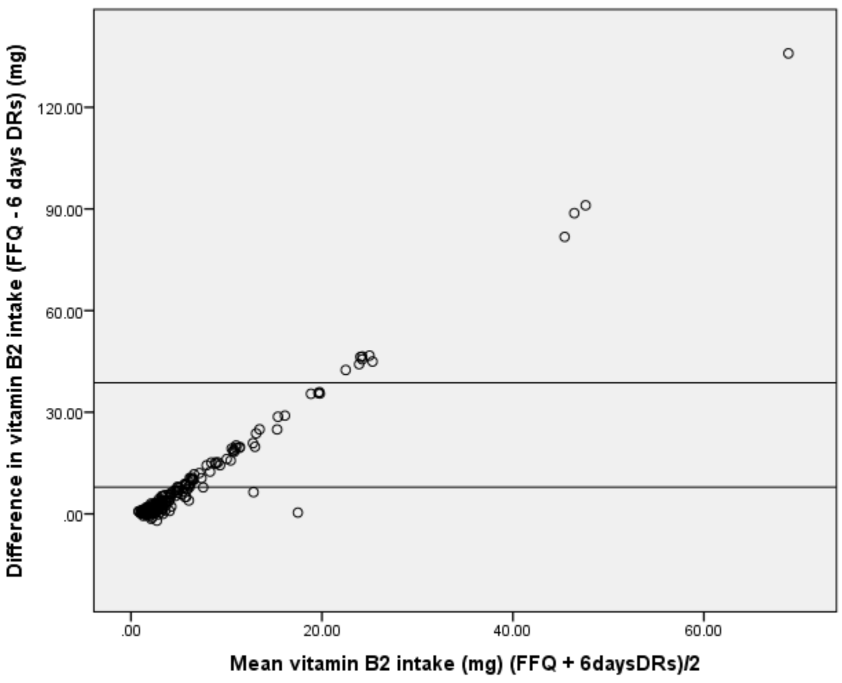
120.00
90.00
60.00
30.00
.00
.00
20.00
40.00
60.00
Difference in vitamin B2 intake (FFQ - 6 days DRs) (mg)
Mean vitamin B2 intake (mg) (FFQ + 6daysDRs)/2

q

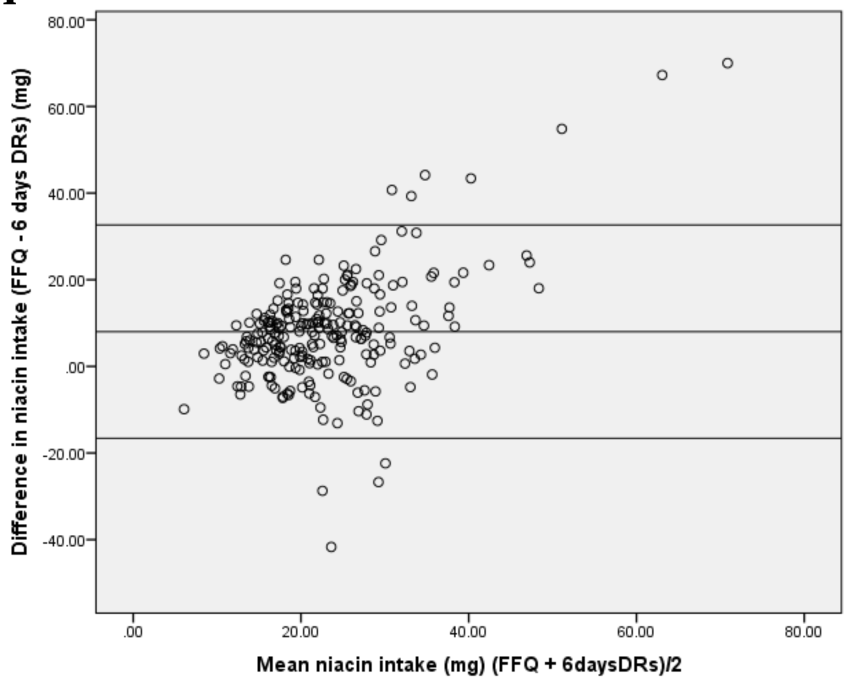
80.00
60.00
40.00
20.00
.00
-20.00
-40.00
Difference in niacin intake (FFQ - 6 days DRs) (mg)
.00
20.00
40.00
60.00
80.00
Mean niacin intake (mg) (FFQ + 6daysDRs)/2

r

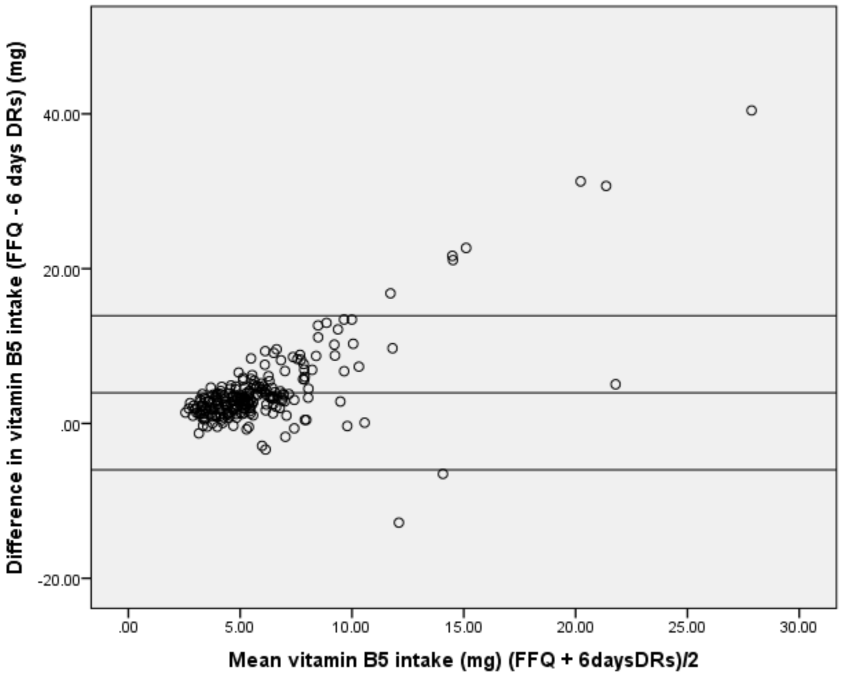
40.00
20.00
.00
-20.00
Difference in vitamin B5 intake (FFQ - 6 days DRs) (mg)
.00
5.00
10.00
15.00
20.00
25.00
30.00
Mean vitamin B5 intake (mg) (FFQ + 6daysDRs)/2

s

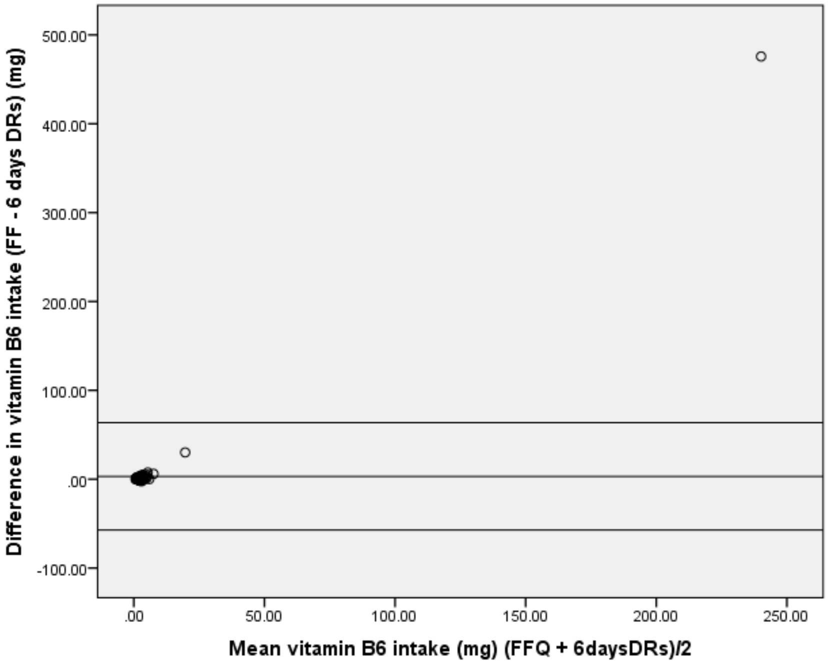
500.00
400.00
300.00
200.00
100.00
.00
-100.00
.00
50.00
100.00
150.00
200.00
250.00
Difference in vitamin B6 intake (FF - 6 days DRs) (mg)
Mean vitamin B6 intake (mg) (FFQ + 6daysDRs)/2

t

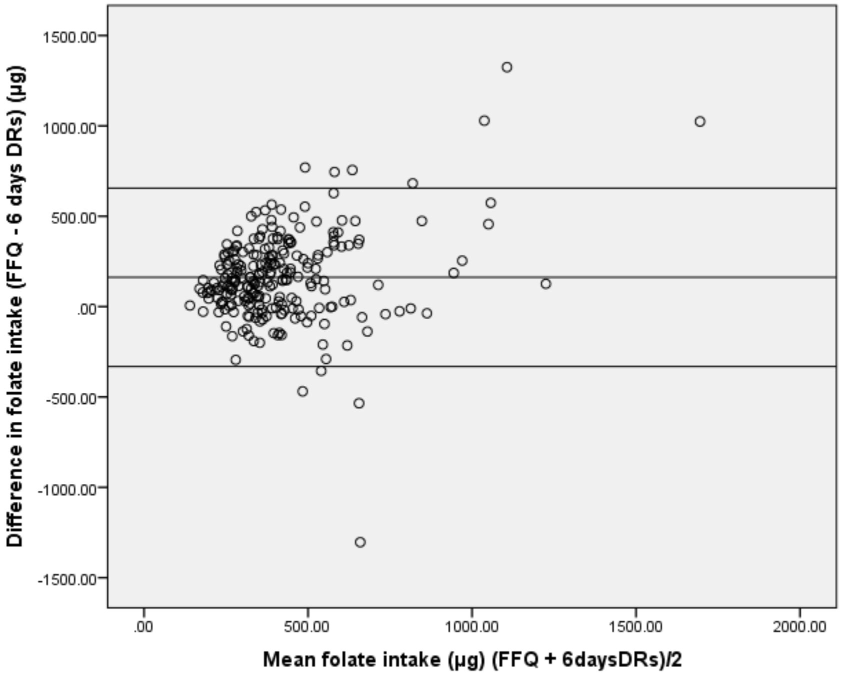
1500.00
1000.00
500.00
.00
-500.00
-1000.00
-1500.00
.00
500.00
1000.00
1500.00
2000.00
Difference in folate intake (FFQ - 6 days DRs) (µg)
Mean folate intake (µg) (FFQ + 6daysDRs)/2

u

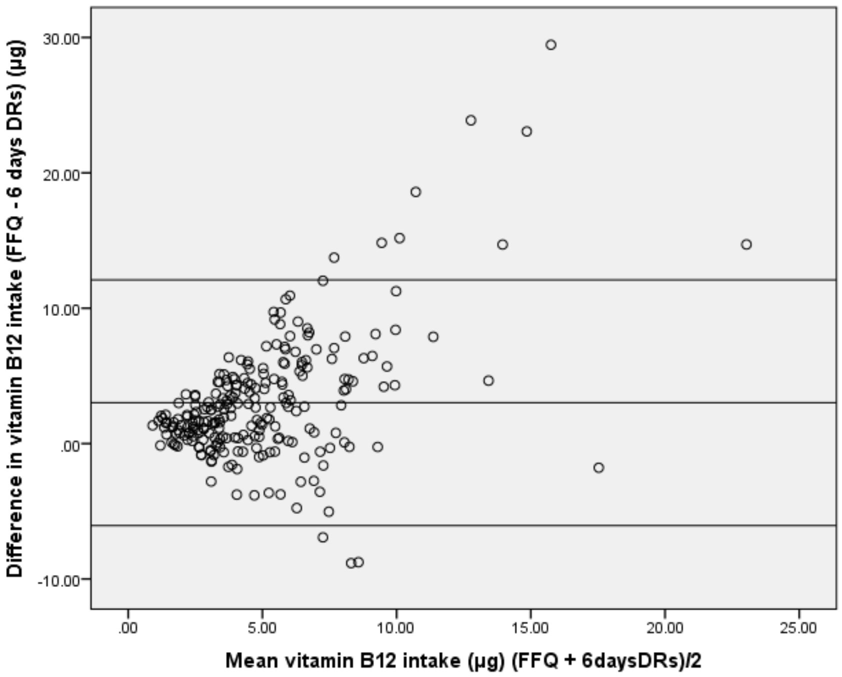
30.00
20.00
10.00
.00
-10.00
Difference in vitamin B12 intake (FFQ - 6 days DRs) (µg)
.00
5.00
10.00
15.00
20.00
25.00
Mean vitamin B12 intake (µg) (FFQ + 6daysDRs)/2

v

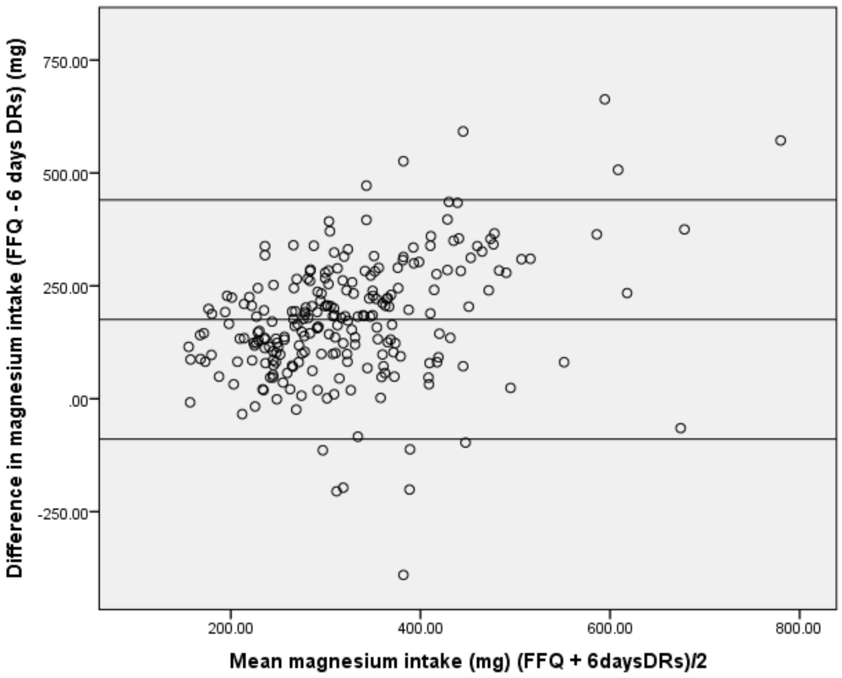
750.00
500.00
250.00
.00
-250.00
Difference in magnesium intake (FFQ - 6 days DRs) (mg)
200.00
400.00
600.00
800.00
Mean magnesium intake (mg) (FFQ + 6daysDRs)/2

W

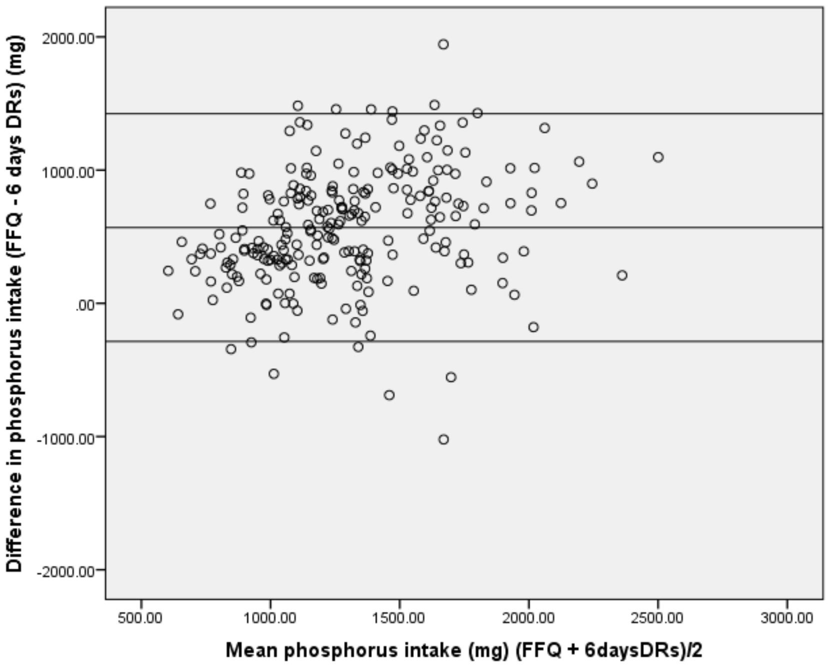
Difference in phosphorus intake (FFQ - 6 days DRs) (mg)
2000.00
1000.00
.00
-1000.00
-2000.00
500.00
1000.00
1500.00
2000.00
2500.00
3000.00
Mean phosphorus intake (mg) (FFQ + 6daysDRs)/2

X

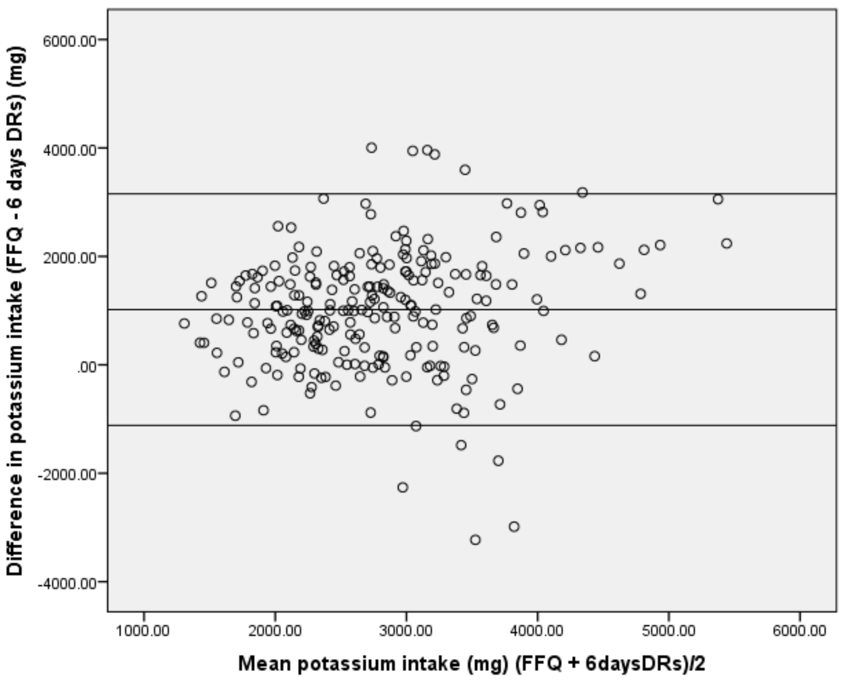
Difference in potassium intake (FFQ - 6 days DRs) (mg)
6000.00
4000.00
2000.00
.00
-2000.00
-4000.00
1000.00
2000.00
3000.00
4000.00
5000.00
6000.00
Mean potassium intake (mg) (FFQ + 6daysDRs)/2

y

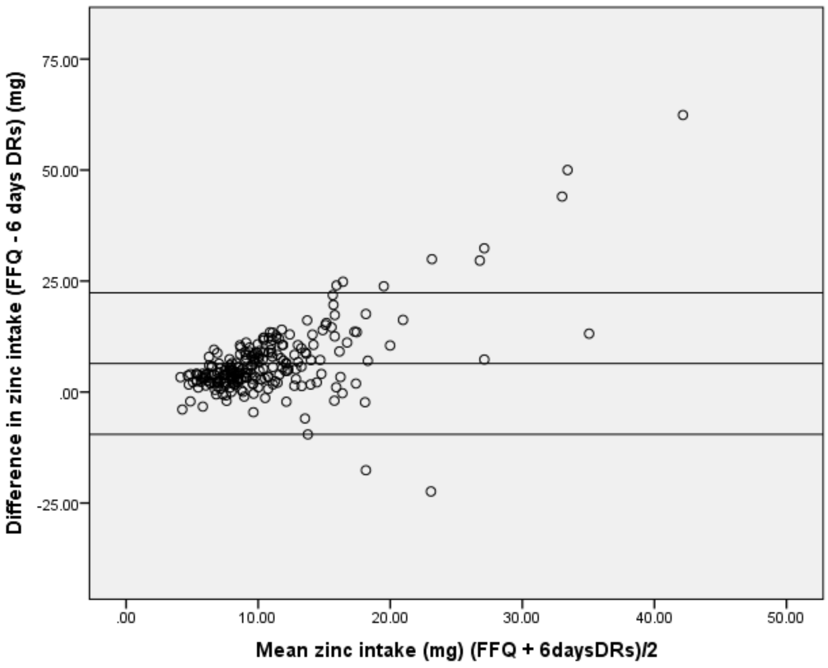
75.00
50.00
25.00
.00
-25.00
Difference in zinc intake (FFQ - 6 days DRs) (mg)
.00
10.00
20.00
30.00
40.00
50.00
Mean zinc intake (mg) (FFQ + 6daysDRs)/2

z

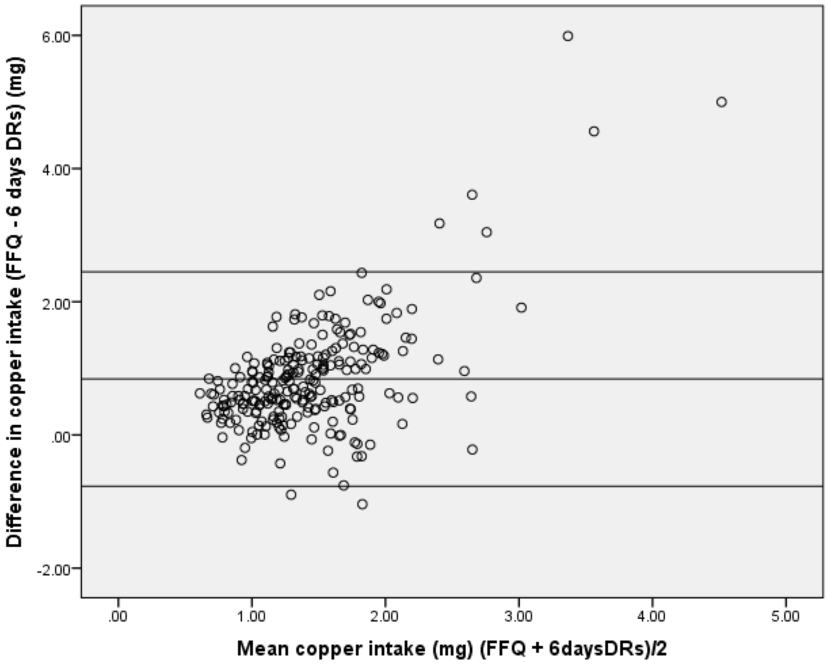
6.00
4.00
2.00
.00
-2.00
Difference in copper intake (FFQ - 6 days DRs) (mg)
.00
1.00
2.00
3.00
4.00
5.00
Mean copper intake (mg) (FFQ + 6daysDRs)/2

aa

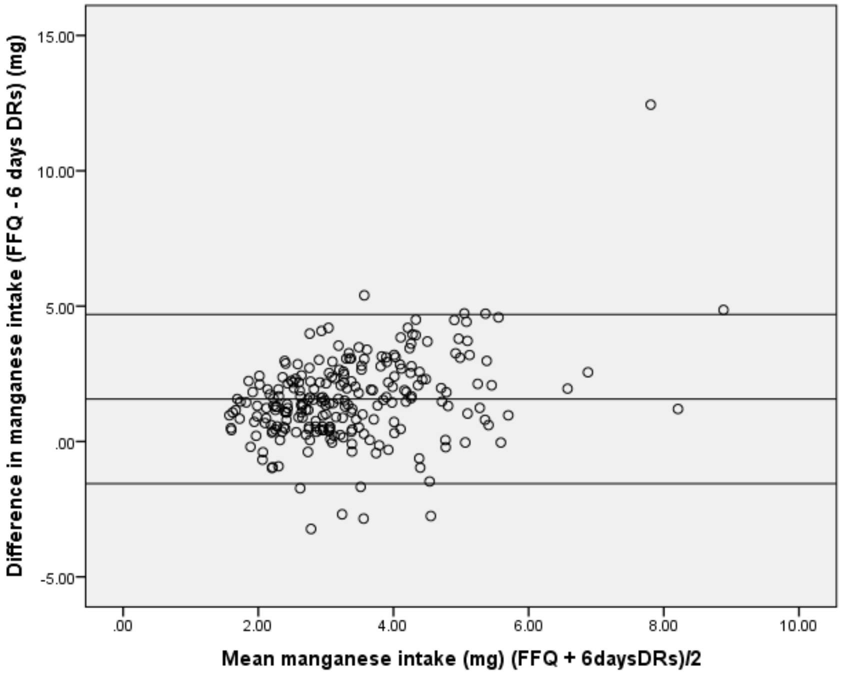
Difference in manganese intake (FFQ - 6 days DRs) (mg)
15.00
10.00
5.00
.00
-5.00
.00
2.00
4.00
6.00
8.00
10.00
Mean manganese intake (mg) (FFQ + 6daysDRs)/2

bb

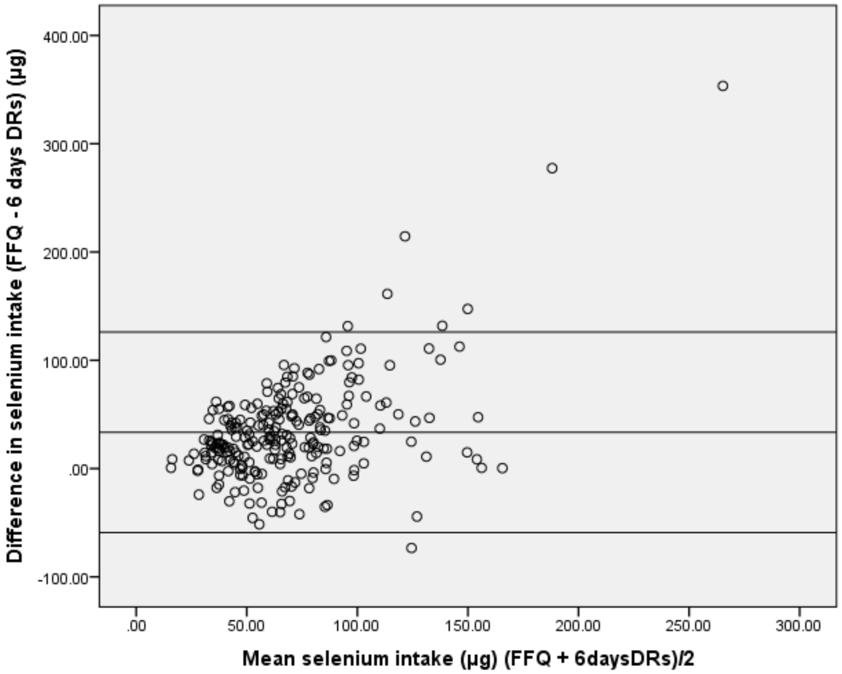
Difference in selenium intake (FFQ - 6 days DRs) (µg)
400.00
300.00
200.00
100.00
.00
-100.00
.00
50.00
100.00
150.00
200.00
250.00
300.00
Mean selenium intake (µg) (FFQ + 6daysDRs)/2

Figure S2 Bland-Altman plots of difference between **a**, carbohydrates (grams) **b** protein (grams), **c** fat (grams), **d** SFA (% of TEI), **e**, SFA (grams), **f** MUFA (% of TEI) **g** MUFA (grams), **h** PUFA (% of TEI), **i** PUFA (grams), **j** sugars (% of carbohydrates), **k** sugars (grams), **l** alcohol (grams), **m** vitamin A, **n** vitamin E, **o** vitamin B1, **p** vitamin B2, **q** niacin, **r** vitamin B5, **s** vitamin B6, **t** vitamin B9, **u** vitamin B12, **v** magnesium, **w** phosphorus, **x** potassium, **y** zinc, **z** copper, **aa** manganese, **bb** and selenium, as predicted by the first and second FFQs (n=52)

a

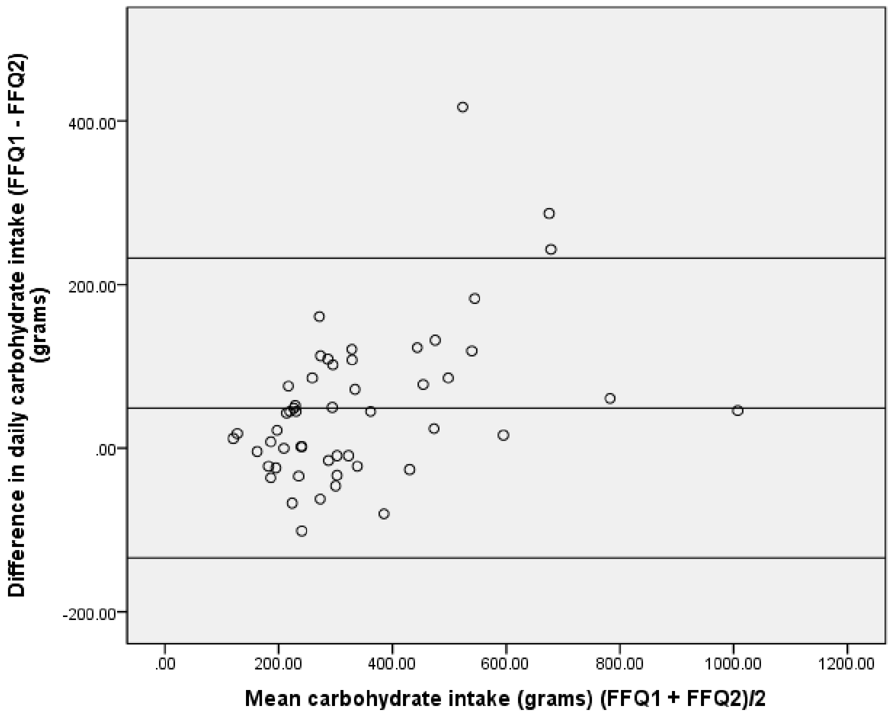

b

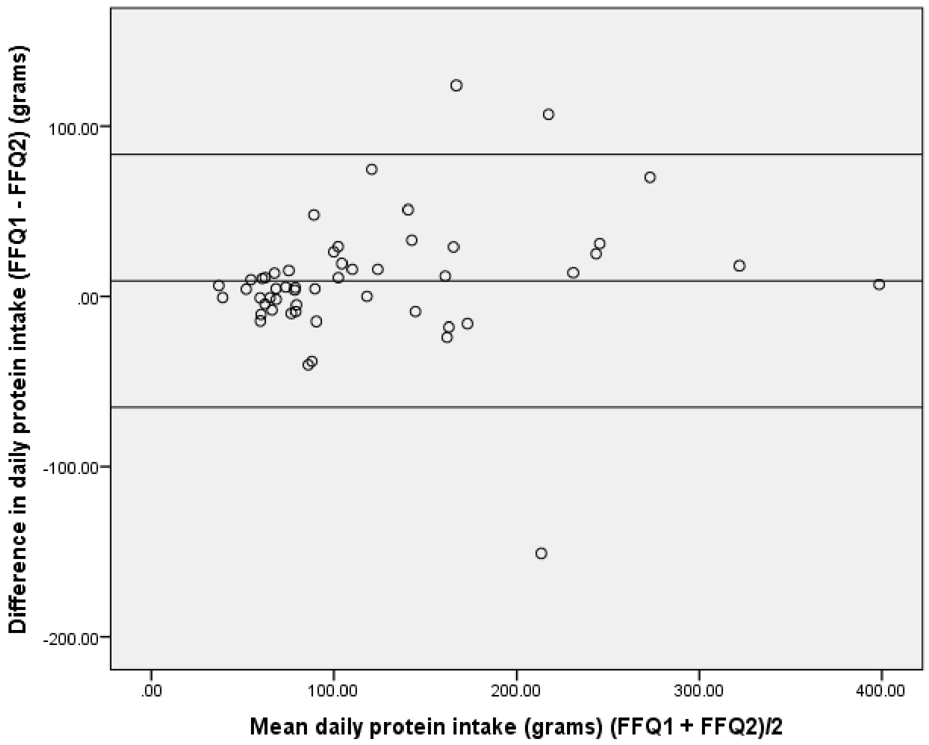
Difference in daily protein intake (FFQ1 - FFQ2) (grams)
100.00
.00
-100.00
-200.00
.00
100.00
200.00
300.00
400.00
Mean daily protein intake (grams) (FFQ1 + FFQ2)/2

c

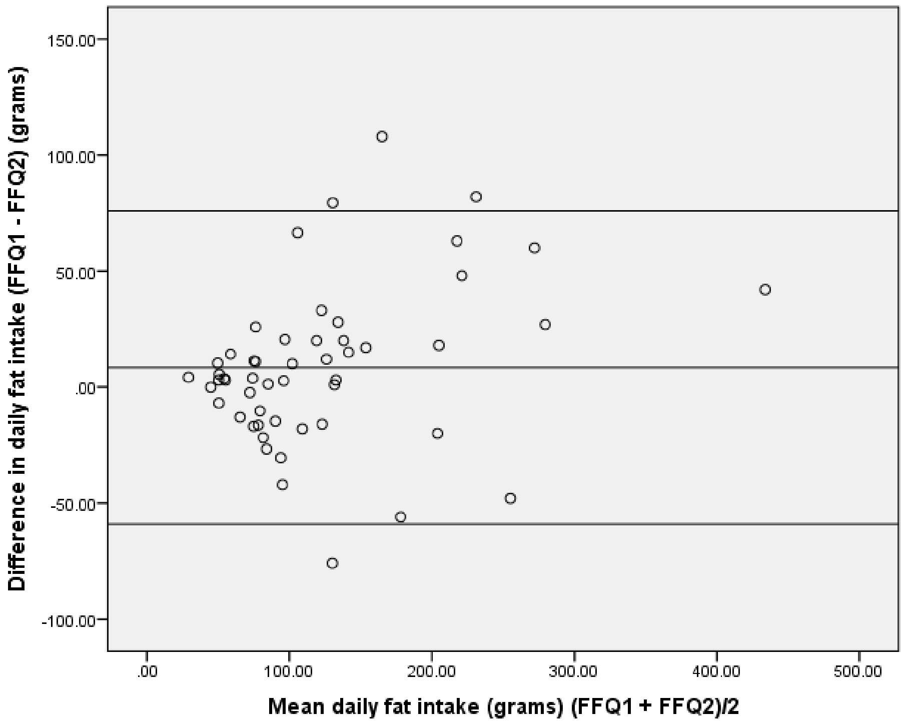
Difference in daily fat intake (FFQ1 - FFQ2) (grams)
150.00
100.00
50.00
.00
-50.00
-100.00
.00
100.00
200.00
300.00
400.00
500.00
Mean daily fat intake (grams) (FFQ1 + FFQ2)/2

d

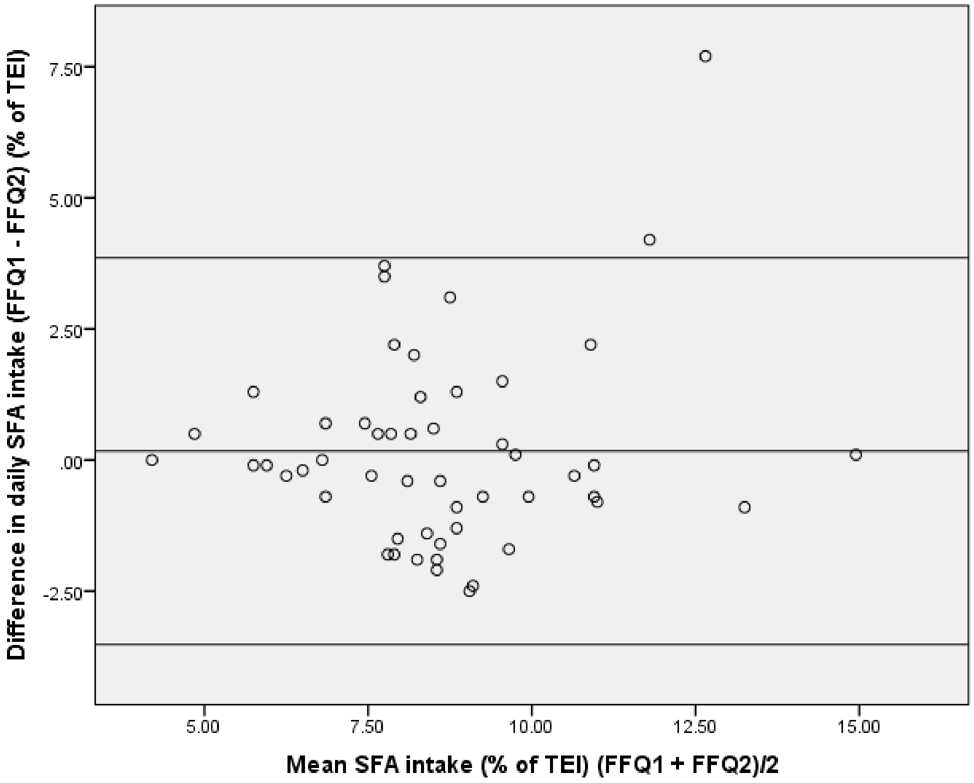

Difference in daily SFA intake (FFQ1 - FFQ2) (% of TEI)
7.50
5.00
2.50
.00
-2.50
5.00
7.50
10.00
12.50
15.00
Mean SFA intake (% of TEI) (FFQ1 + FFQ2)/2

e

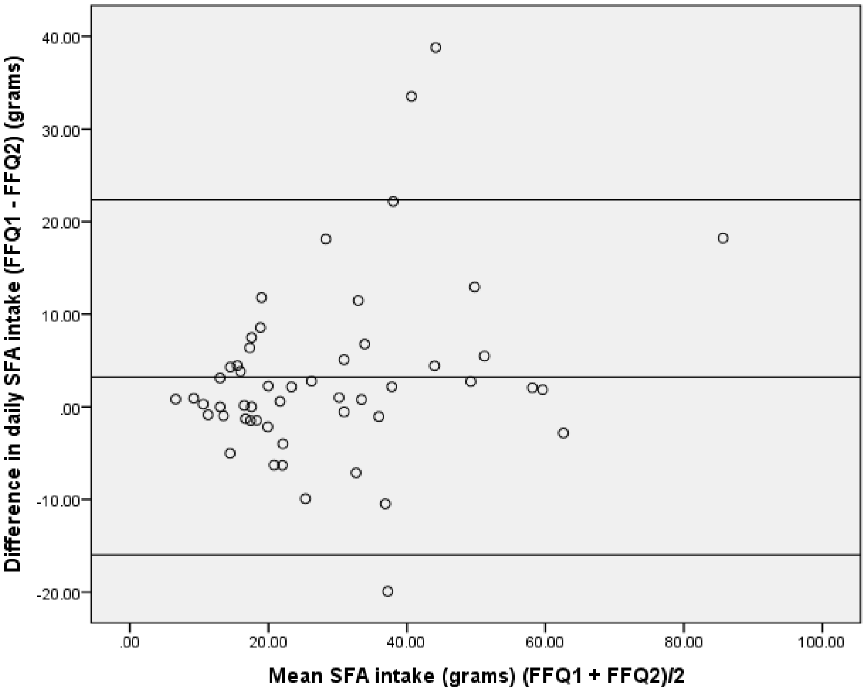

Difference in daily SFA intake (FFQ1 - FFQ2) (grams)
40.00
30.00
20.00
10.00
.00
-10.00
-20.00
.00
20.00
40.00
60.00
80.00
100.00
Mean SFA intake (grams) (FFQ1 + FFQ2)/2

f

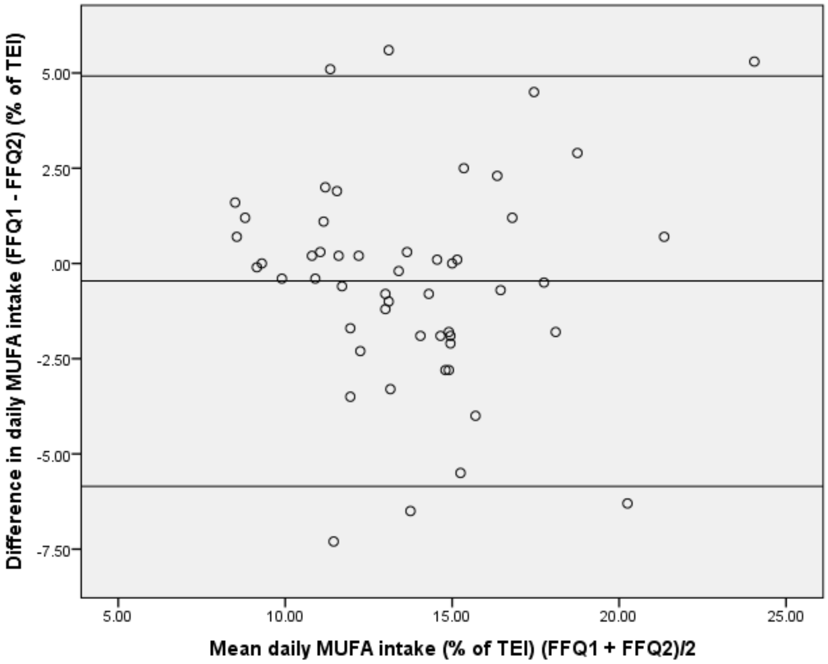
Difference in daily MUFA intake (FFQ1 - FFQ2) (% of TEI)
5.00
2.50
.00
-2.50
-5.00
-7.50
5.00
10.00
15.00
20.00
25.00
Mean daily MUFA intake (% of TEI) (FFQ1 + FFQ2)/2

g

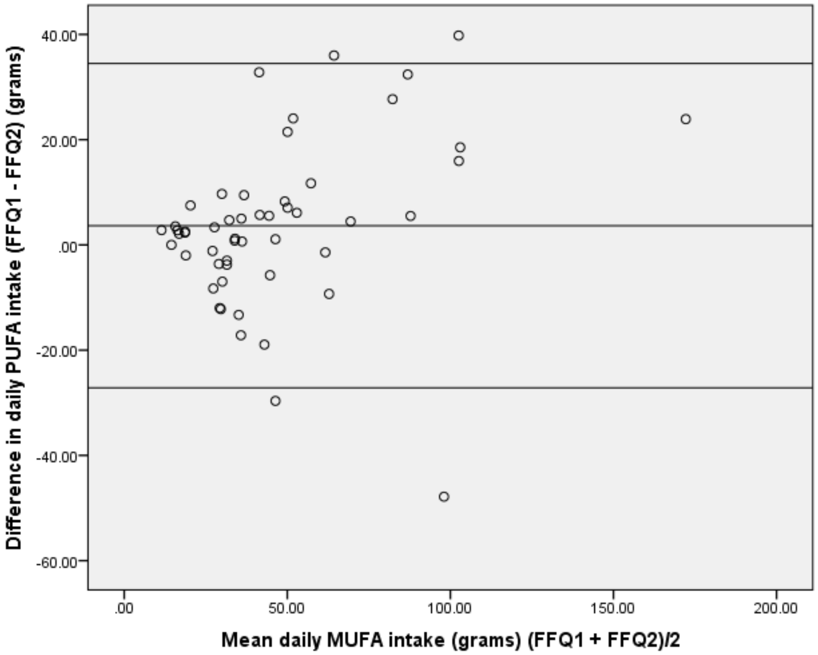
Difference in daily PUFA intake (FFQ1 - FFQ2) (grams)
40.00
20.00
.00
-20.00
-40.00
-60.00
.00
50.00
100.00
150.00
200.00
Mean daily MUFA intake (grams) (FFQ1 + FFQ2)/2

h

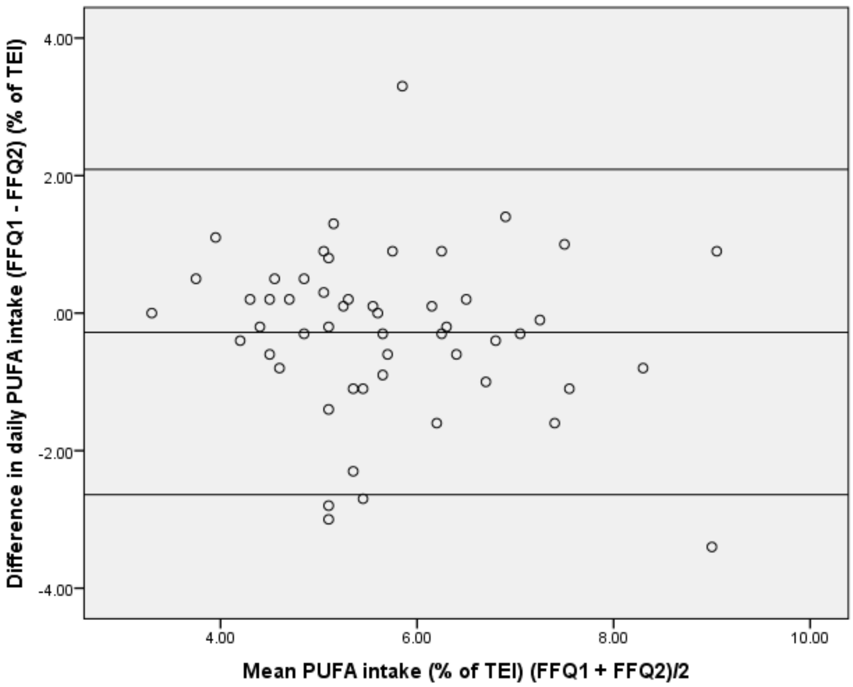
4.00
2.00
.00
-2.00
-4.00
4.00
6.00
8.00
10.00
Difference in daily PUFA intake (FFQ1 - FFQ2) (% of TEI)
Mean PUFA intake (% of TEI) (FFQ1 + FFQ2)/2

i

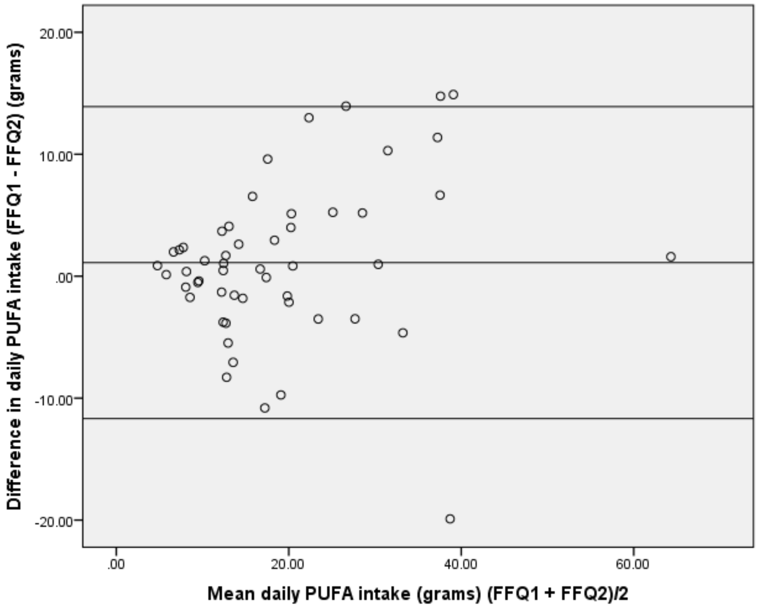
20.00
10.00
.00
-10.00
-20.00
.00
20.00
40.00
60.00
Difference in daily PUFA intake (FFQ1 - FFQ2) (grams)
Mean daily PUFA intake (grams) (FFQ1 + FFQ2)/2

j

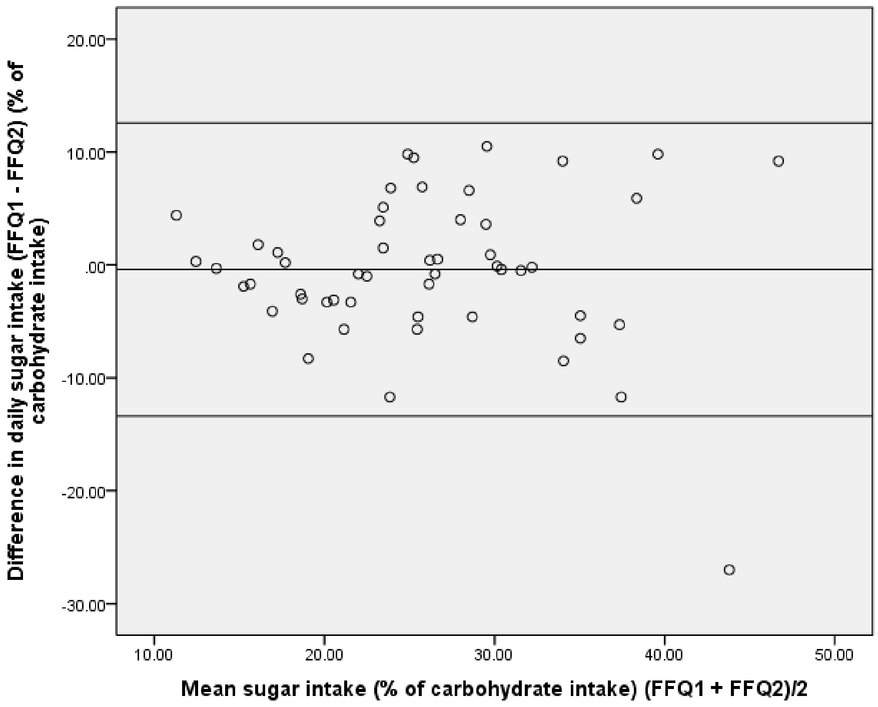
20.00
10.00
.00
-10.00
-20.00
-30.00
10.00
20.00
30.00
40.00
50.00
Difference in daily sugar intake (FFQ1 - FFQ2) (% of carbohydrate intake)
Mean sugar intake (% of carbohydrate intake) (FFQ1 + FFQ2)/2

k

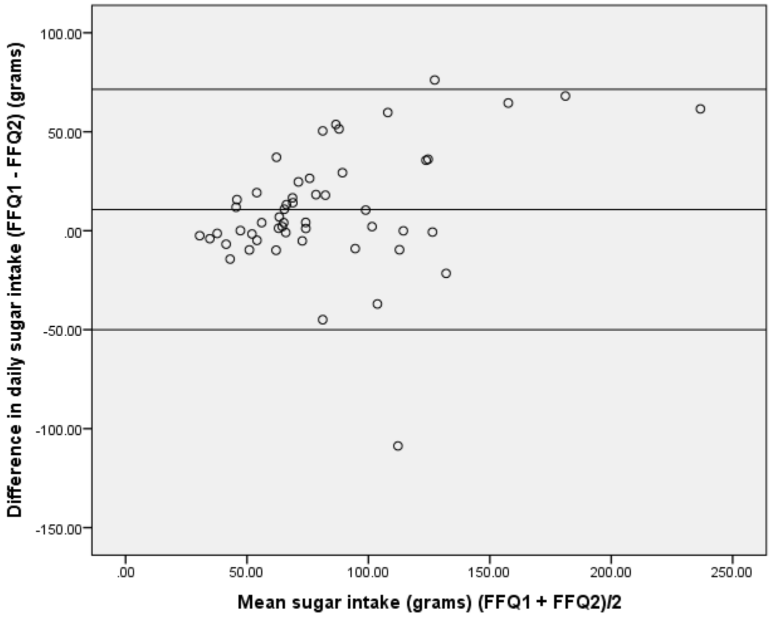
100.00
50.00
.00
-50.00
-100.00
-150.00
.00
50.00
100.00
150.00
200.00
250.00
Difference in daily sugar intake (FFQ1 - FFQ2) (grams)
Mean sugar intake (grams) (FFQ1 + FFQ2)/2

l

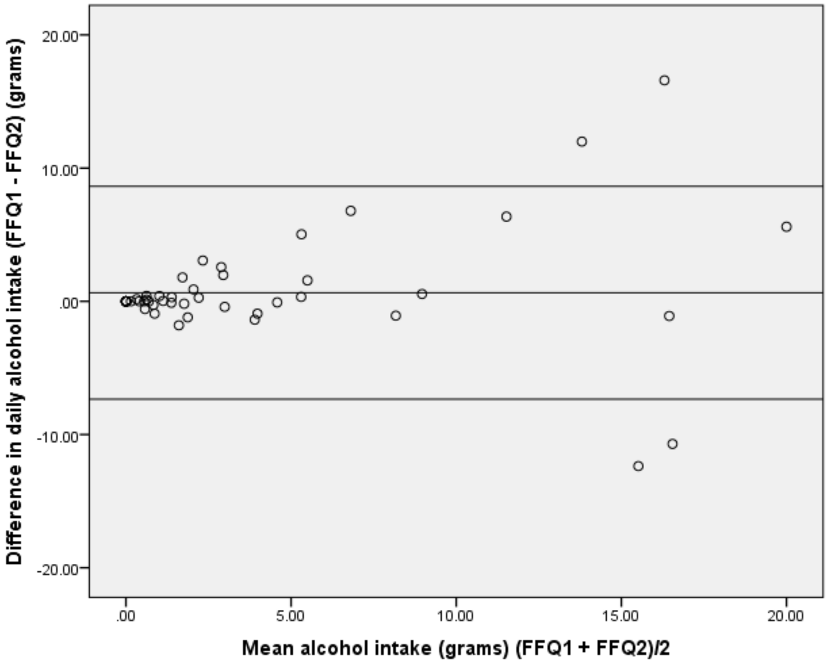
Difference in daily alcohol intake (FFQ1 - FFQ2) (grams)
20.00
10.00
.00
-10.00
-20.00
.00
5.00
10.00
15.00
20.00
Mean alcohol intake (grams) (FFQ1 + FFQ2)/2

m

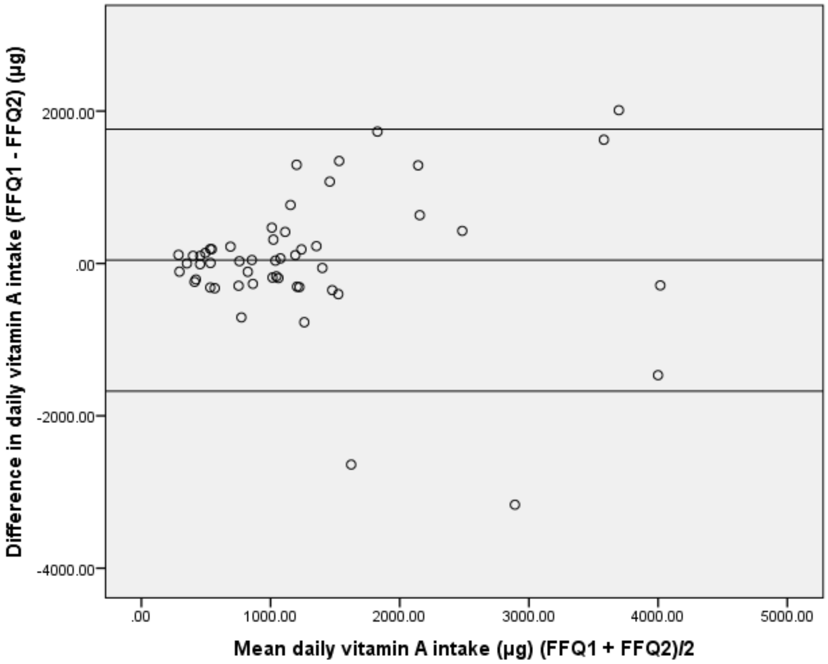
Difference in daily vitamin A intake (FFQ1 - FFQ2) (µg)
2000.00
.00
-2000.00
-4000.00
.00
1000.00
2000.00
3000.00
4000.00
5000.00
Mean daily vitamin A intake (µg) (FFQ1 + FFQ2)/2

n

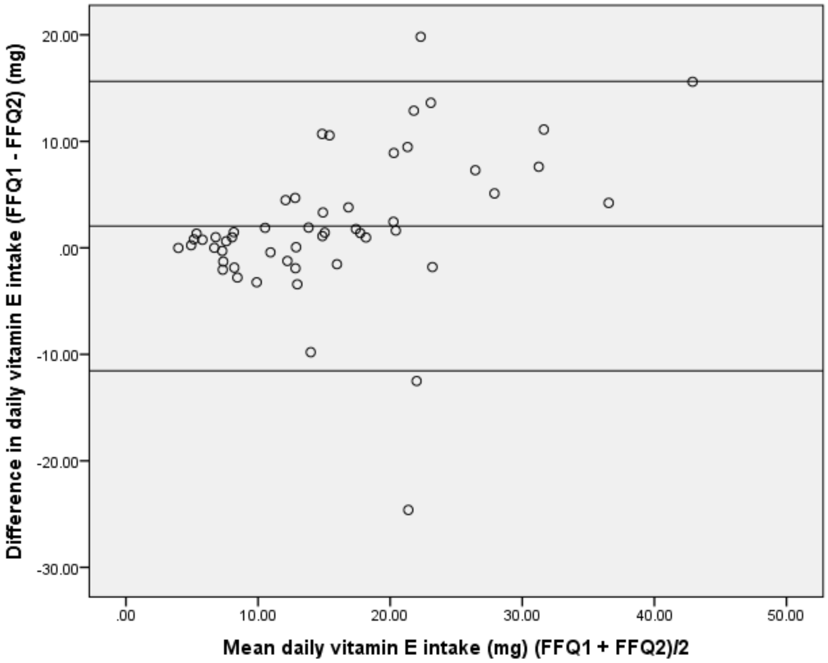
Difference in daily vitamin E intake (FFQ1 - FFQ2) (mg)
20.00
10.00
.00
-10.00
-20.00
-30.00
.00
10.00
20.00
30.00
40.00
50.00
Mean daily vitamin E intake (mg) (FFQ1 + FFQ2)/2

o

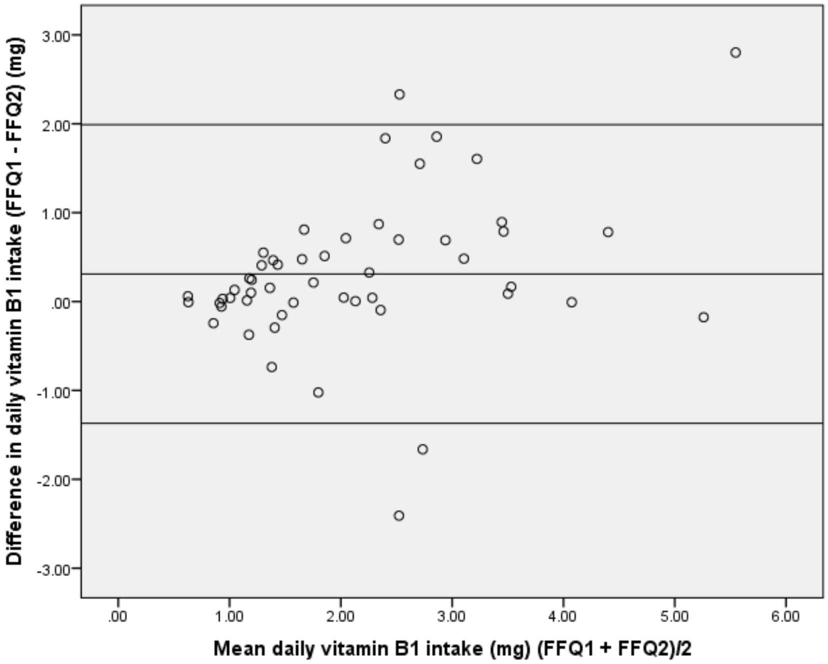
Difference in daily vitamin B1 intake (FFQ1 - FFQ2) (mg)
3.00
2.00
1.00
.00
-1.00
-2.00
-3.00
.00
1.00
2.00
3.00
4.00
5.00
6.00
Mean daily vitamin B1 intake (mg) (FFQ1 + FFQ2)/2

p

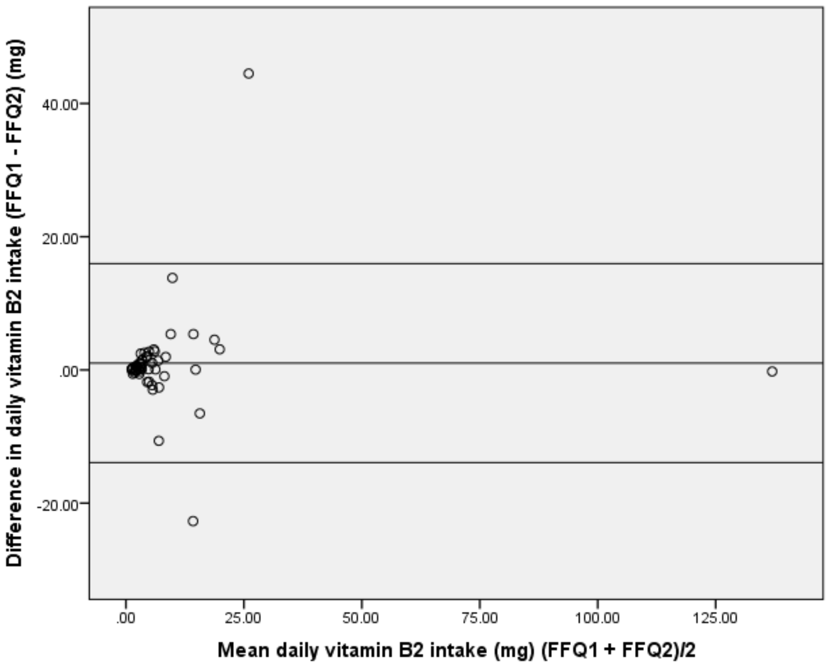
Difference in daily vitamin B2 intake (FFQ1 - FFQ2) (mg)
40.00
20.00
.00
-20.00
.00
25.00
50.00
75.00
100.00
125.00
Mean daily vitamin B2 intake (mg) (FFQ1 + FFQ2)/2

q

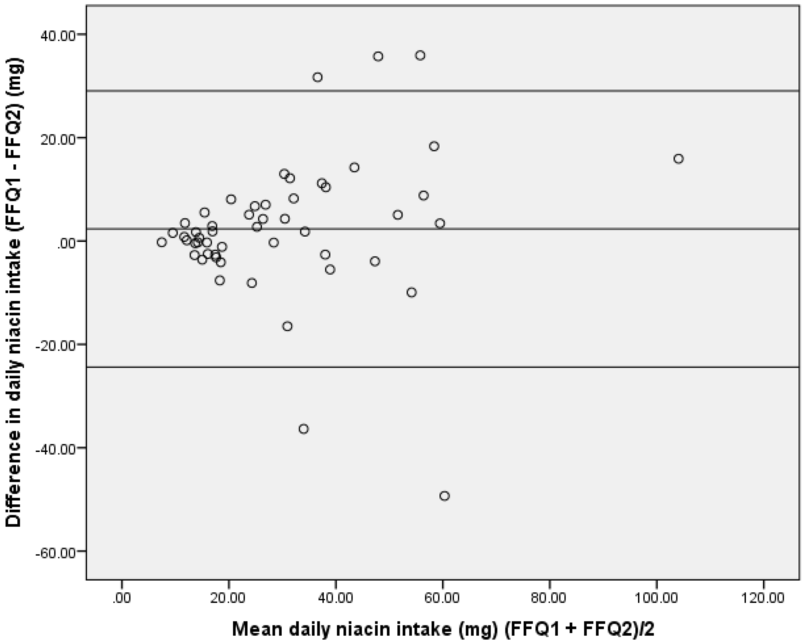
Difference in daily niacin intake (FFQ1 - FFQ2) (mg)
40.00
20.00
.00
-20.00
-40.00
-60.00
.00
20.00
40.00
60.00
80.00
100.00
120.00
Mean daily niacin intake (mg) (FFQ1 + FFQ2)/2

r

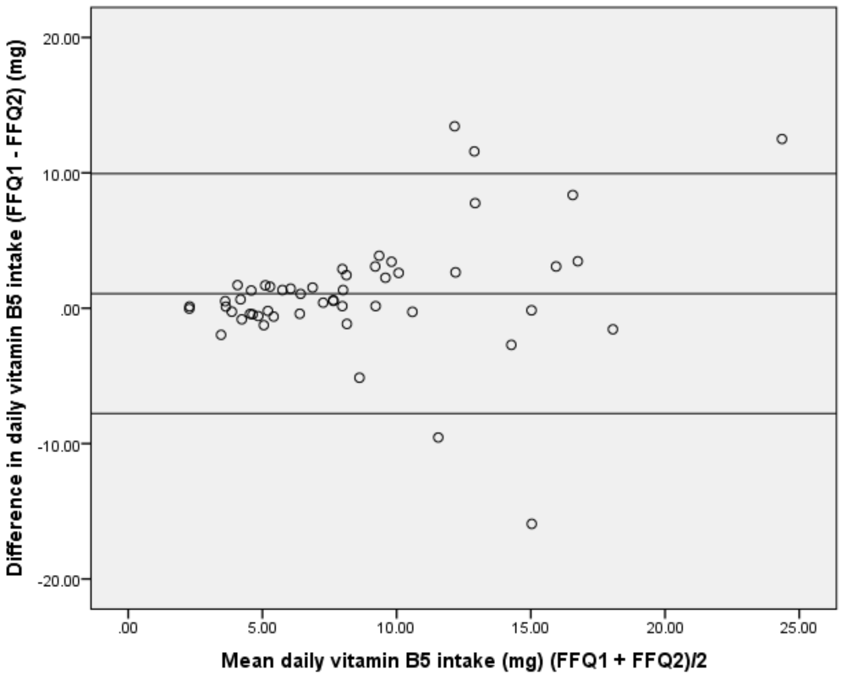
20.00
10.00
.00
-10.00
-20.00
.00
5.00
10.00
15.00
20.00
25.00
Difference in daily vitamin B5 intake (FFQ1 - FFQ2) (mg)
Mean daily vitamin B5 intake (mg) (FFQ1 + FFQ2)/2

s

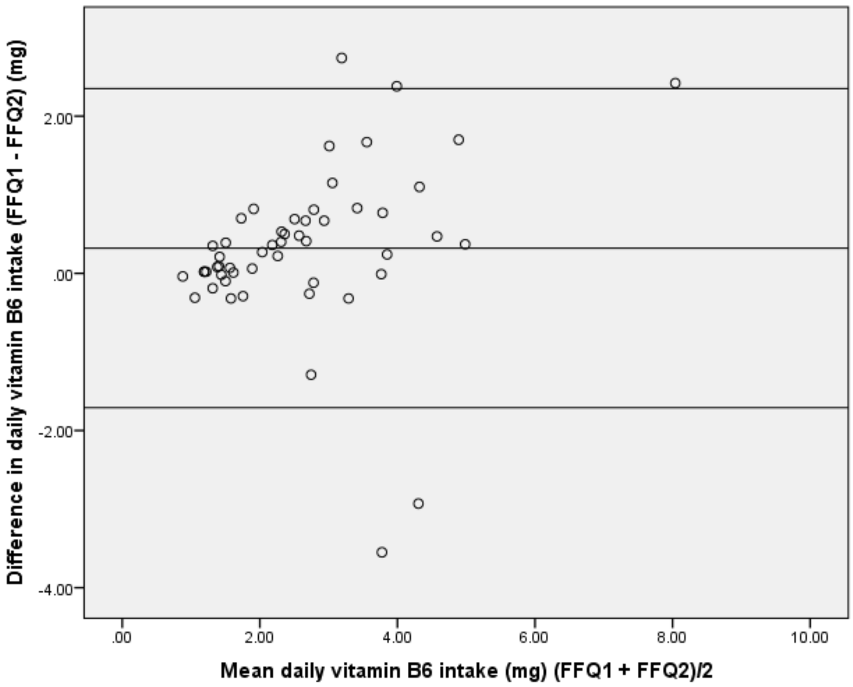
2.00
.00
-2.00
-4.00
.00
2.00
4.00
6.00
8.00
10.00
Difference in daily vitamin B6 intake (FFQ1 - FFQ2) (mg)
Mean daily vitamin B6 intake (mg) (FFQ1 + FFQ2)/2

t

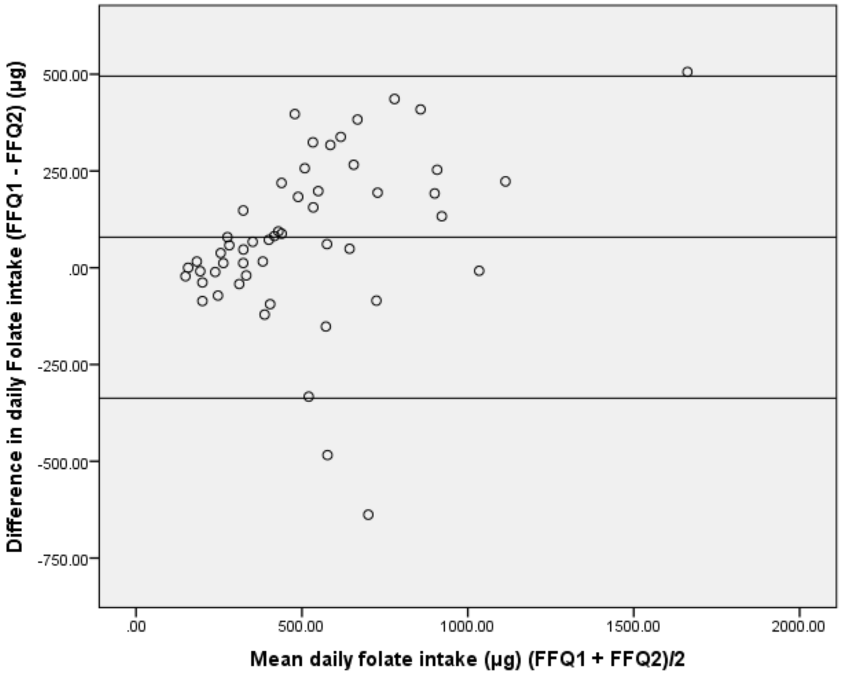
Difference in daily Folate intake (FFQ1 - FFQ2) (µg)
500.00
250.00
.00
-250.00
-500.00
-750.00
.00
500.00
1000.00
1500.00
2000.00
Mean daily folate intake (µg) (FFQ1 + FFQ2)/2

u

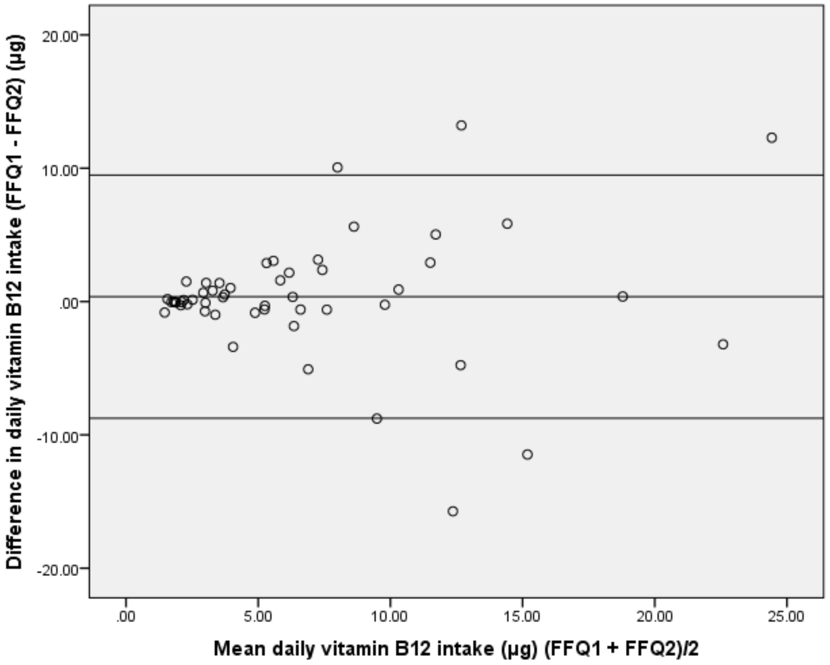
Difference in daily vitamin B12 intake (FFQ1 - FFQ2) (µg)
20.00
10.00
.00
-10.00
-20.00
.00
5.00
10.00
15.00
20.00
25.00
Mean daily vitamin B12 intake (µg) (FFQ1 + FFQ2)/2

V

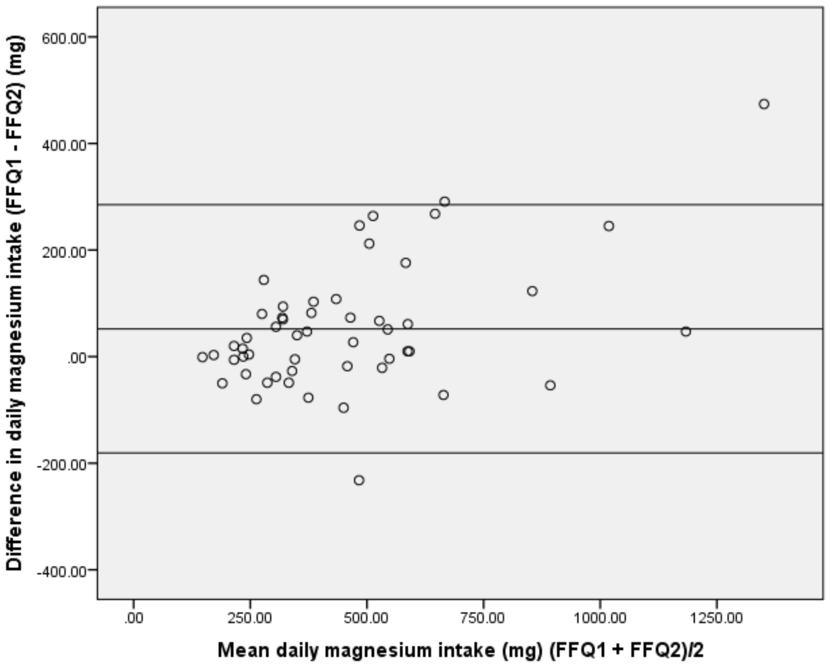
600.00
400.00
200.00
.00
-200.00
-400.00
.00
250.00
500.00
750.00
1000.00
1250.00
Difference in daily magnesium intake (FFQ1 - FFQ2) (mg)
Mean daily magnesium intake (mg) (FFQ1 + FFQ2)/2

W

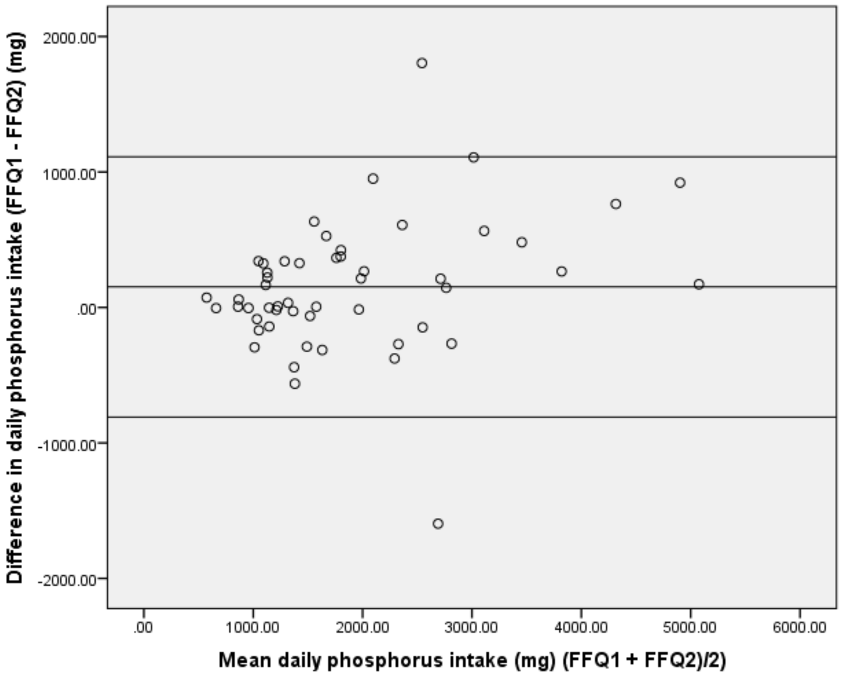
2000.00
1000.00
.00
-1000.00
-2000.00
.00
1000.00
2000.00
3000.00
4000.00
5000.00
6000.00
Difference in daily phosphorus intake (FFQ1 - FFQ2) (mg)
Mean daily phosphorus intake (mg) (FFQ1 + FFQ2)/2)

x

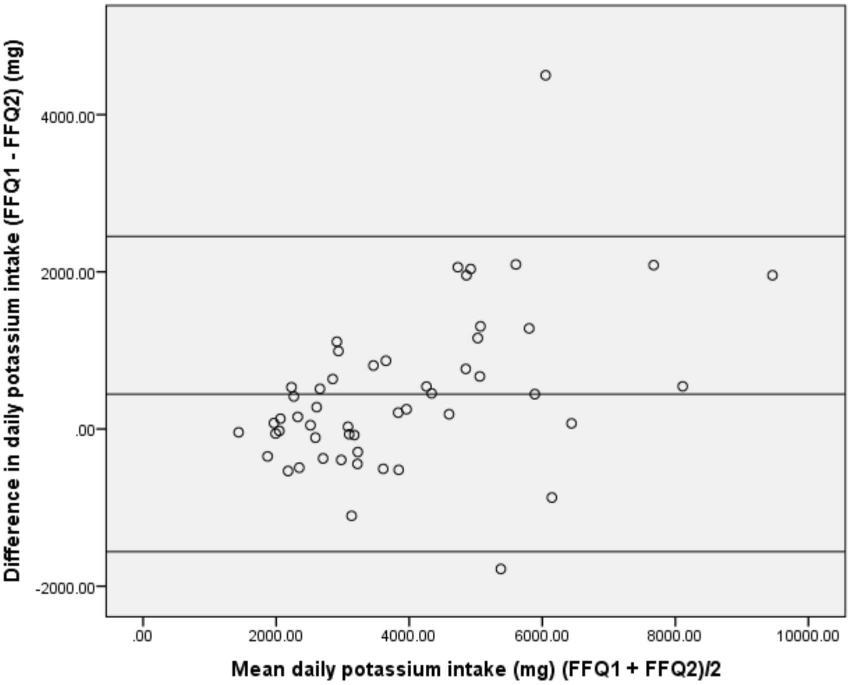
Difference in daily potassium intake (FFQ1 - FFQ2) (mg)
4000.00
2000.00
.00
-2000.00
.00
2000.00
4000.00
6000.00
8000.00
10000.00
Mean daily potassium intake (mg) (FFQ1 + FFQ2)/2

y

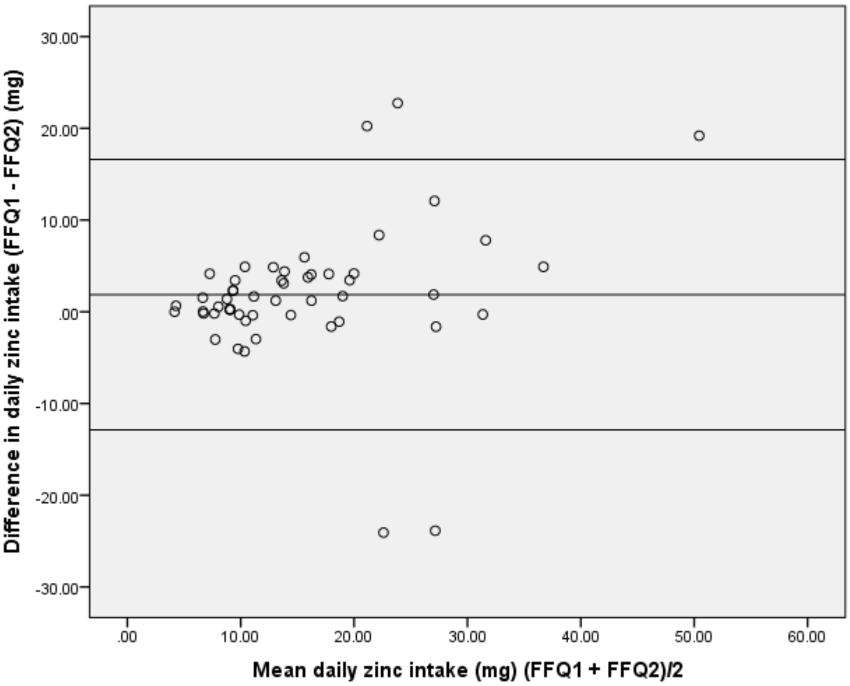
Difference in daily zinc intake (FFQ1 - FFQ2) (mg)
30.00
20.00
10.00
.00
-10.00
-20.00
-30.00
.00
10.00
20.00
30.00
40.00
50.00
60.00
Mean daily zinc intake (mg) (FFQ1 + FFQ2)/2

z

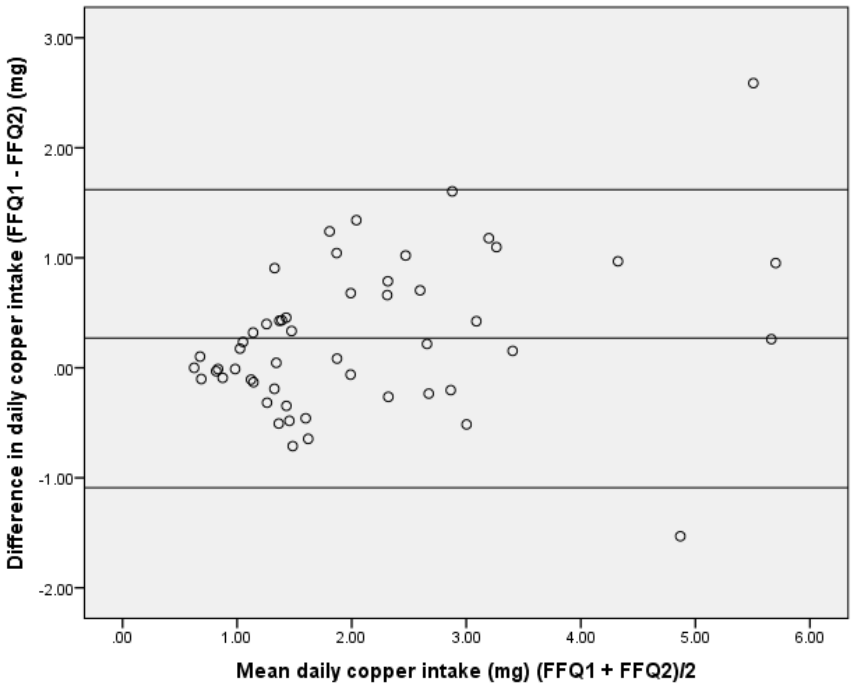
Difference in daily copper intake (FFQ1 - FFQ2) (mg)
3.00
2.00
1.00
.00
-1.00
-2.00
.00
1.00
2.00
3.00
4.00
5.00
6.00
Mean daily copper intake (mg) (FFQ1 + FFQ2)/2

aa

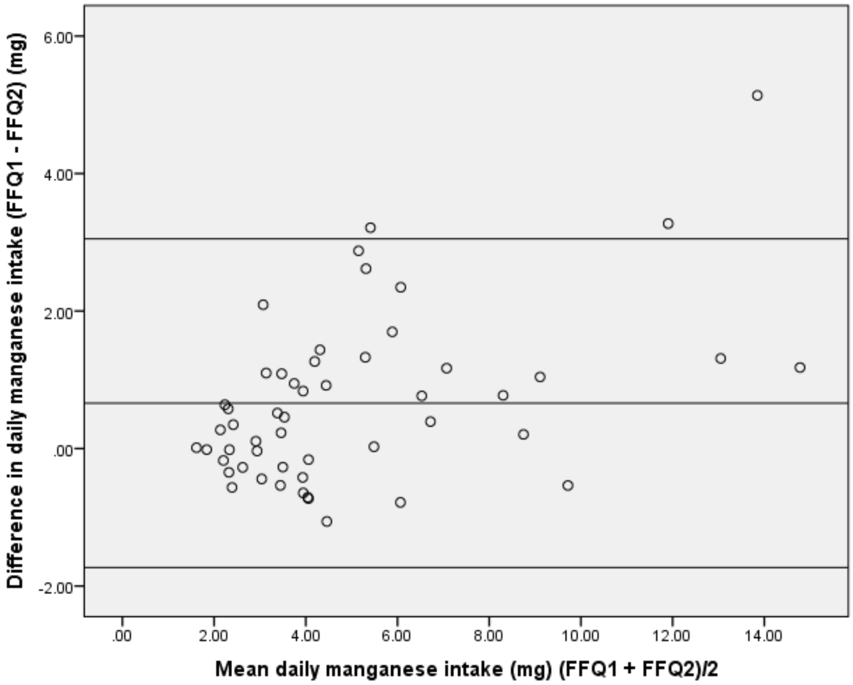
Difference in daily manganese intake (FFQ1 - FFQ2) (mg)
6.00
4.00
2.00
.00
-2.00
.00
2.00
4.00
6.00
8.00
10.00
12.00
14.00
Mean daily manganese intake (mg) (FFQ1 + FFQ2)/2

bb

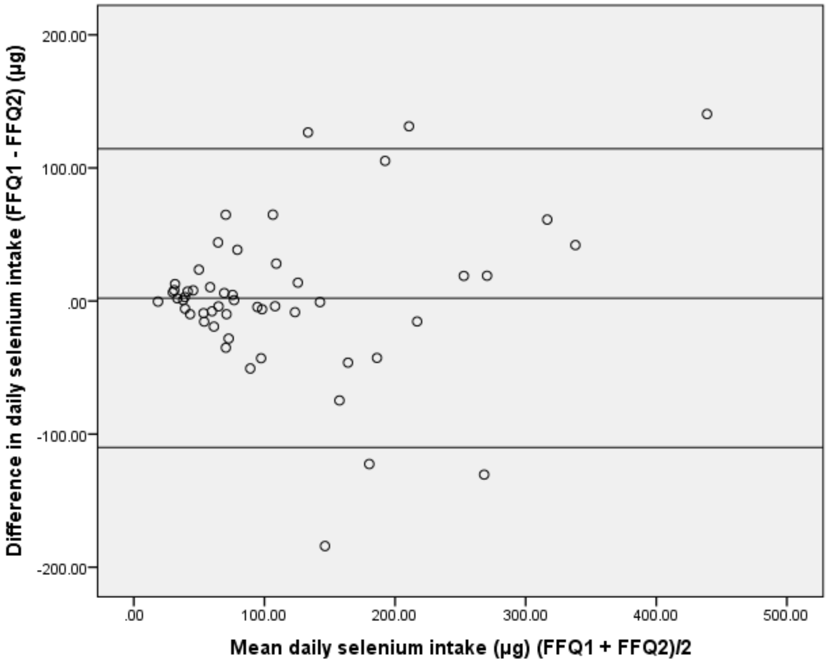
200.00
100.00
.00
-100.00
-200.00
Difference in daily selenium intake (FFQ1 - FFQ2) (µg)
.00
100.00
200.00
300.00
400.00
500.00
Mean daily selenium intake (µg) (FFQ1 + FFQ2)/2
